# Supplementary figures and images for: The development of early human lymphatic vessels as characterized by lymphatic endothelial markers
Source: EMBO J. 2024 Feb 14;43(5):868–85. doi: 10.1038/s44318-024-00045-0 (PMC10907744; doi:10.1038/s44318-024-00045-0)

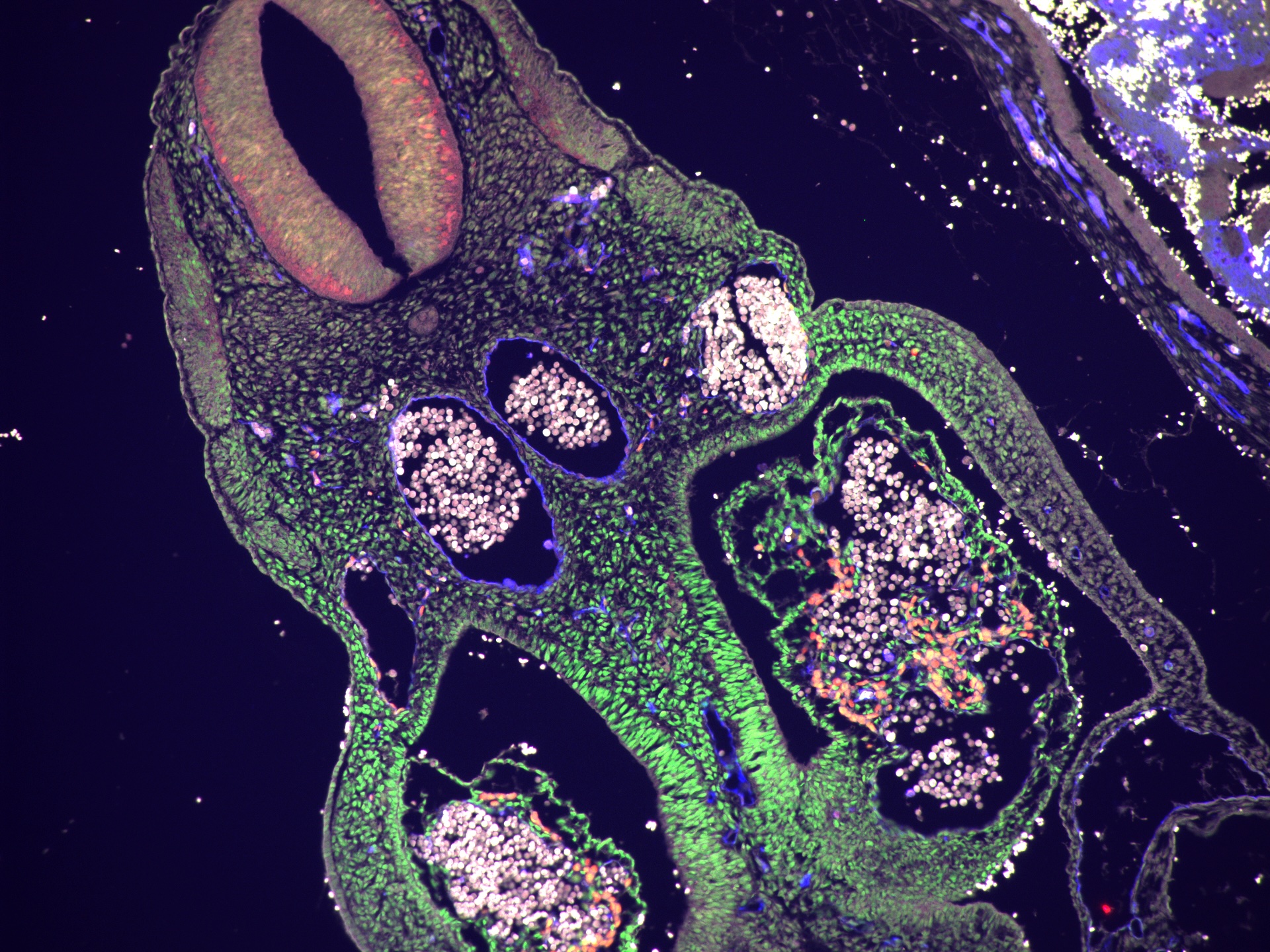

Supplement: Supplementary file 1 — Source Data Fig. 1 [file 44318_2024_45_MOESM1_ESM.zip › Figure1/Figure 1A.jpeg]

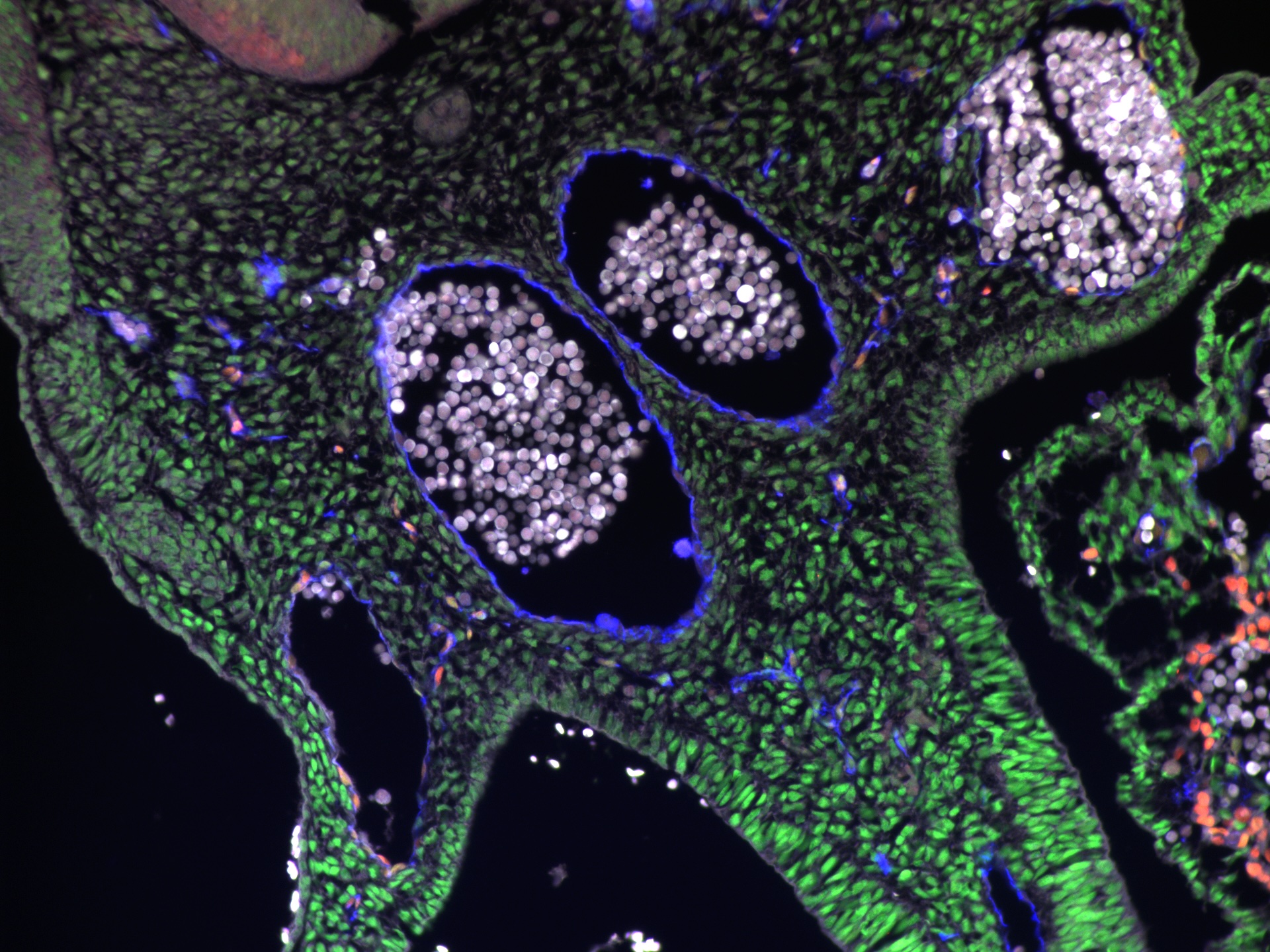

Supplement: Supplementary file 1 — Source Data Fig. 1 [file 44318_2024_45_MOESM1_ESM.zip › Figure1/Figure 1B.jpeg]

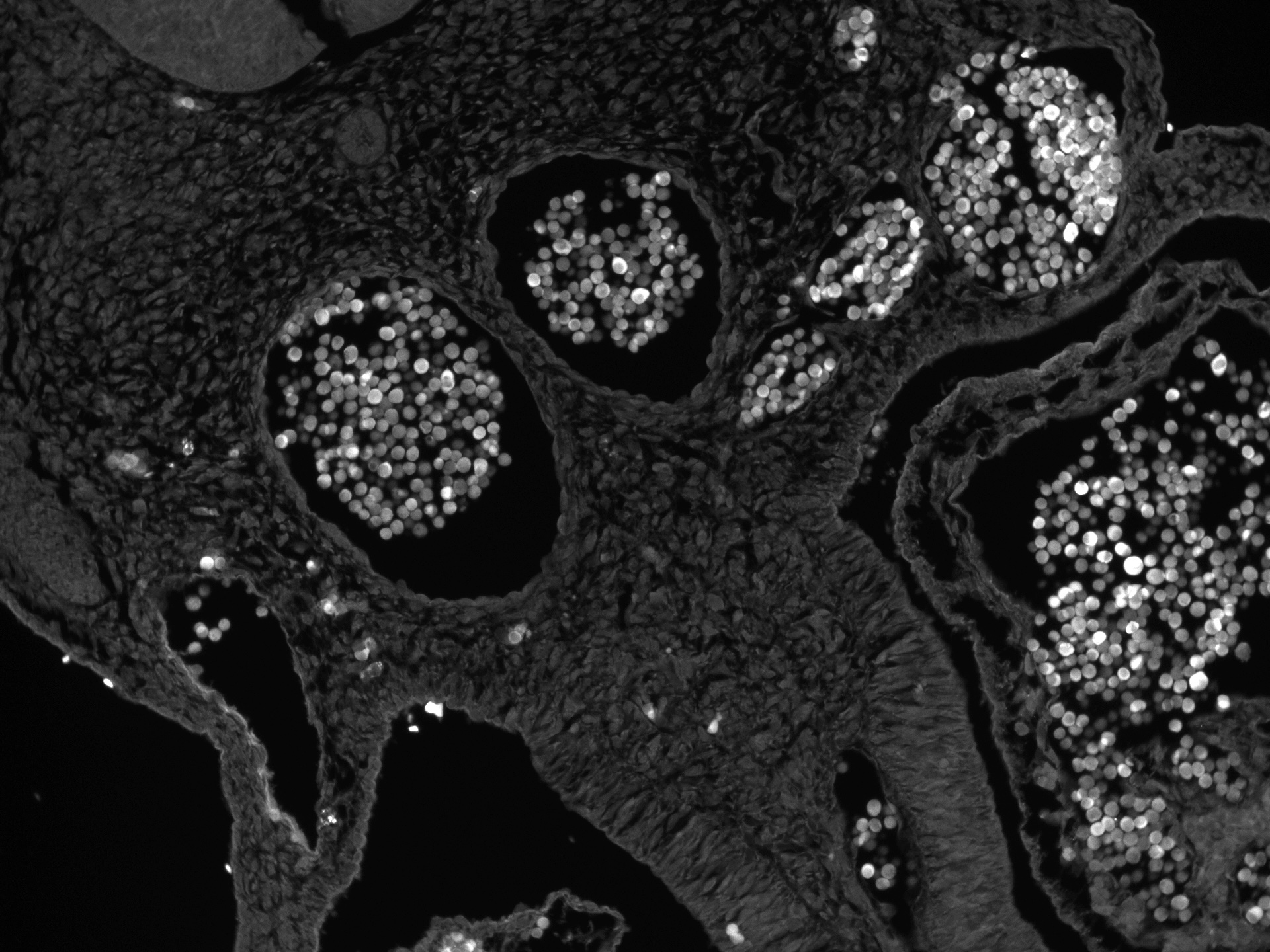

Supplement: Supplementary file 1 — Source Data Fig. 1 [file 44318_2024_45_MOESM1_ESM.zip › Figure1/Figure 1C.jpeg]

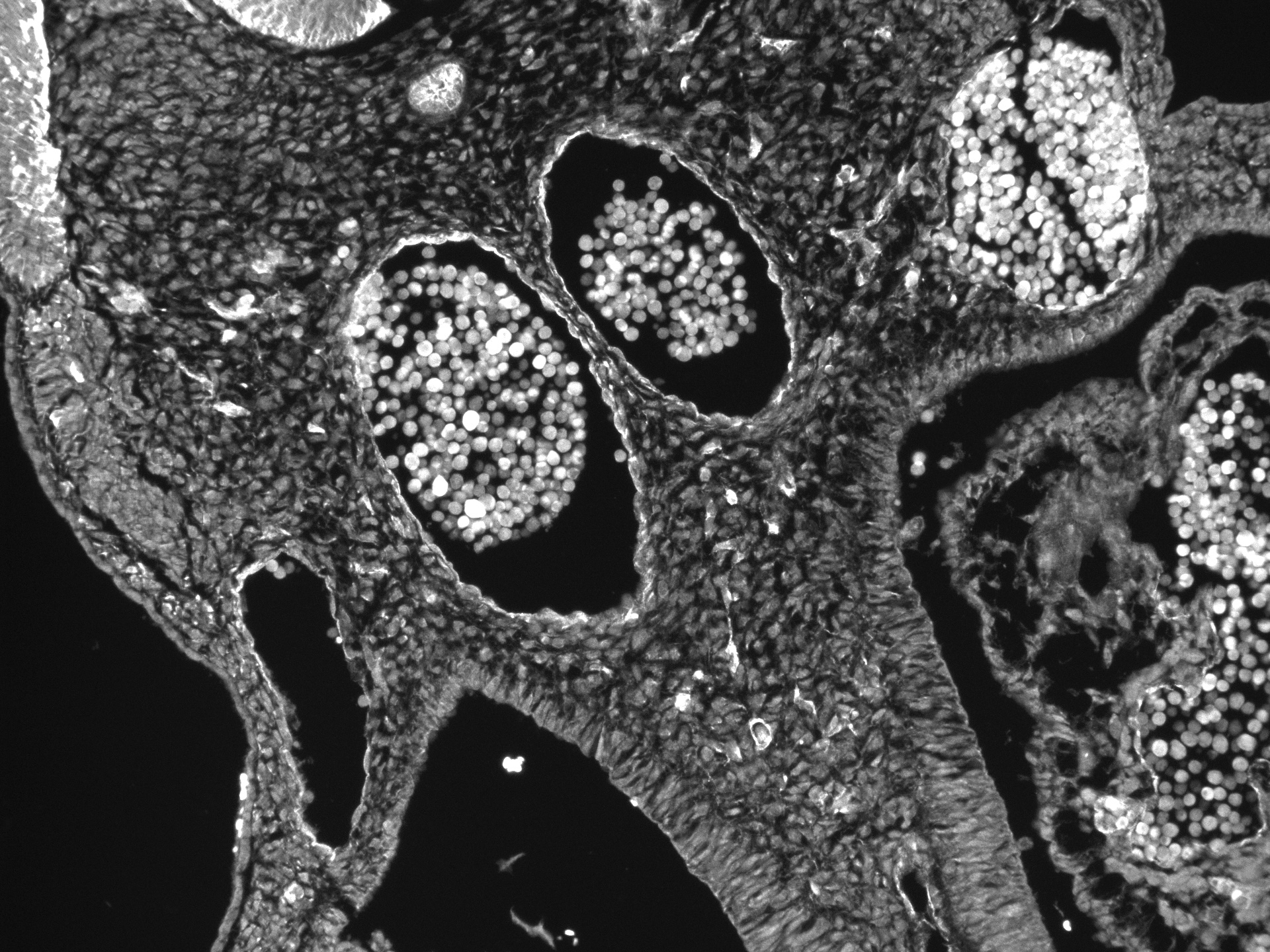

Supplement: Supplementary file 1 — Source Data Fig. 1 [file 44318_2024_45_MOESM1_ESM.zip › Figure1/Figure 1D.jpeg]

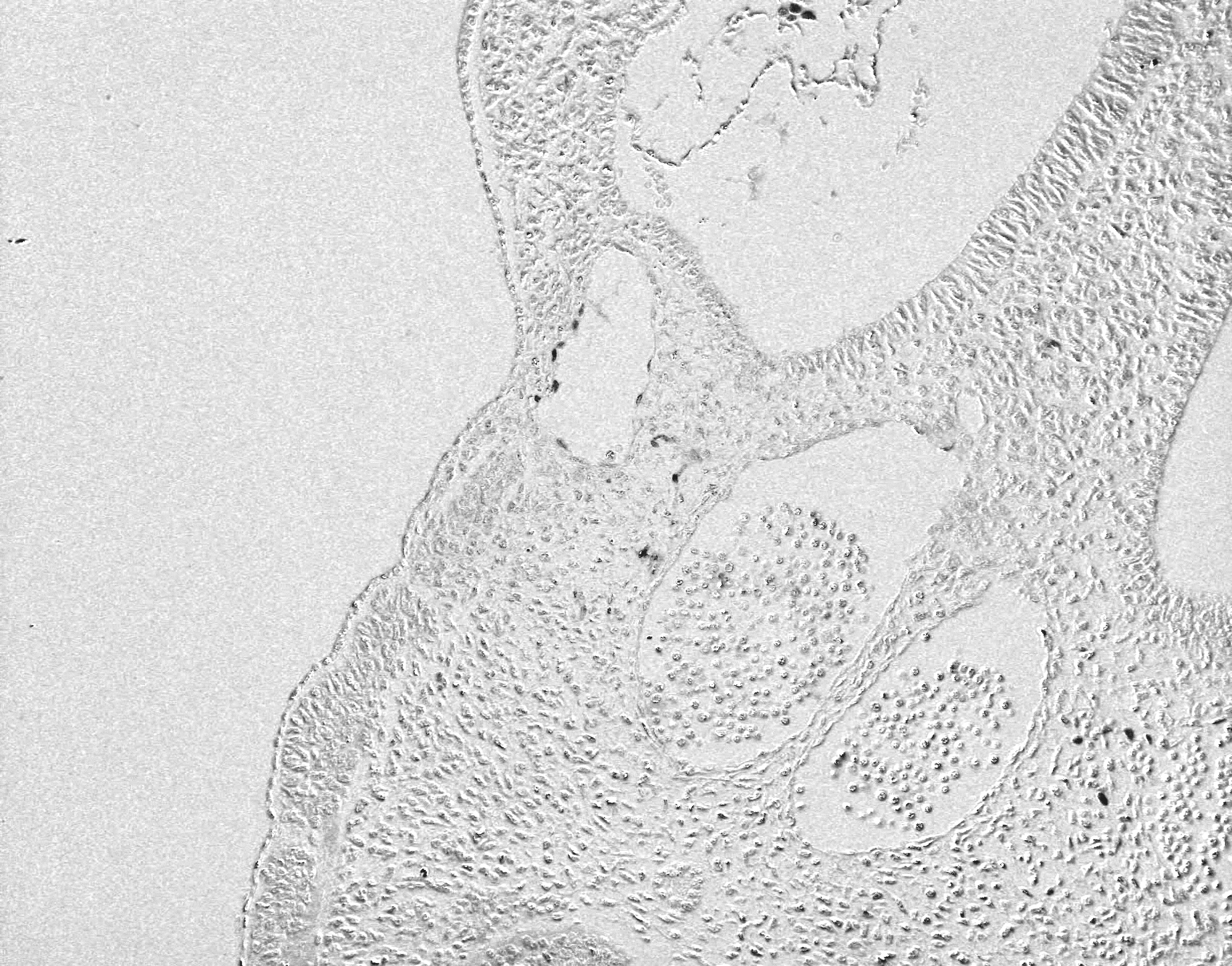

Supplement: Supplementary file 1 — Source Data Fig. 1 [file 44318_2024_45_MOESM1_ESM.zip › Figure1/Figure 1E.jpeg]

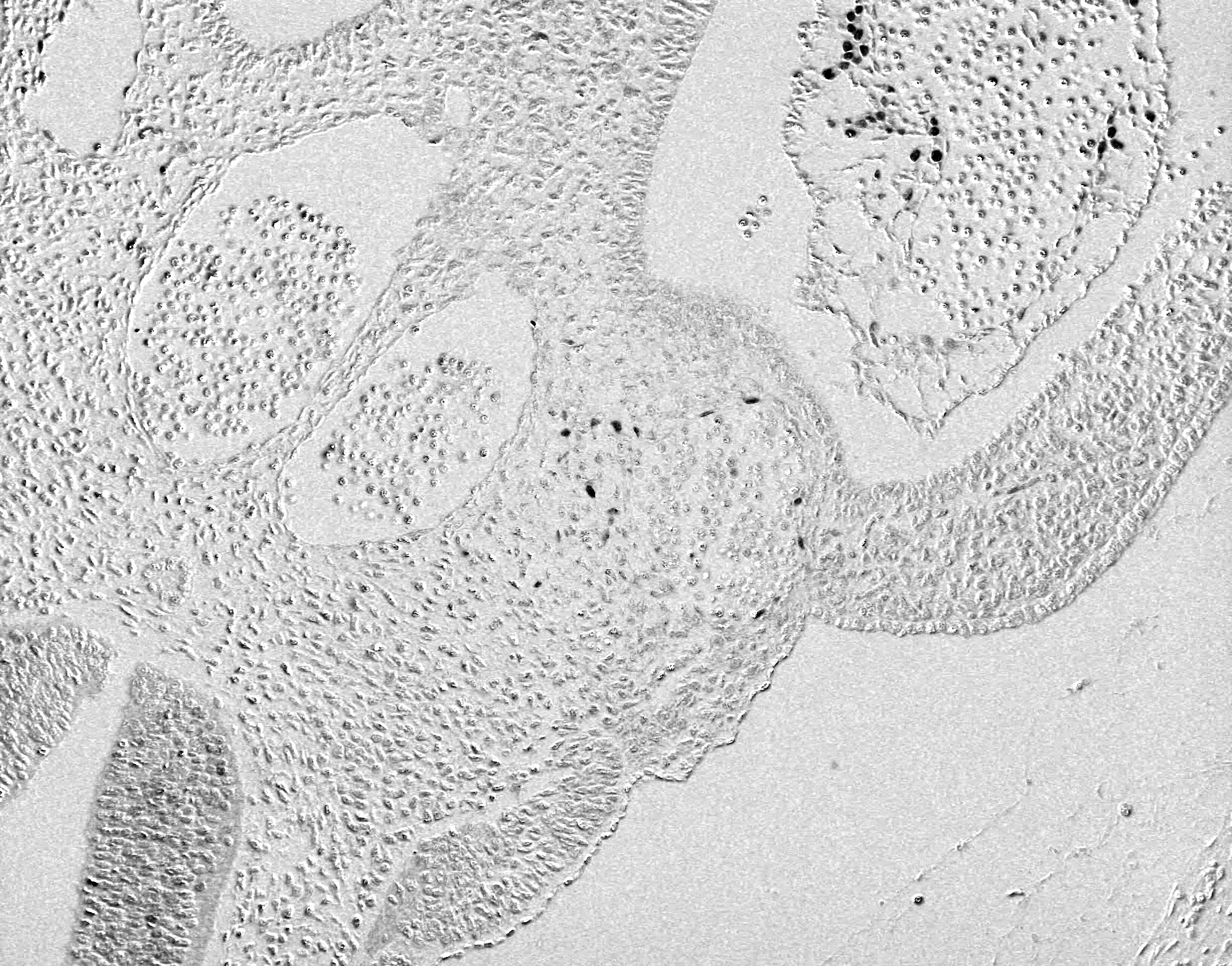

Supplement: Supplementary file 1 — Source Data Fig. 1 [file 44318_2024_45_MOESM1_ESM.zip › Figure1/Figure 1I.jpeg]

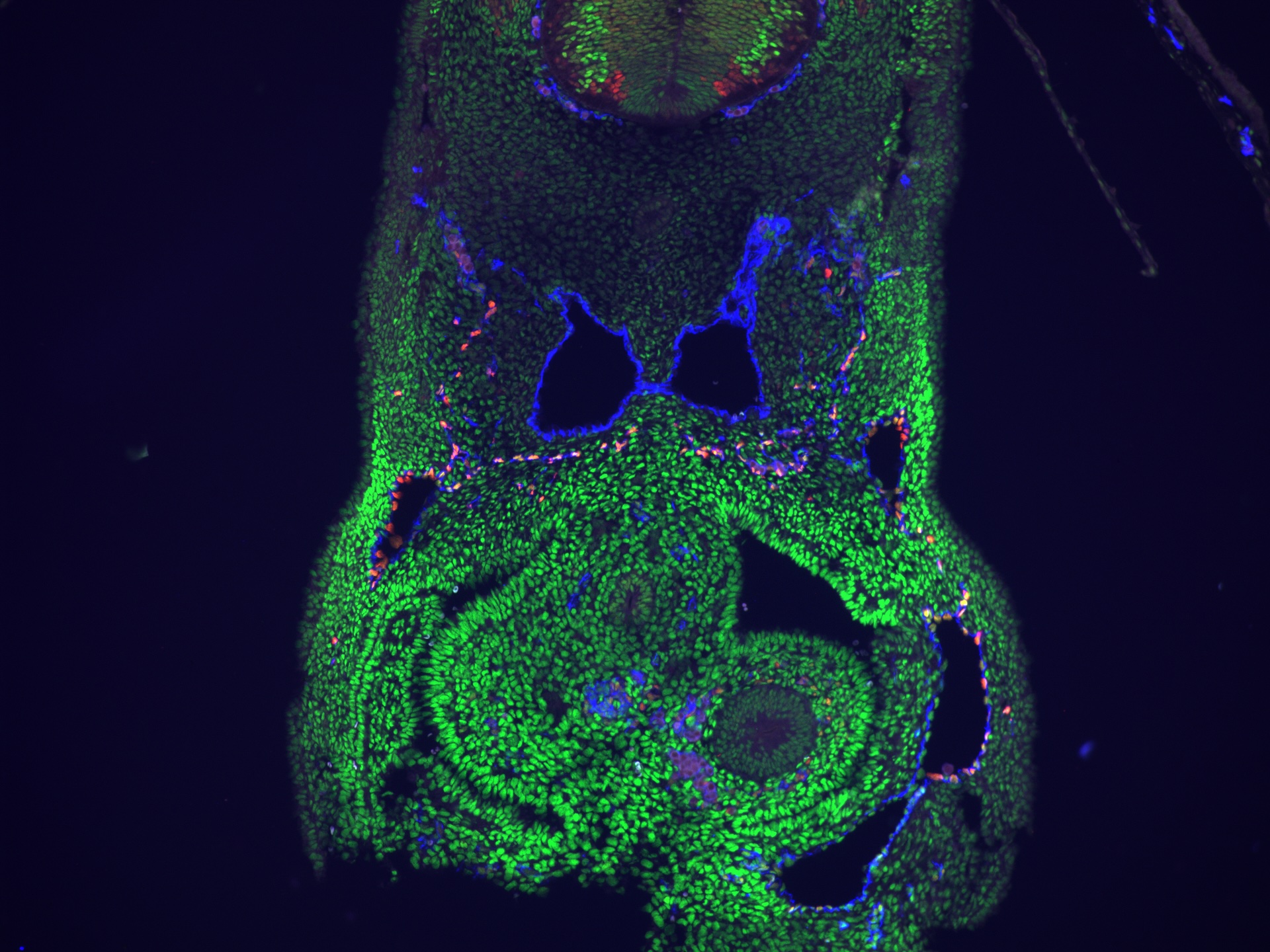

Supplement: Supplementary file 1 — Source Data Fig. 1 [file 44318_2024_45_MOESM1_ESM.zip › Figure1/Figure 1J.jpeg]

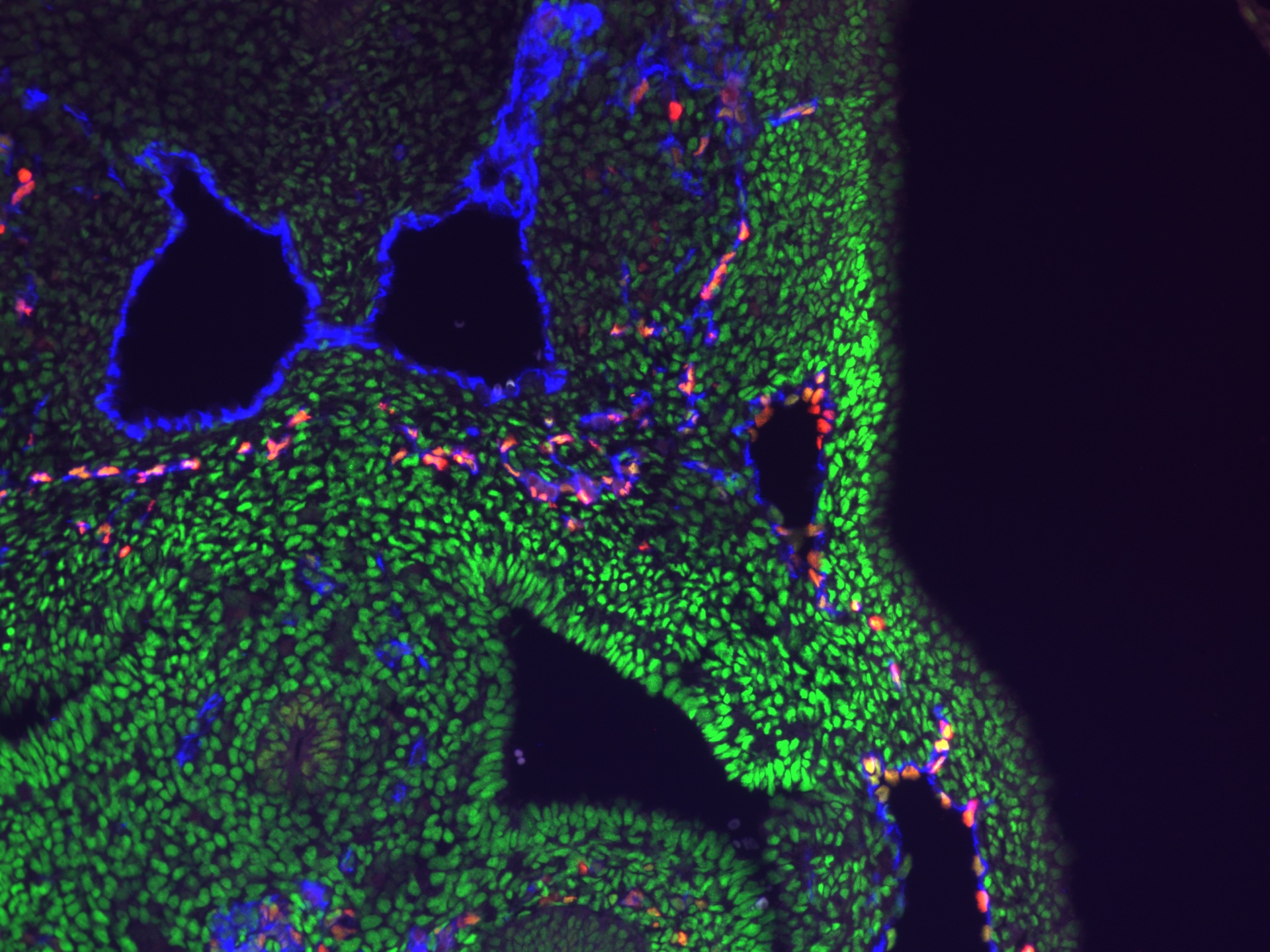

Supplement: Supplementary file 1 — Source Data Fig. 1 [file 44318_2024_45_MOESM1_ESM.zip › Figure1/Figure 1K.jpeg]

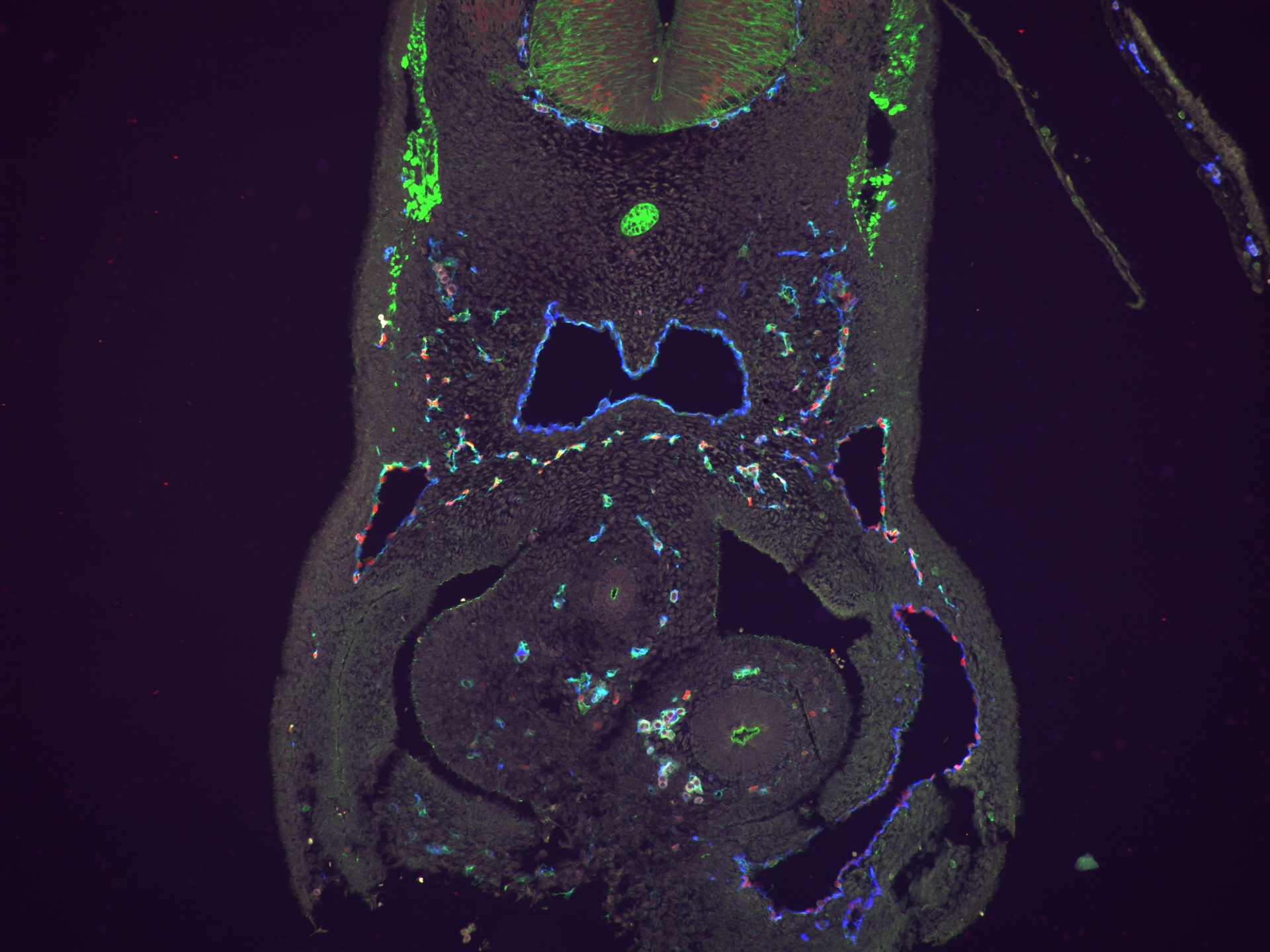

Supplement: Supplementary file 1 — Source Data Fig. 1 [file 44318_2024_45_MOESM1_ESM.zip › Figure1/Figure 1L.jpeg]

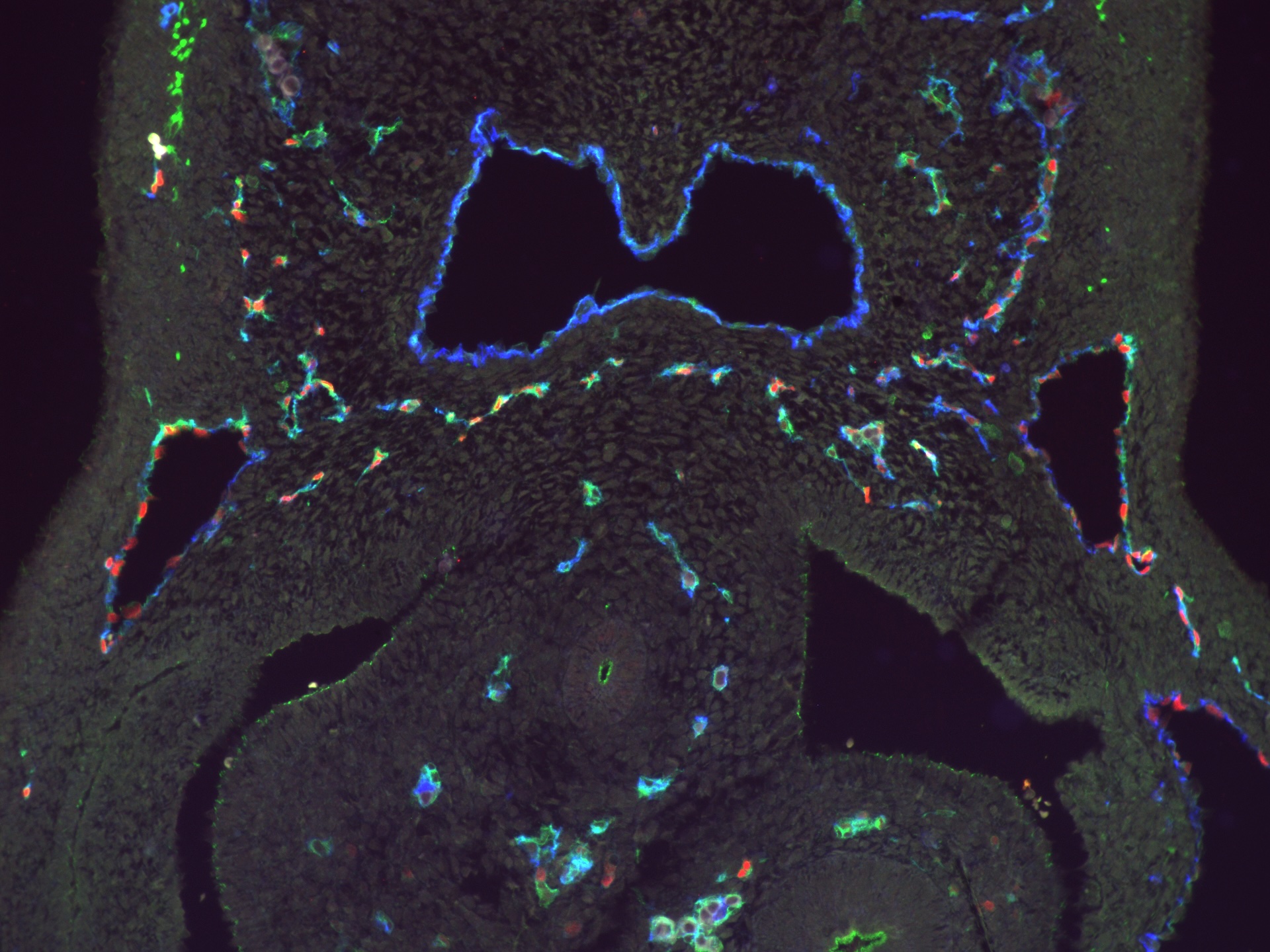

Supplement: Supplementary file 1 — Source Data Fig. 1 [file 44318_2024_45_MOESM1_ESM.zip › Figure1/Figure 1M.jpeg]

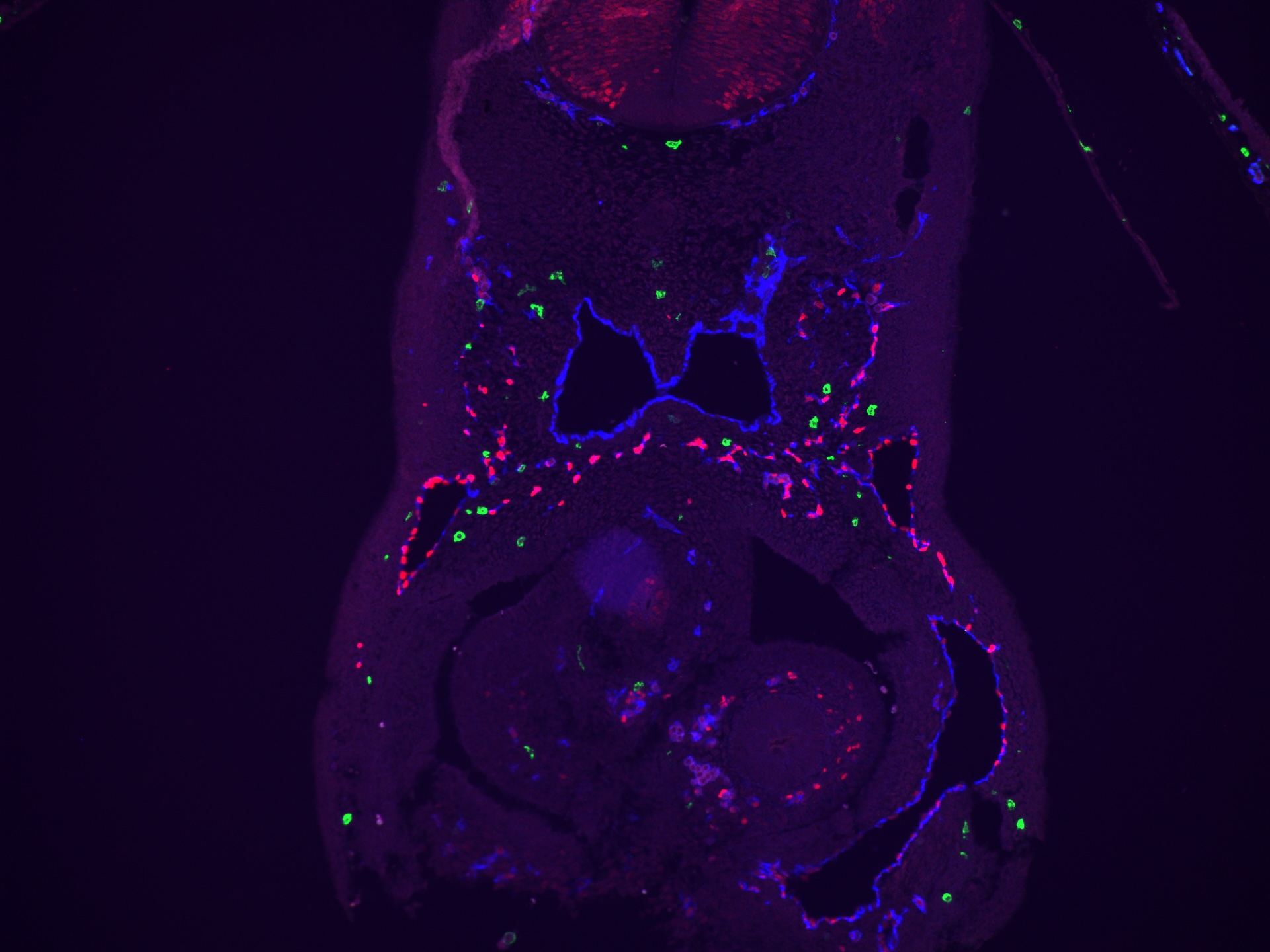

Supplement: Supplementary file 1 — Source Data Fig. 1 [file 44318_2024_45_MOESM1_ESM.zip › Figure1/Figure 1N.jpeg]

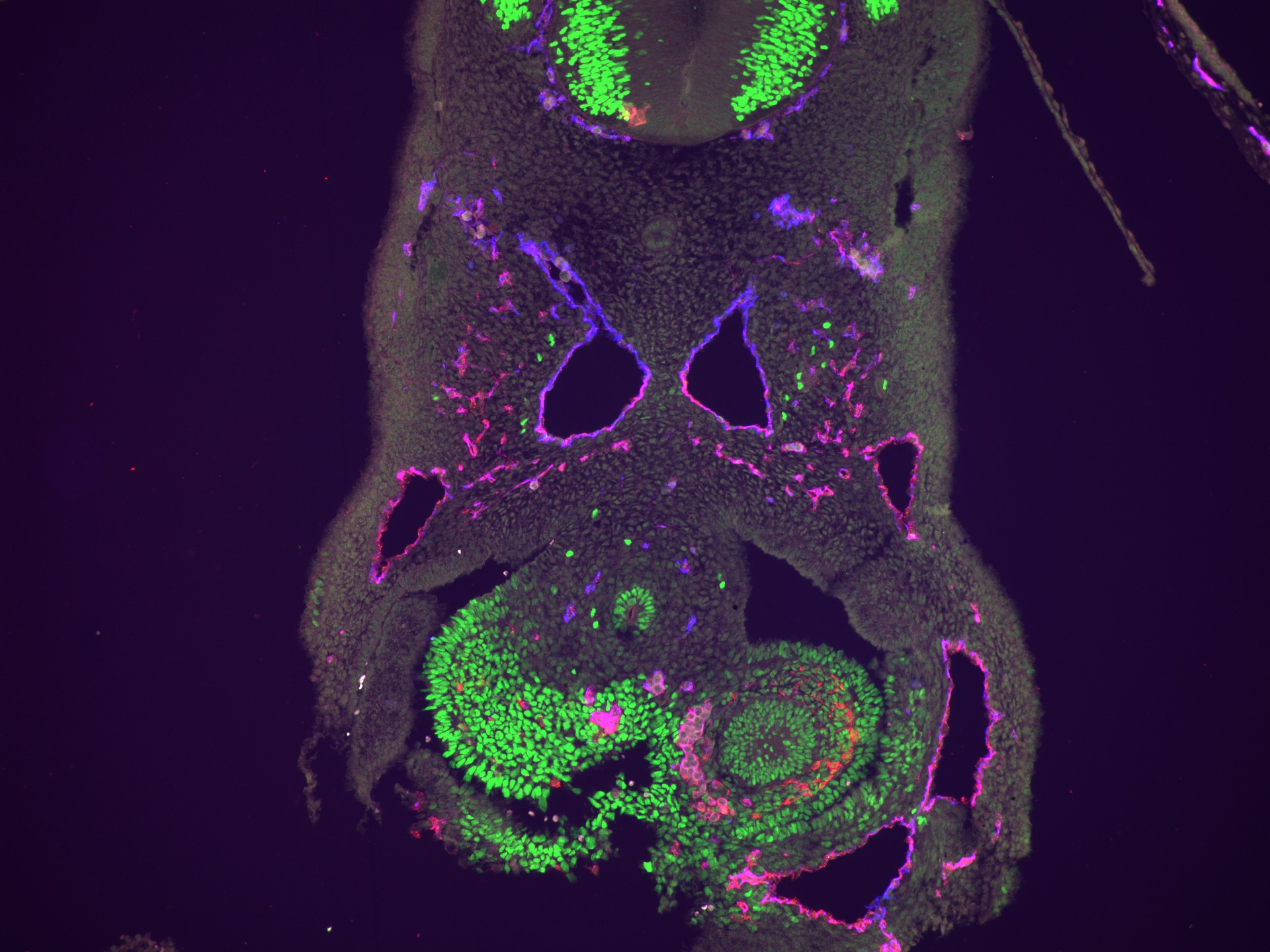

Supplement: Supplementary file 1 — Source Data Fig. 1 [file 44318_2024_45_MOESM1_ESM.zip › Figure1/Figure 1P.jpeg]

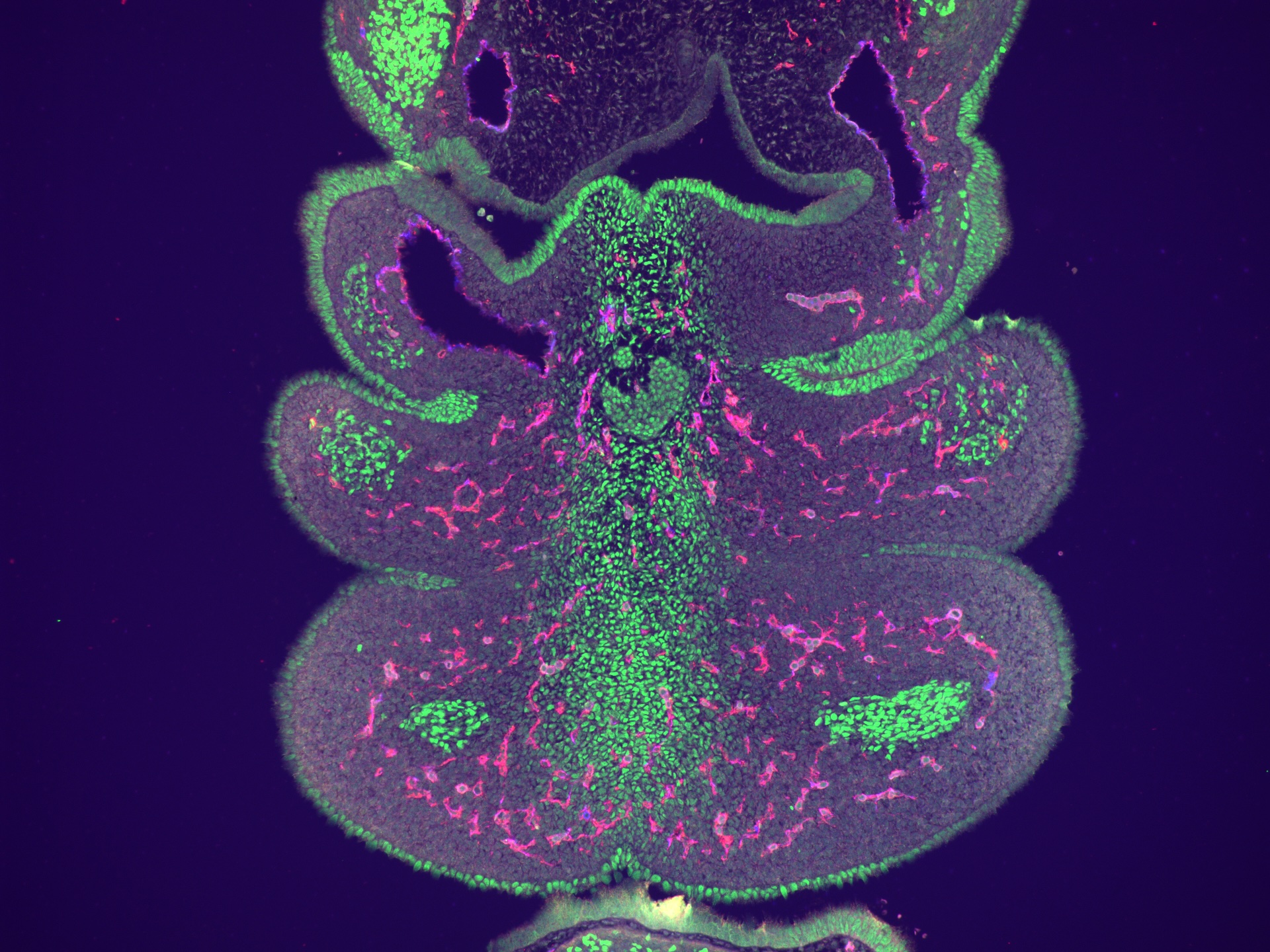

Supplement: Supplementary file 1 — Source Data Fig. 1 [file 44318_2024_45_MOESM1_ESM.zip › Figure1/Figure 1R.jpeg]

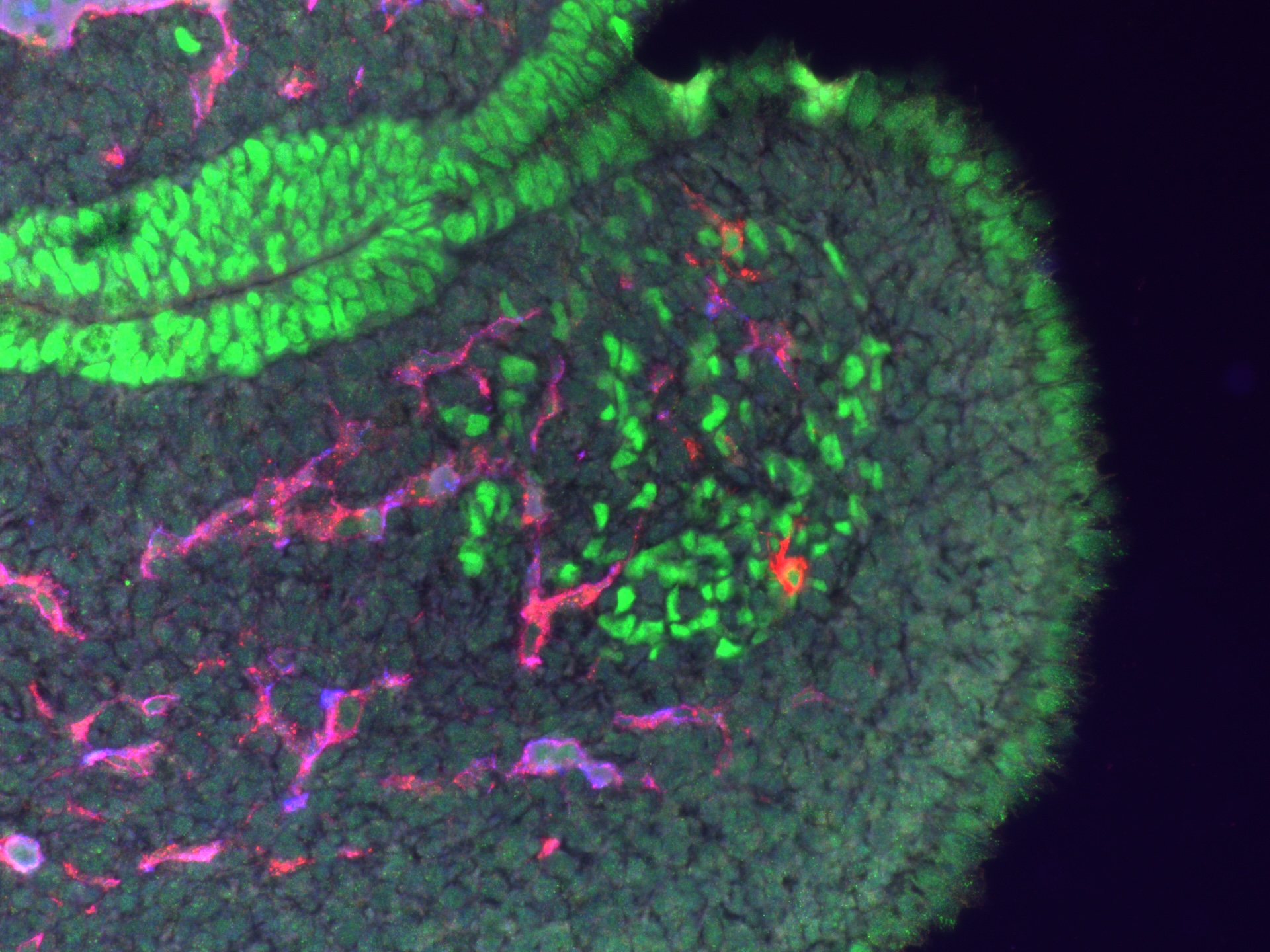

Supplement: Supplementary file 1 — Source Data Fig. 1 [file 44318_2024_45_MOESM1_ESM.zip › Figure1/Figure 1S.jpeg]

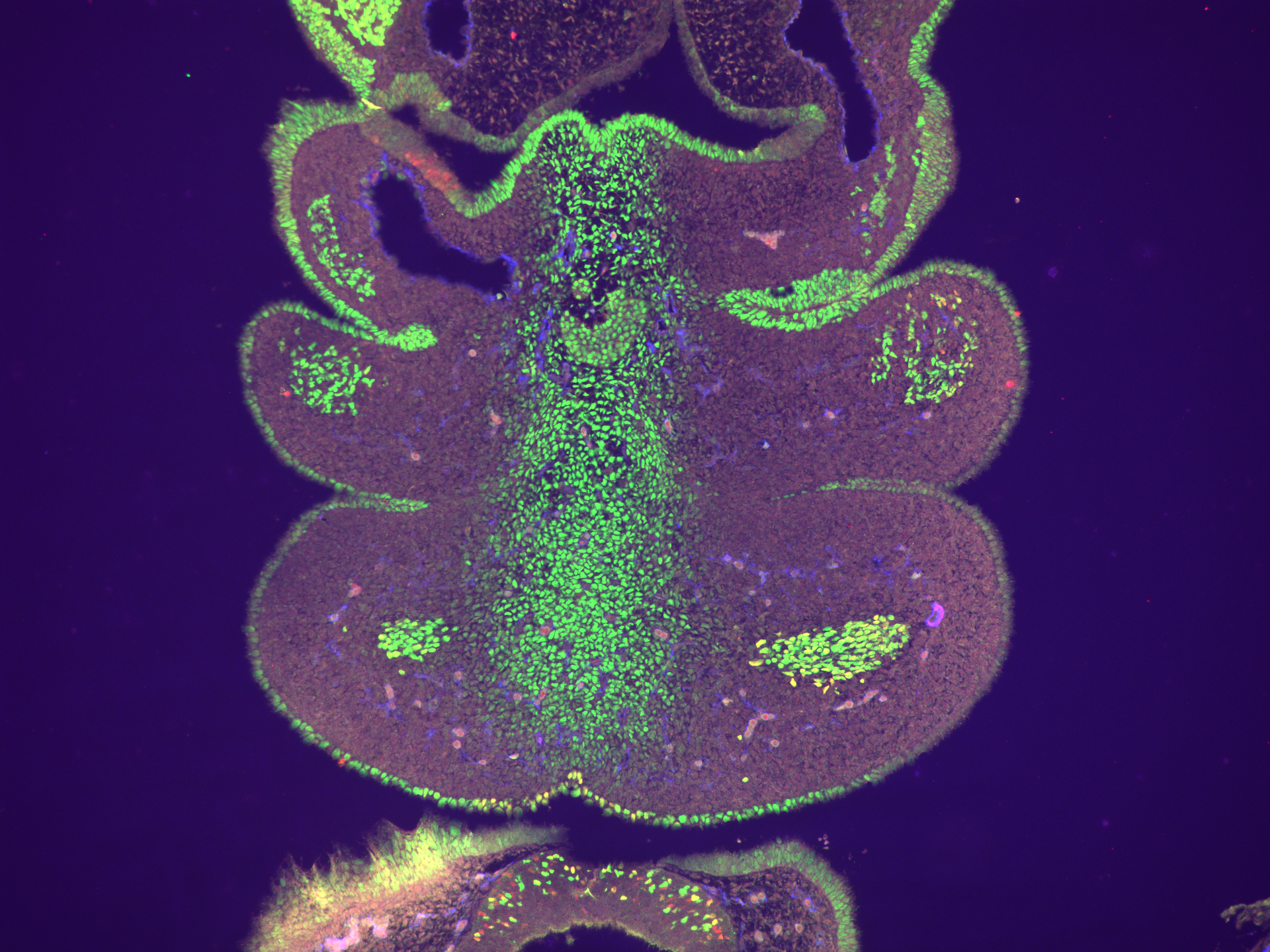

Supplement: Supplementary file 1 — Source Data Fig. 1 [file 44318_2024_45_MOESM1_ESM.zip › Figure1/Figure 1T.jpeg]

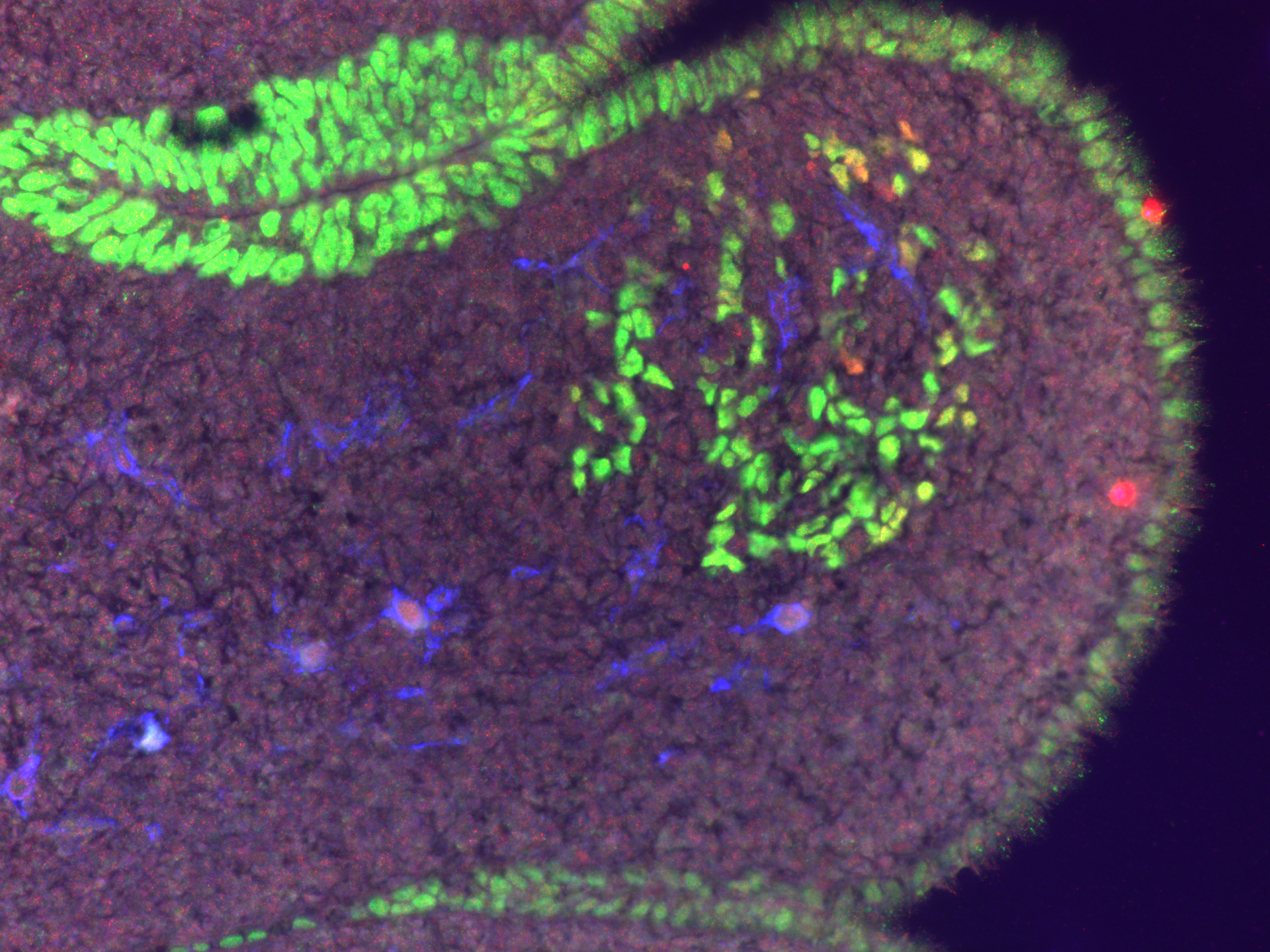

Supplement: Supplementary file 1 — Source Data Fig. 1 [file 44318_2024_45_MOESM1_ESM.zip › Figure1/Figure 1U.jpeg]

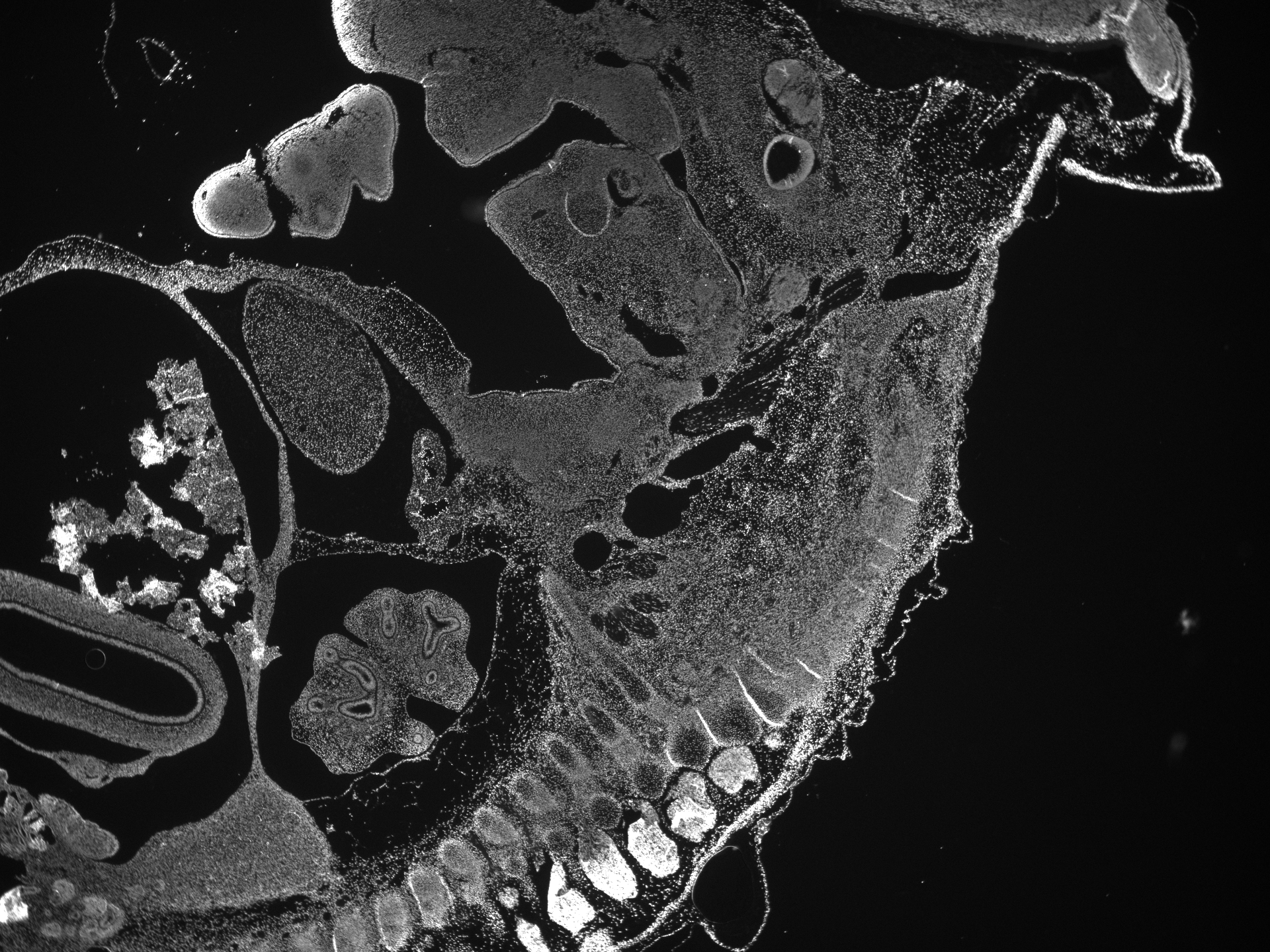

Supplement: Supplementary file 2 — Source Data Fig. 2 [file 44318_2024_45_MOESM2_ESM.zip › Figure2/Figure-2A.jpeg]

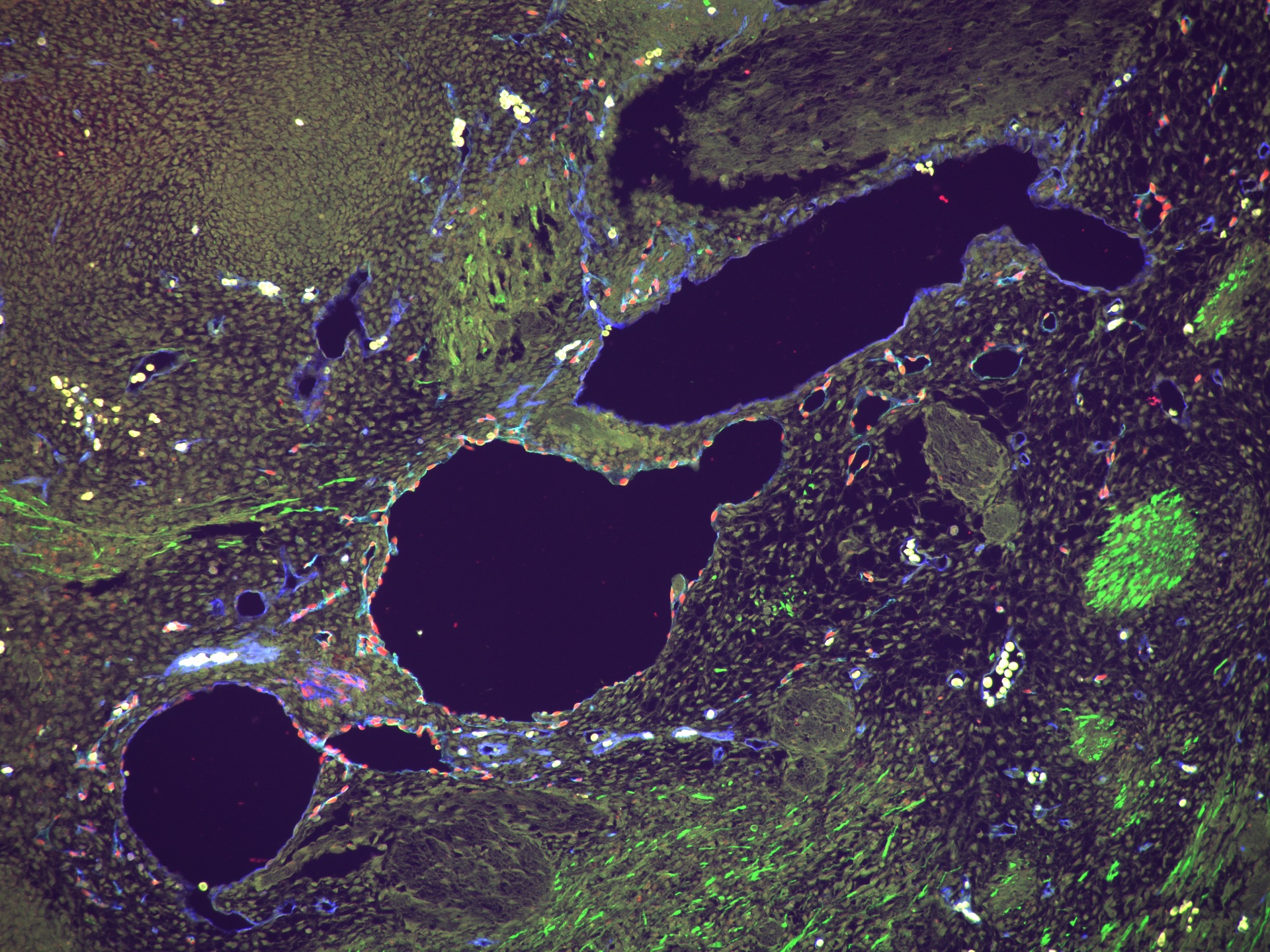

Supplement: Supplementary file 2 — Source Data Fig. 2 [file 44318_2024_45_MOESM2_ESM.zip › Figure2/Figure-2B.jpeg]

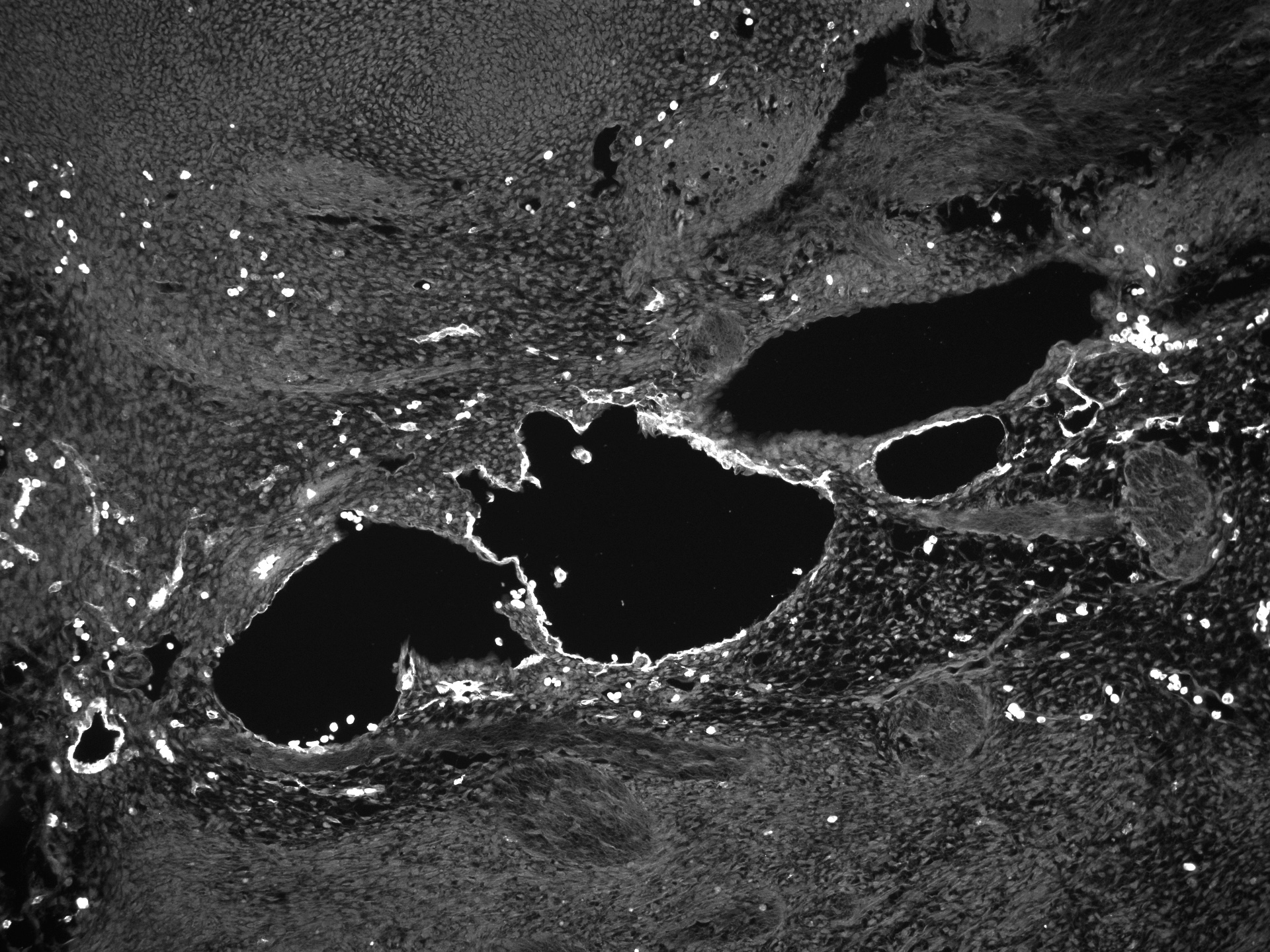

Supplement: Supplementary file 2 — Source Data Fig. 2 [file 44318_2024_45_MOESM2_ESM.zip › Figure2/Figure-2C.jpeg]

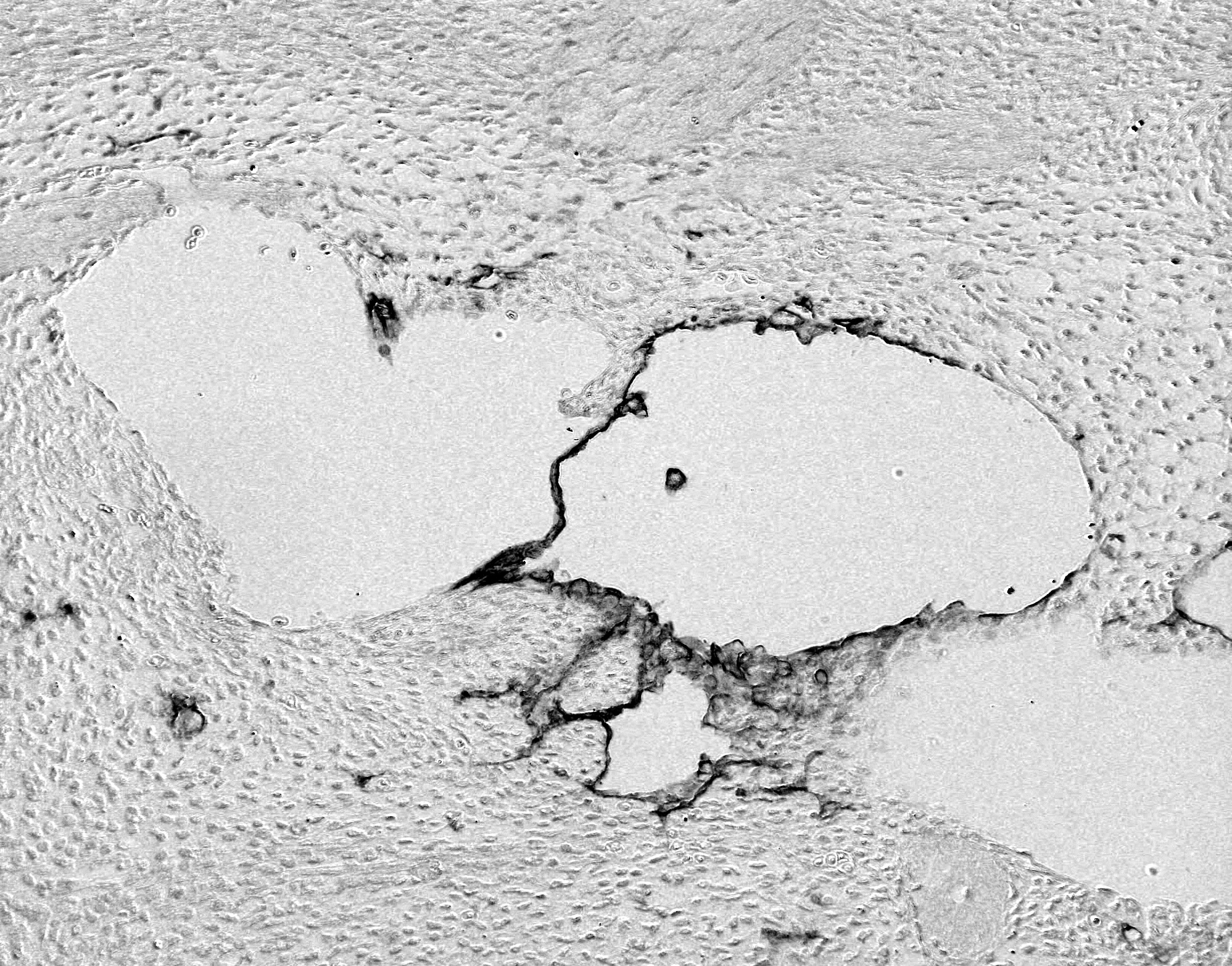

Supplement: Supplementary file 2 — Source Data Fig. 2 [file 44318_2024_45_MOESM2_ESM.zip › Figure2/Figure-2D.jpeg]

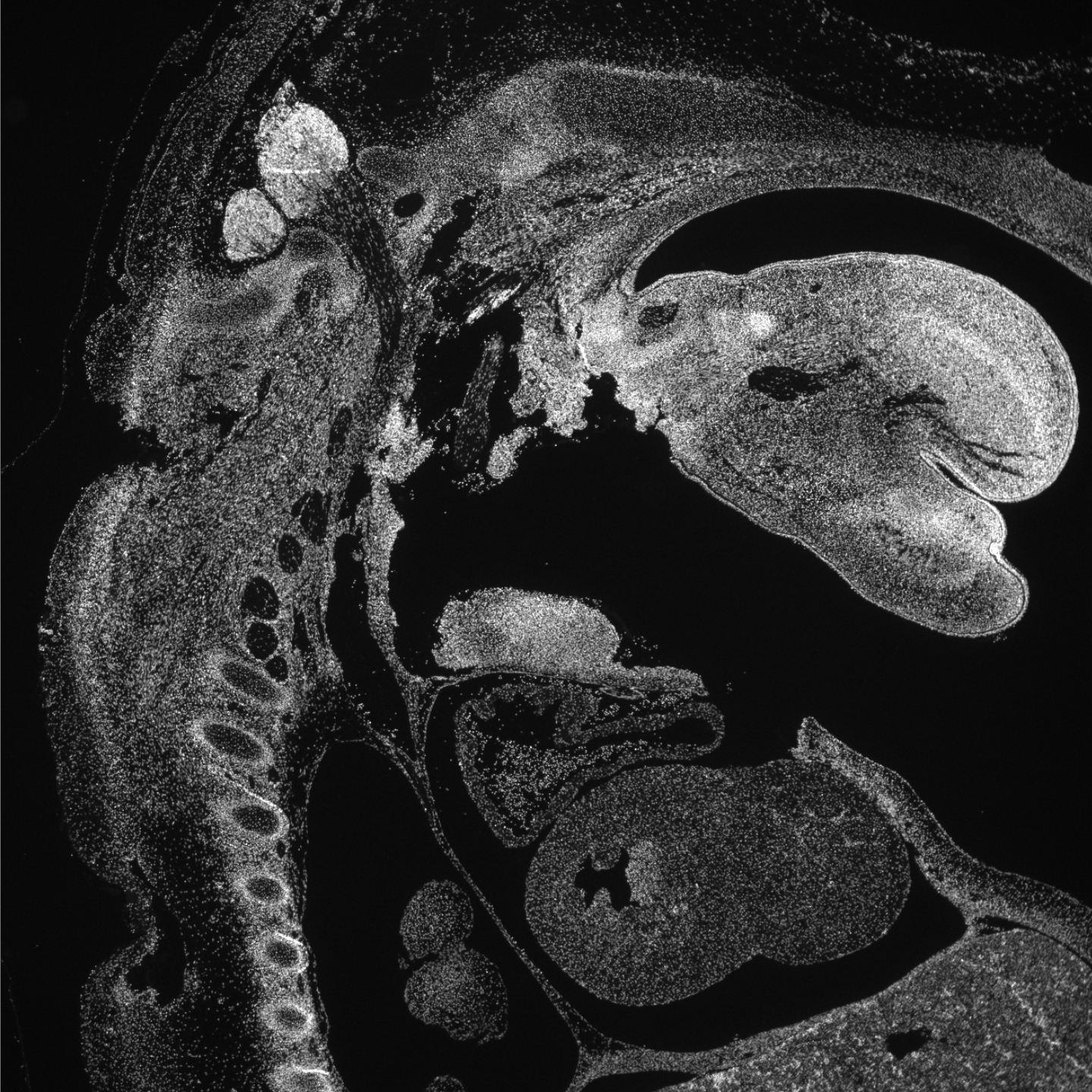

Supplement: Supplementary file 2 — Source Data Fig. 2 [file 44318_2024_45_MOESM2_ESM.zip › Figure2/Figure-2E.jpeg]

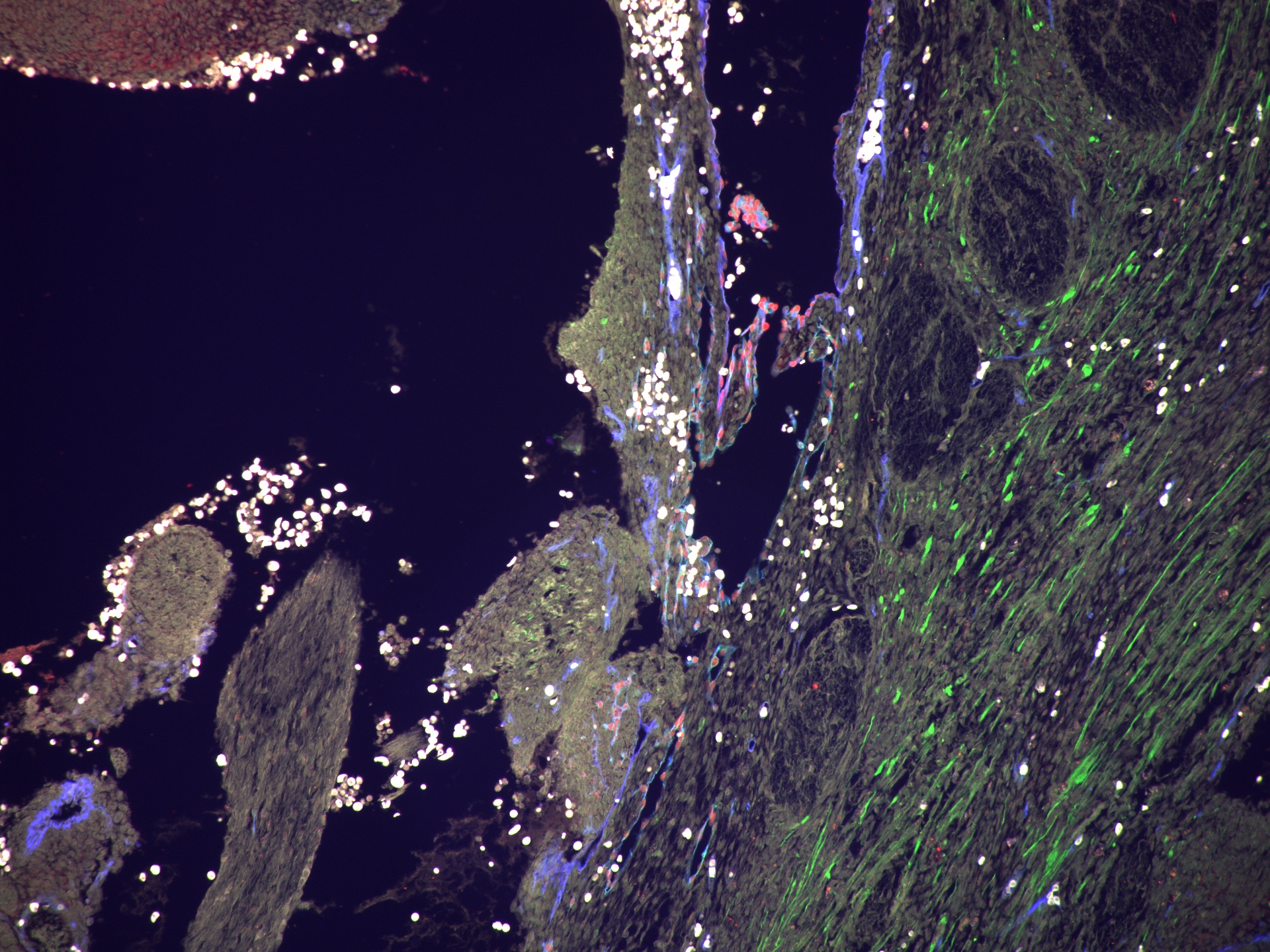

Supplement: Supplementary file 2 — Source Data Fig. 2 [file 44318_2024_45_MOESM2_ESM.zip › Figure2/Figure-2F.jpeg]

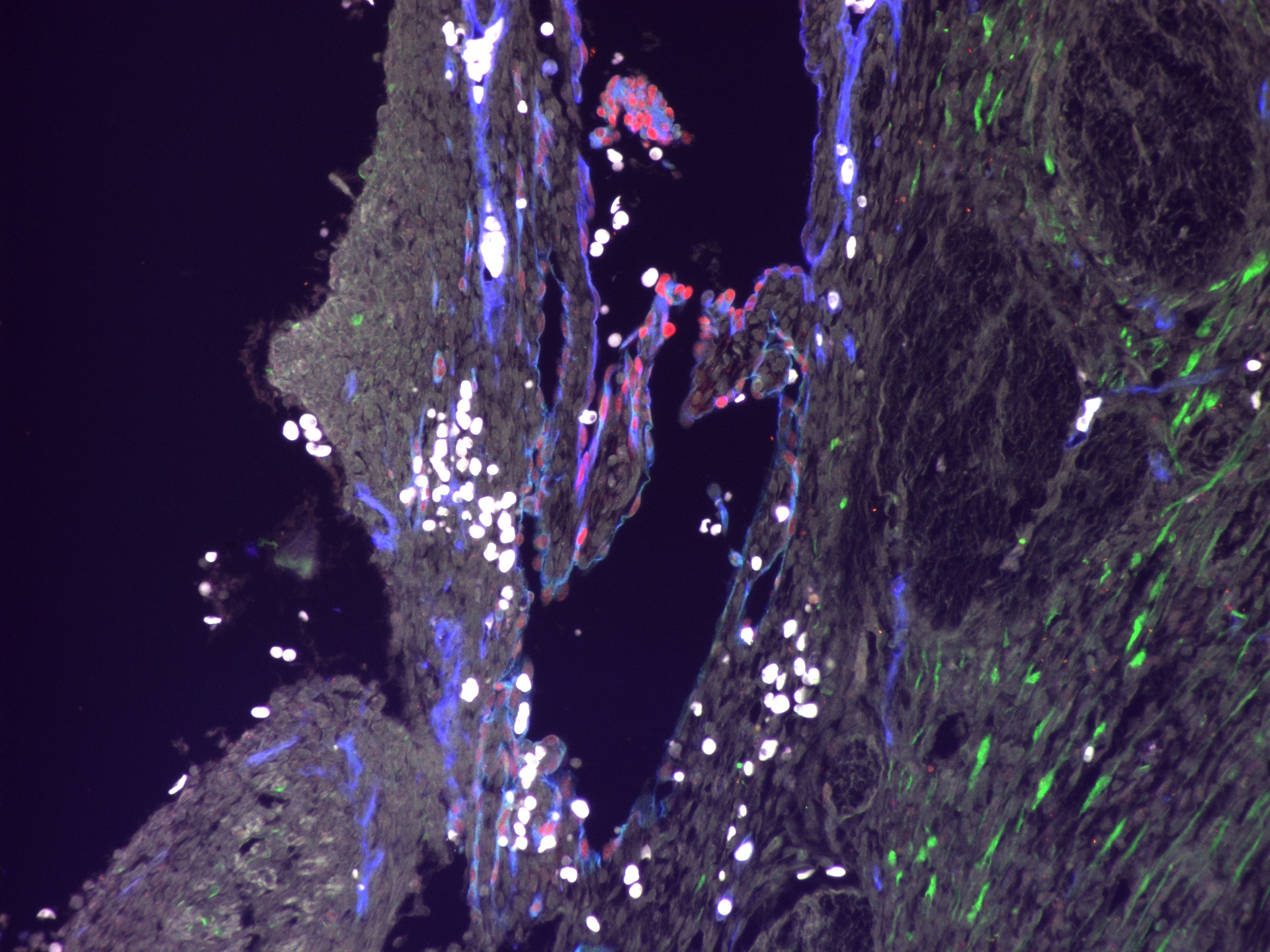

Supplement: Supplementary file 2 — Source Data Fig. 2 [file 44318_2024_45_MOESM2_ESM.zip › Figure2/Figure-2G.jpeg]

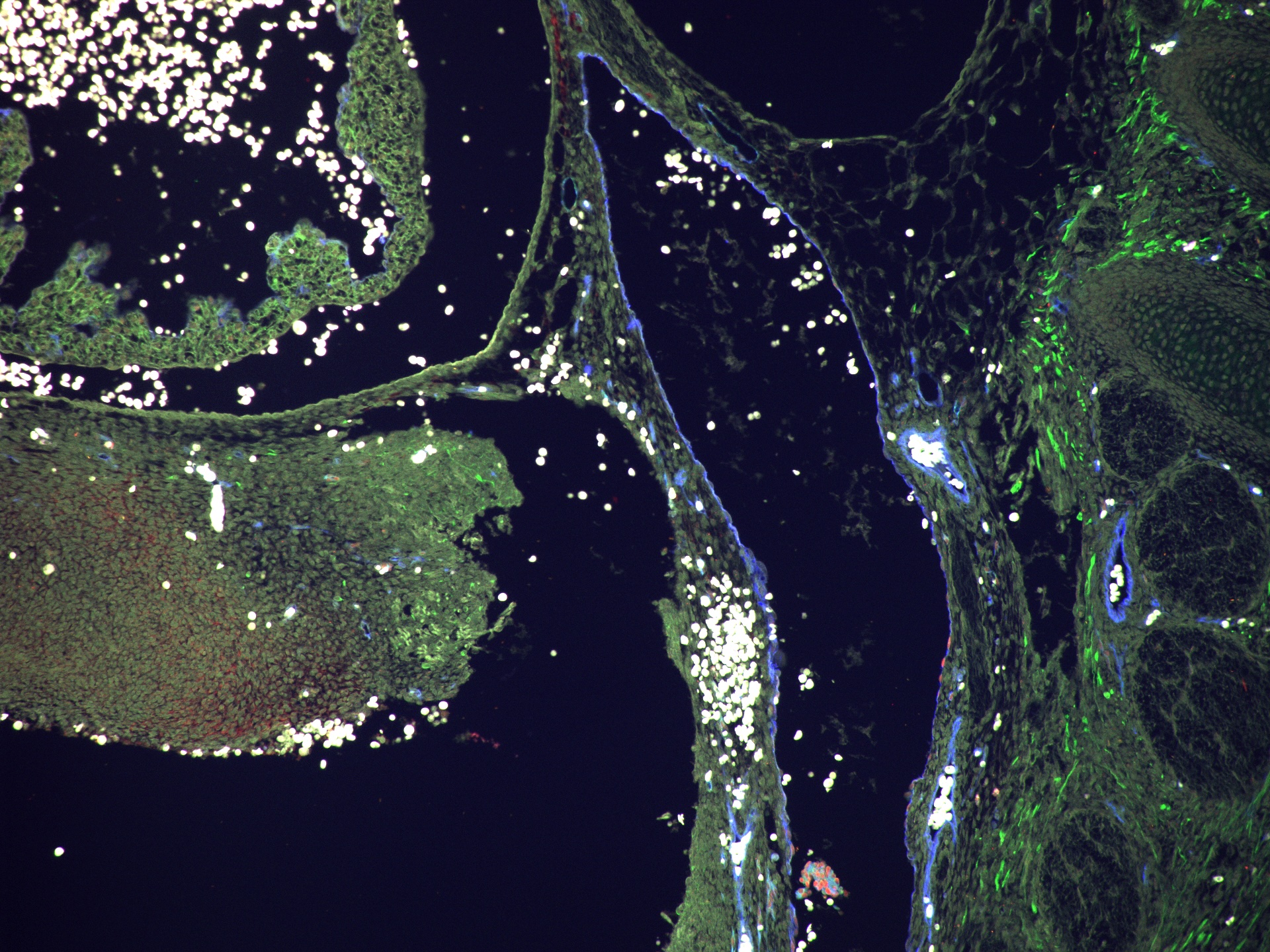

Supplement: Supplementary file 2 — Source Data Fig. 2 [file 44318_2024_45_MOESM2_ESM.zip › Figure2/Figure-2H.jpeg]

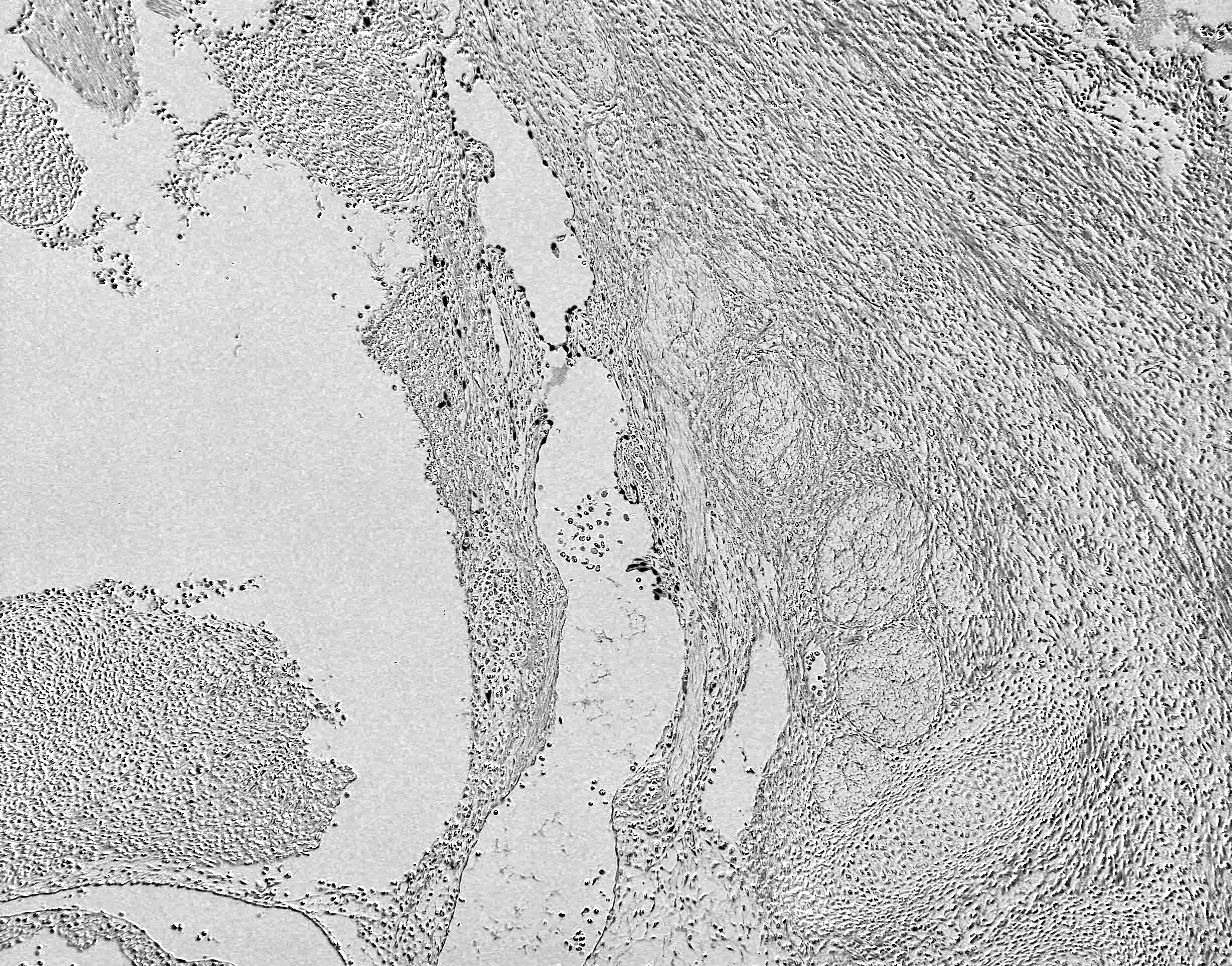

Supplement: Supplementary file 2 — Source Data Fig. 2 [file 44318_2024_45_MOESM2_ESM.zip › Figure2/Figure-2J.jpeg]

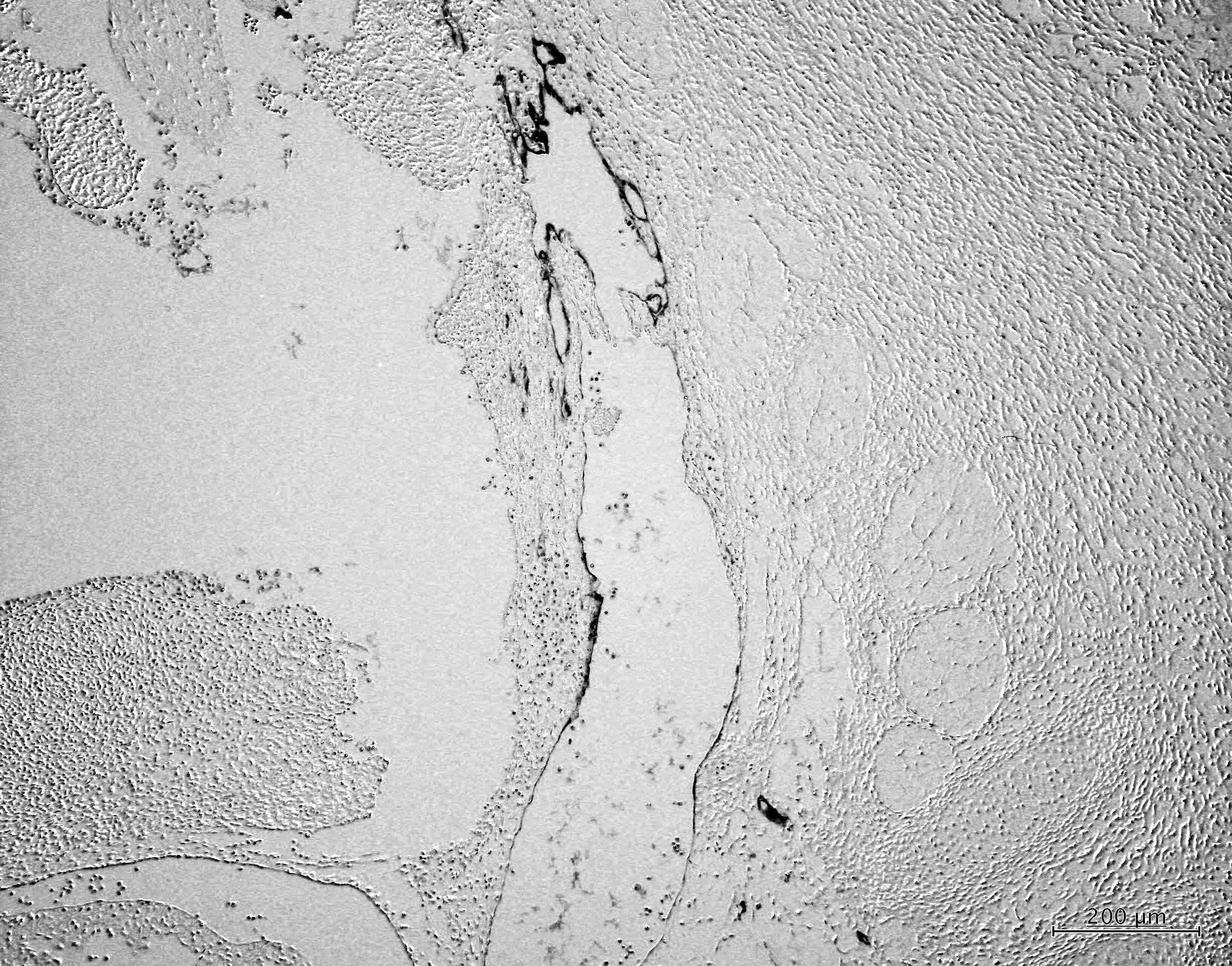

Supplement: Supplementary file 2 — Source Data Fig. 2 [file 44318_2024_45_MOESM2_ESM.zip › Figure2/Figure-2K.jpeg]

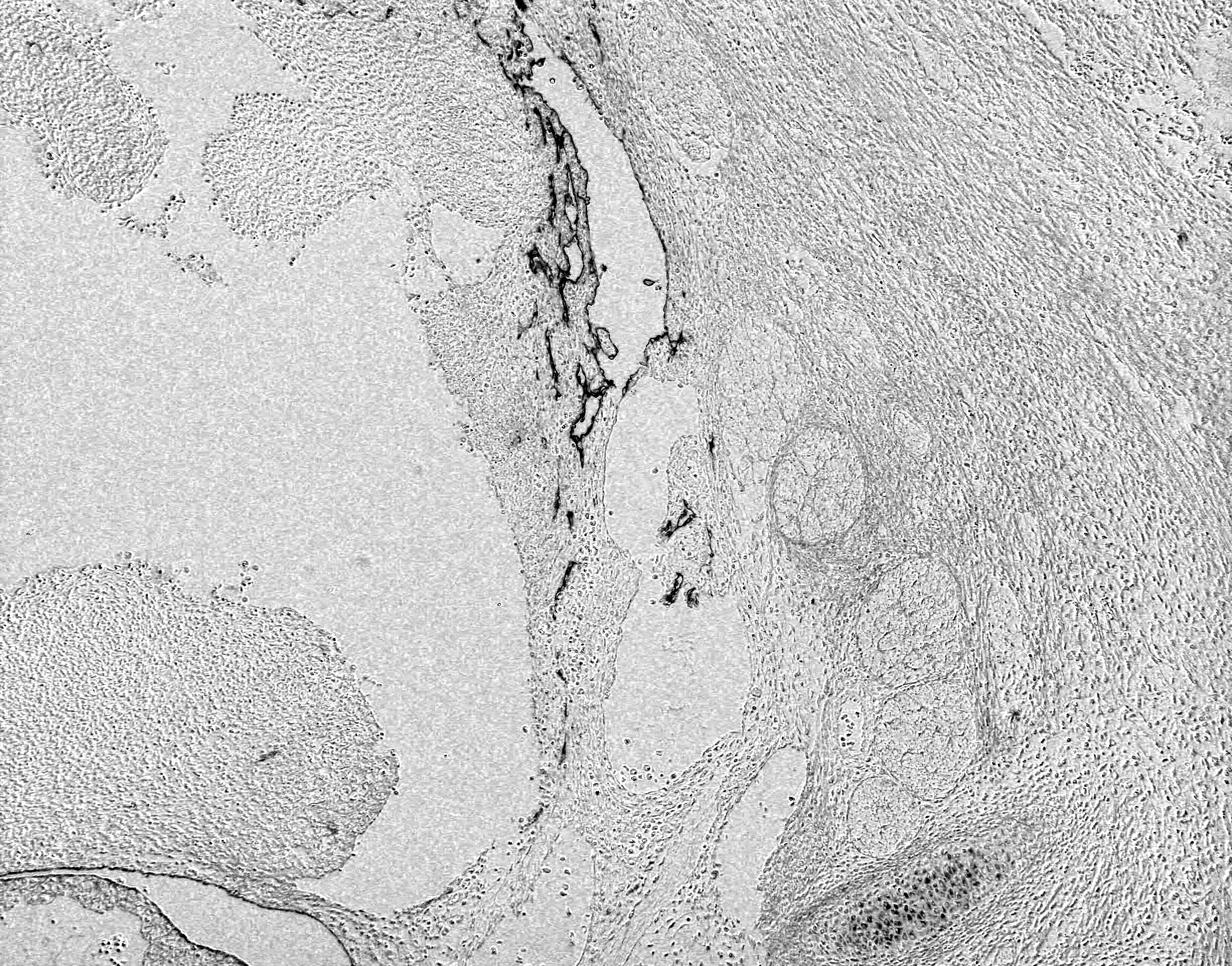

Supplement: Supplementary file 2 — Source Data Fig. 2 [file 44318_2024_45_MOESM2_ESM.zip › Figure2/Figure-2L.jpeg]

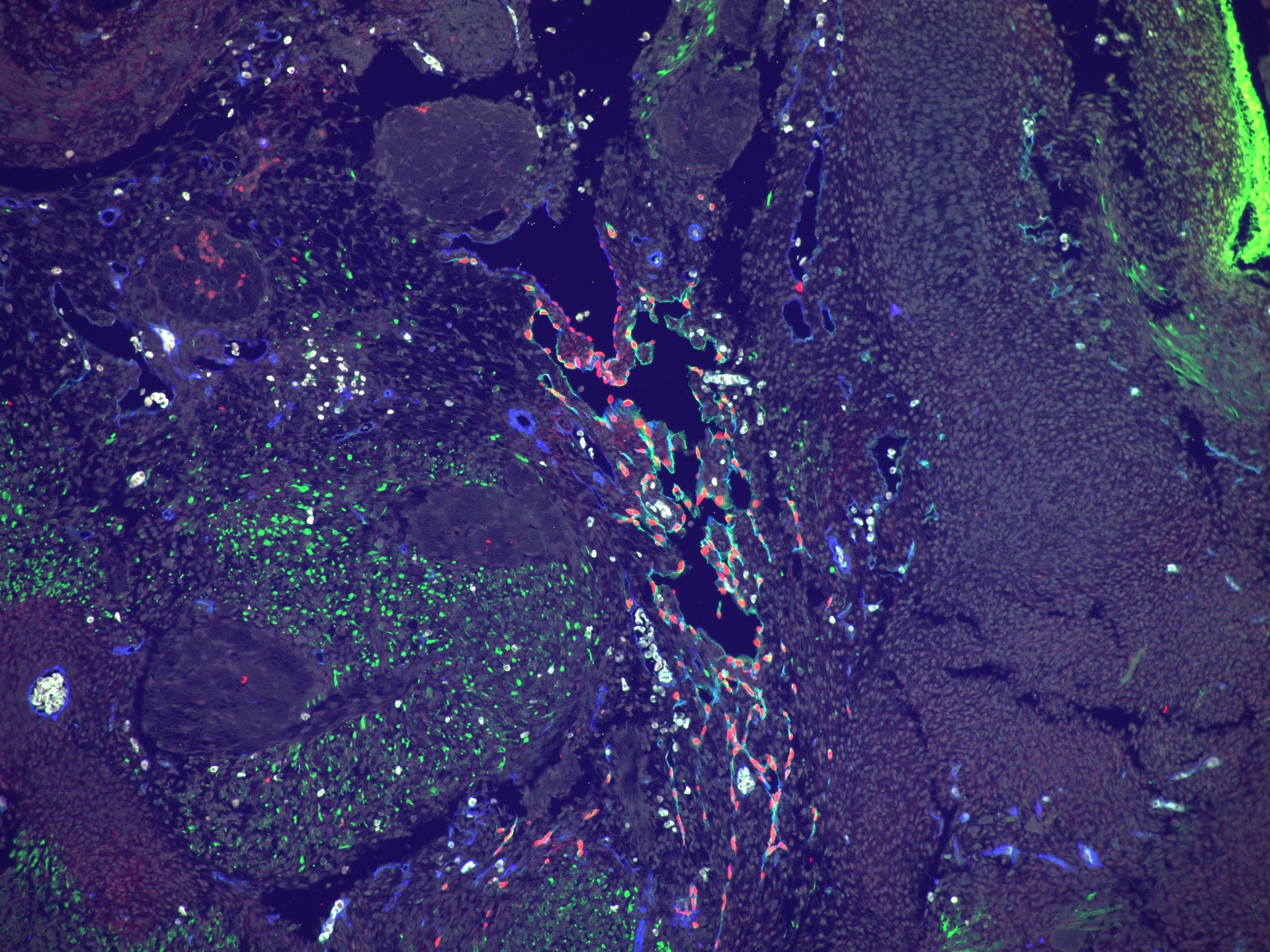

Supplement: Supplementary file 2 — Source Data Fig. 2 [file 44318_2024_45_MOESM2_ESM.zip › Figure2/Figure-2O.jpeg]

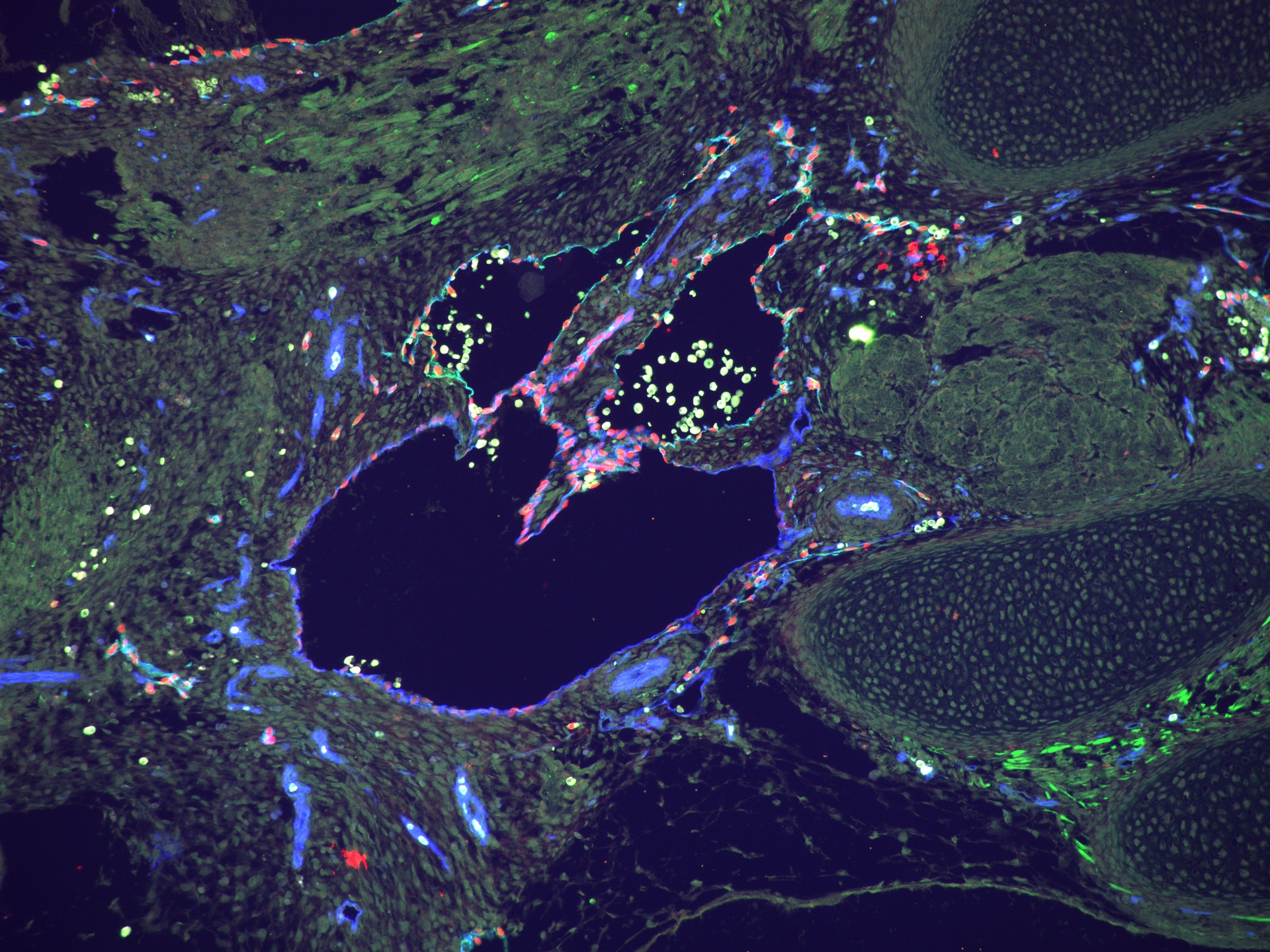

Supplement: Supplementary file 2 — Source Data Fig. 2 [file 44318_2024_45_MOESM2_ESM.zip › Figure2/Figure-2Q.jpeg]

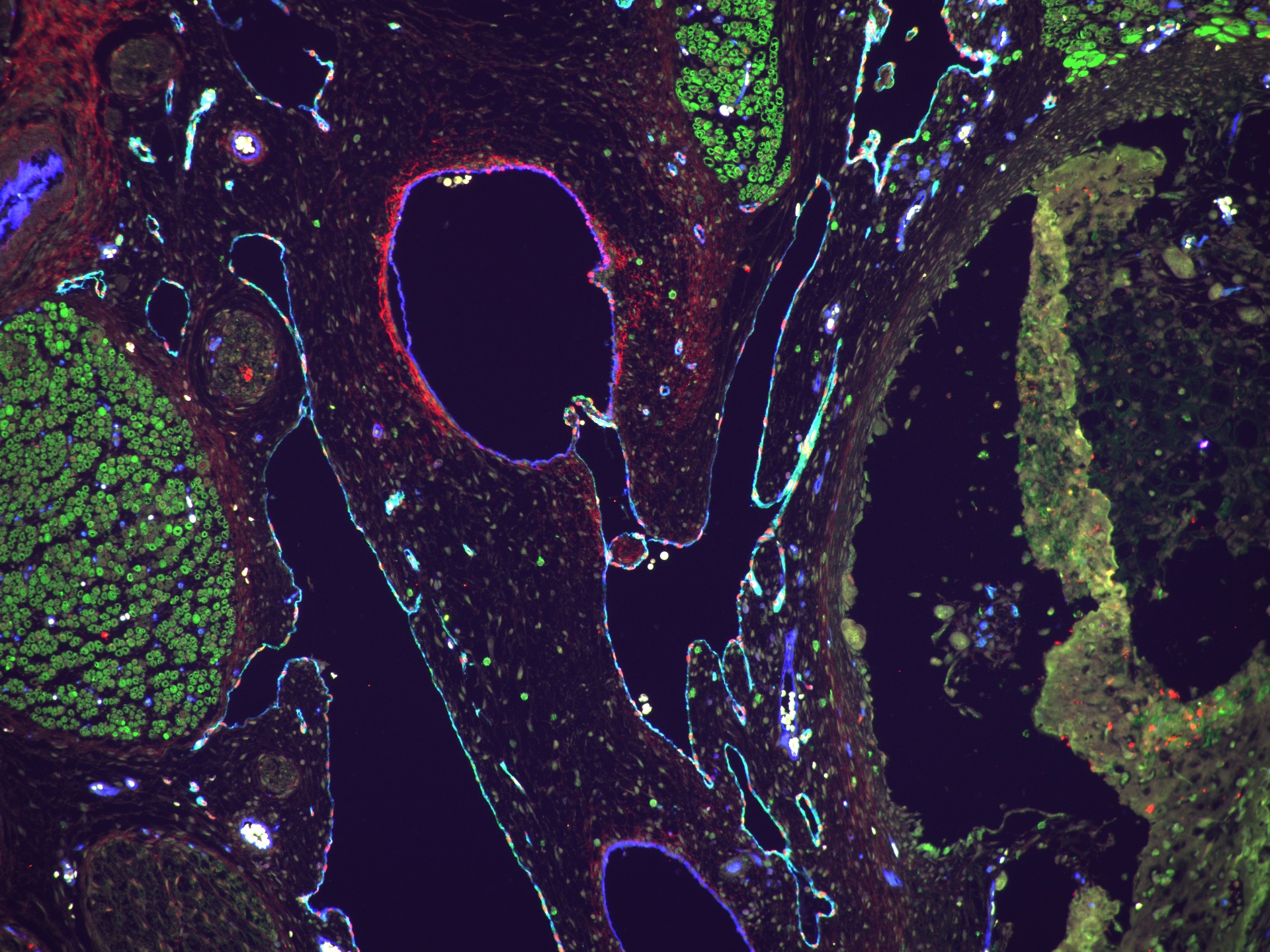

Supplement: Supplementary file 2 — Source Data Fig. 2 [file 44318_2024_45_MOESM2_ESM.zip › Figure2/Figure-2R.jpeg]

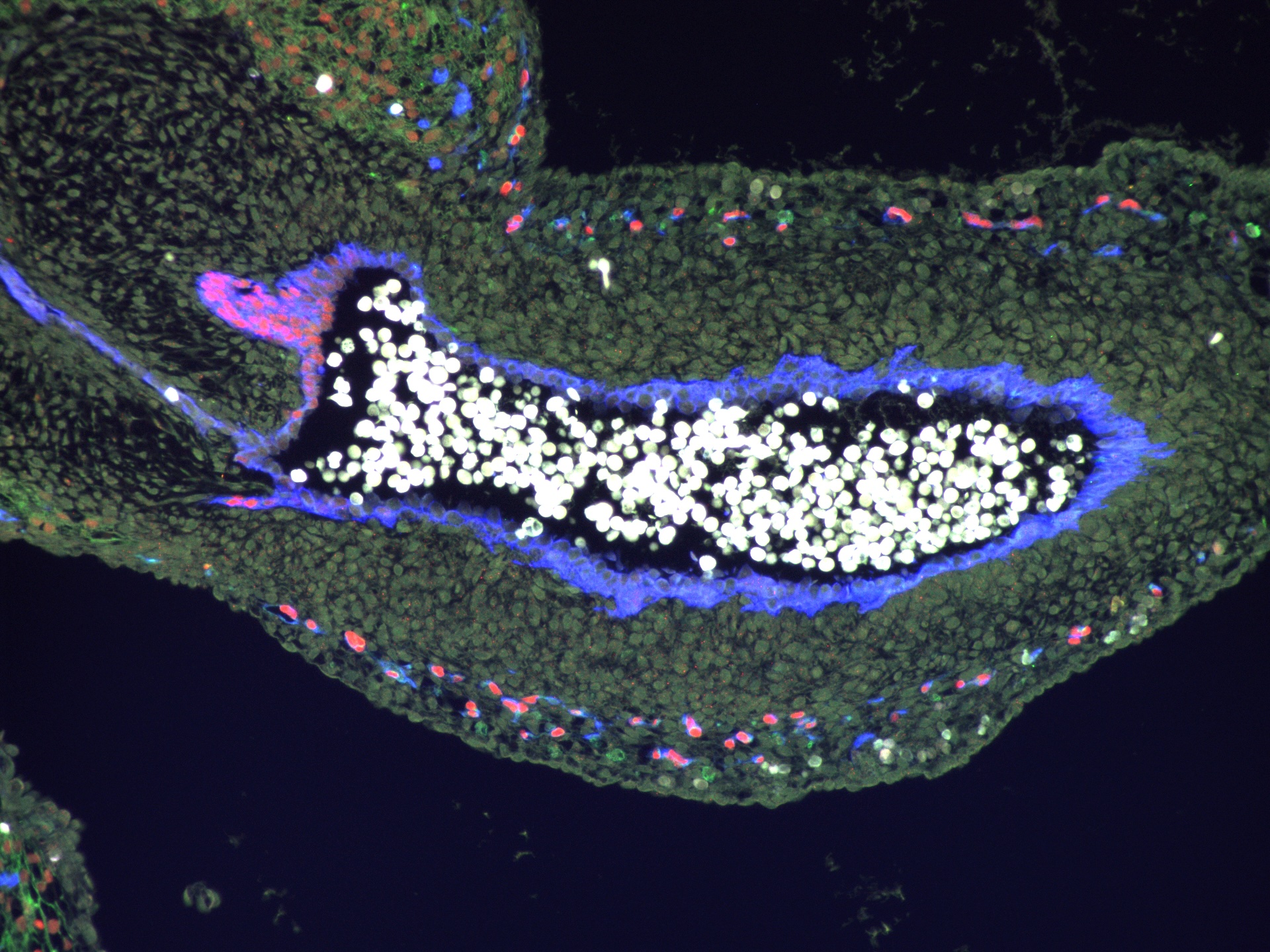

Supplement: Supplementary file 3 — Source Data Fig. 3 [file 44318_2024_45_MOESM3_ESM.zip › Figure3/Figure-3A.jpeg]

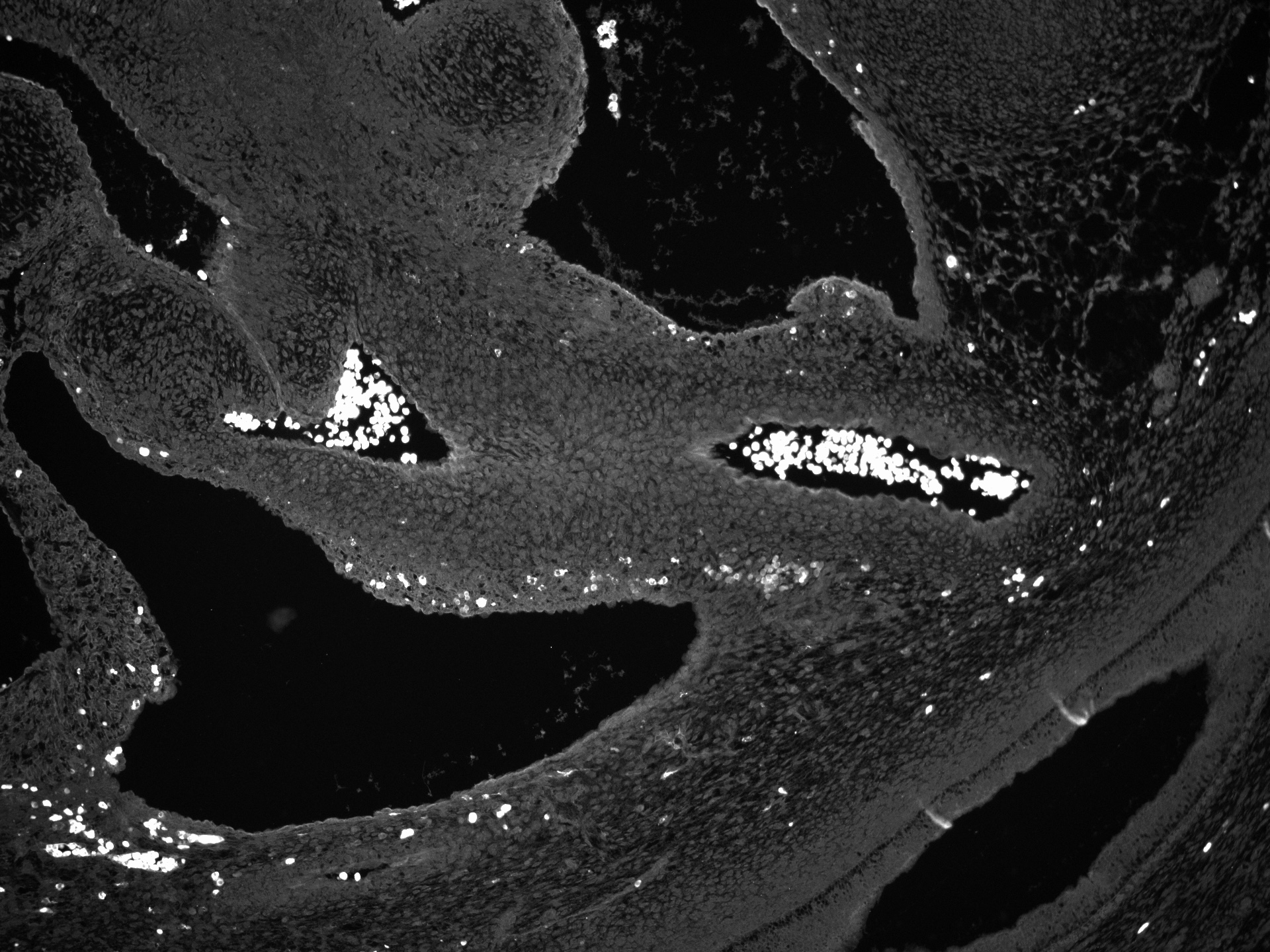

Supplement: Supplementary file 3 — Source Data Fig. 3 [file 44318_2024_45_MOESM3_ESM.zip › Figure3/Figure-3C.jpeg]

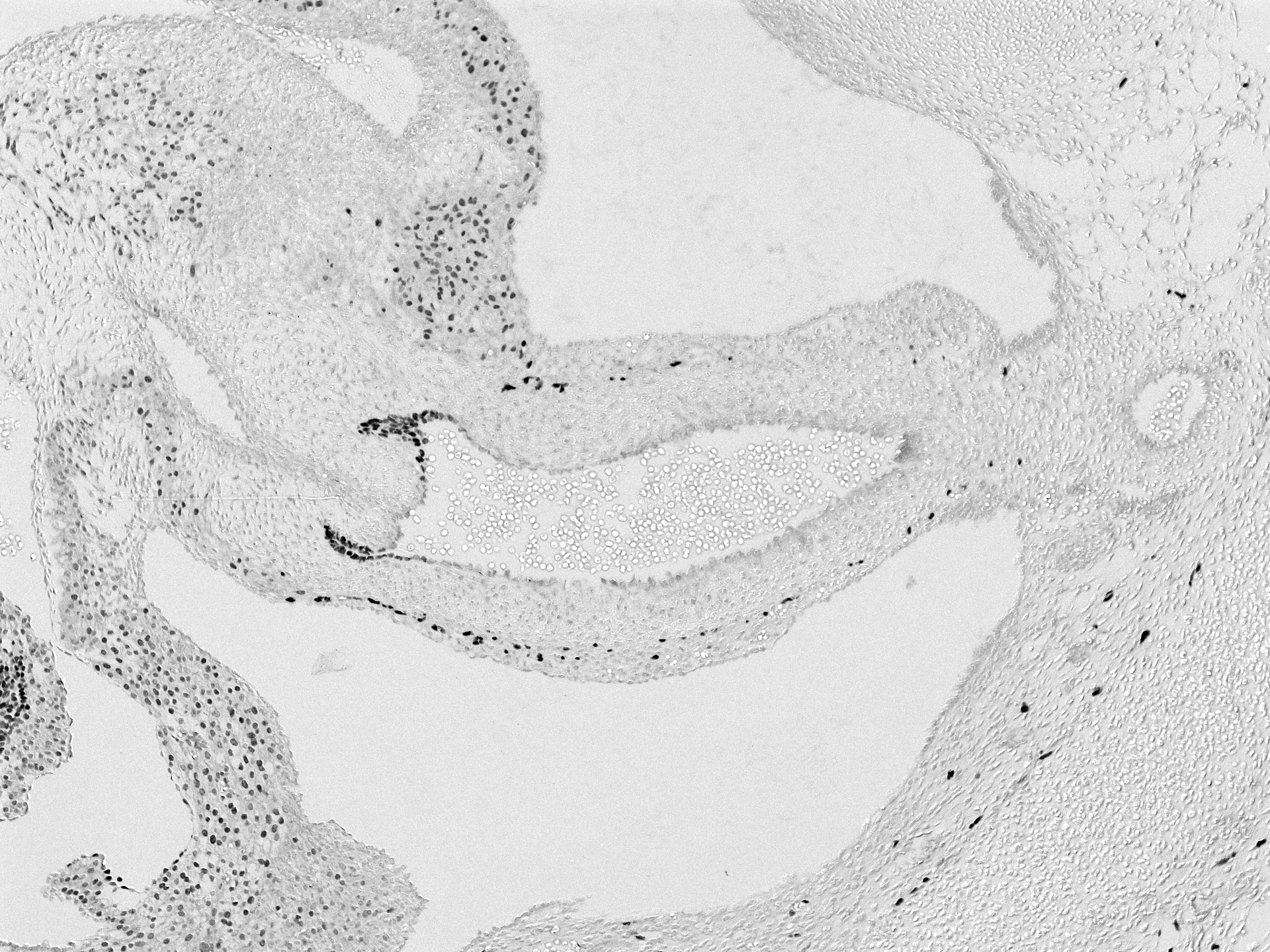

Supplement: Supplementary file 3 — Source Data Fig. 3 [file 44318_2024_45_MOESM3_ESM.zip › Figure3/Figure-3D.jpeg]

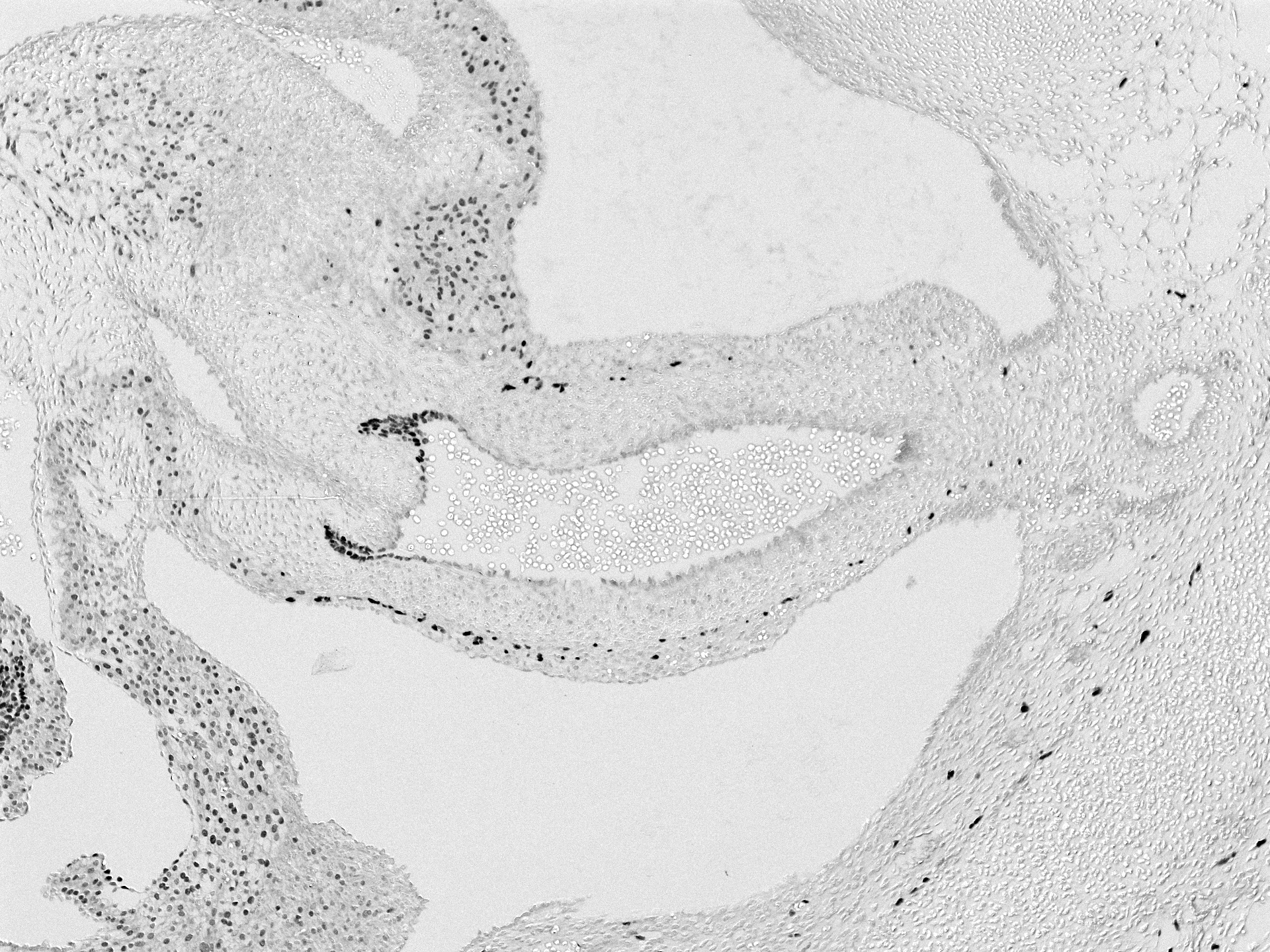

Supplement: Supplementary file 3 — Source Data Fig. 3 [file 44318_2024_45_MOESM3_ESM.zip › Figure3/Figure-3D.jpg]

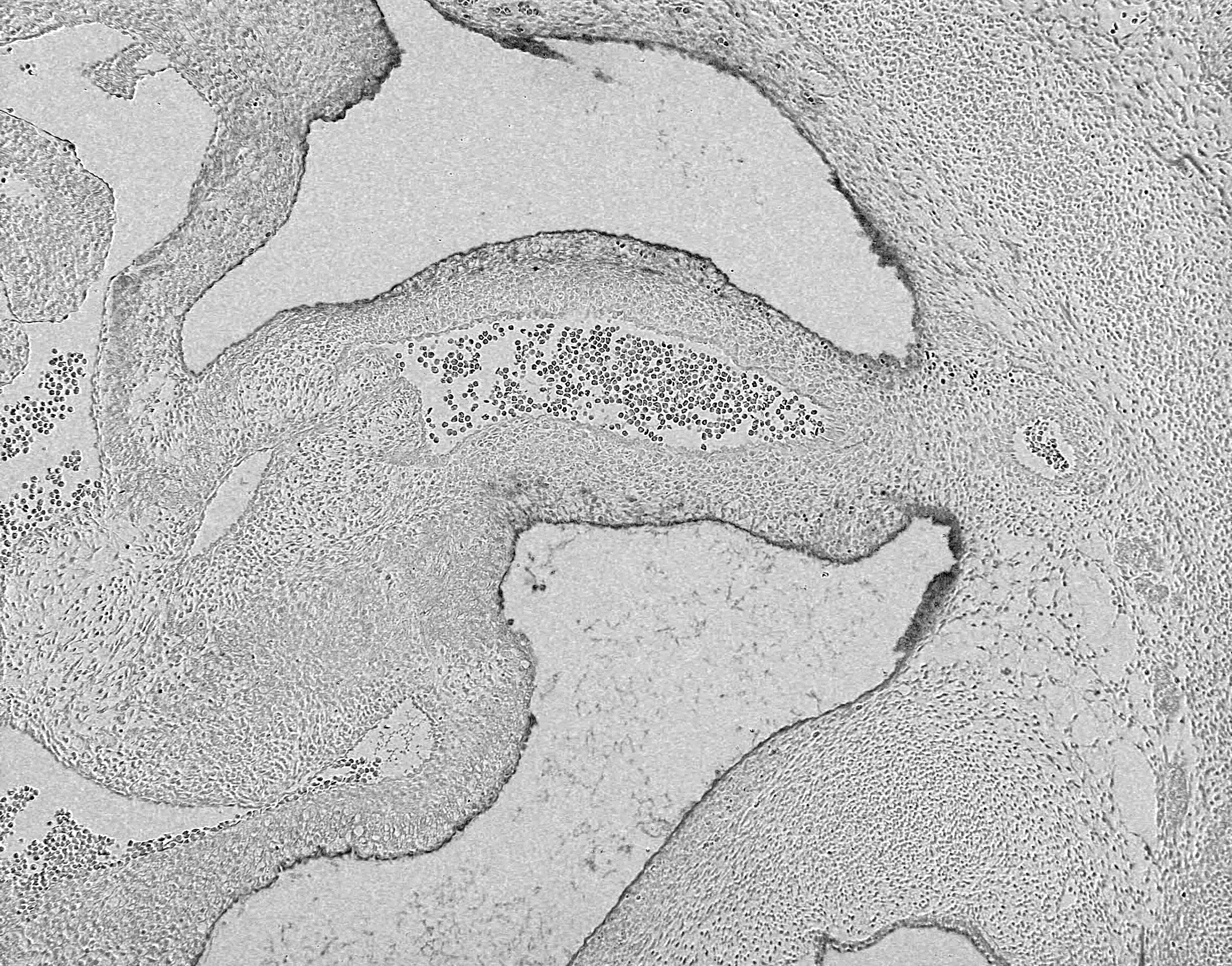

Supplement: Supplementary file 3 — Source Data Fig. 3 [file 44318_2024_45_MOESM3_ESM.zip › Figure3/Figure-3E.jpeg]

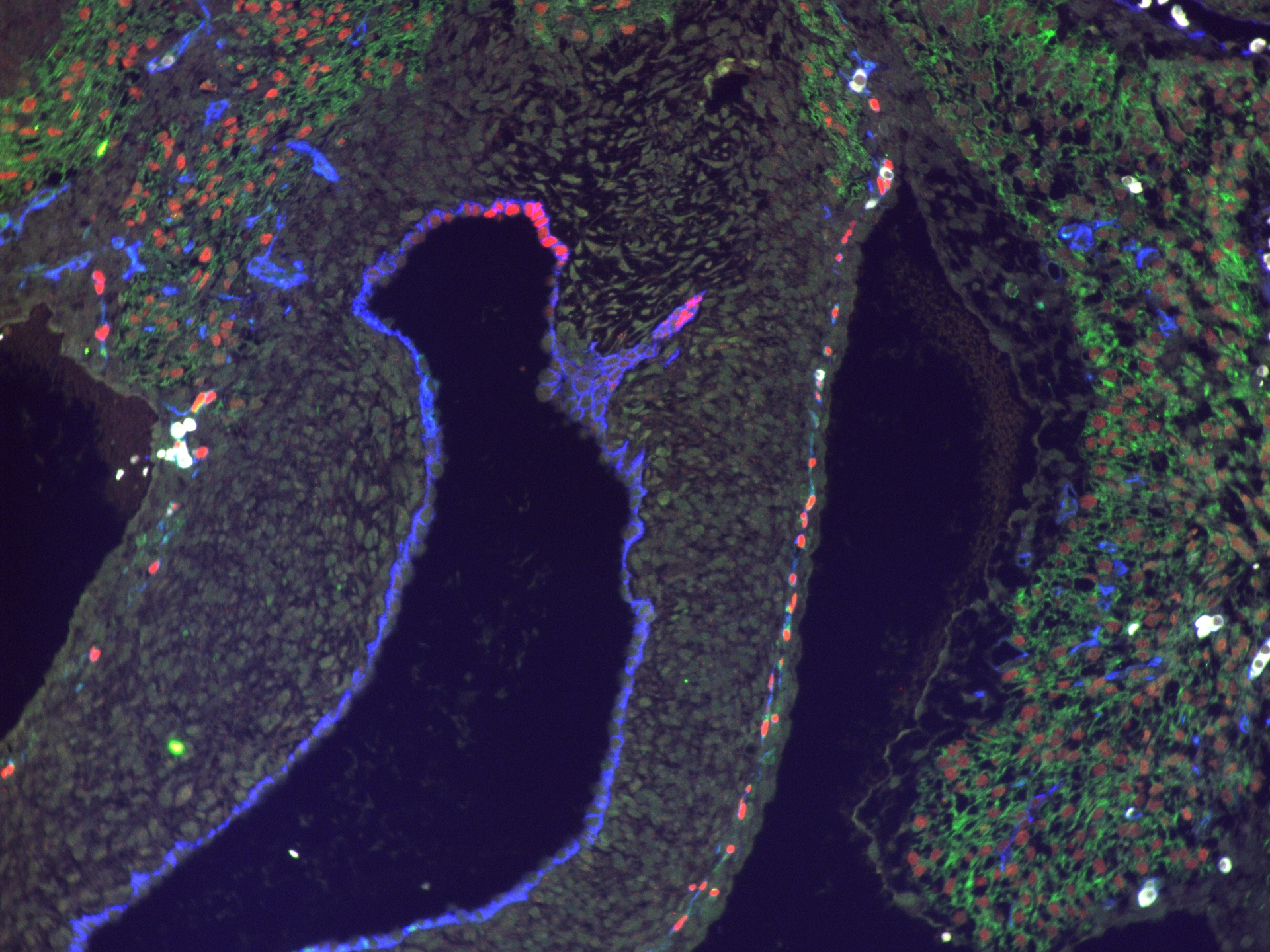

Supplement: Supplementary file 3 — Source Data Fig. 3 [file 44318_2024_45_MOESM3_ESM.zip › Figure3/Figure-3F.jpeg]

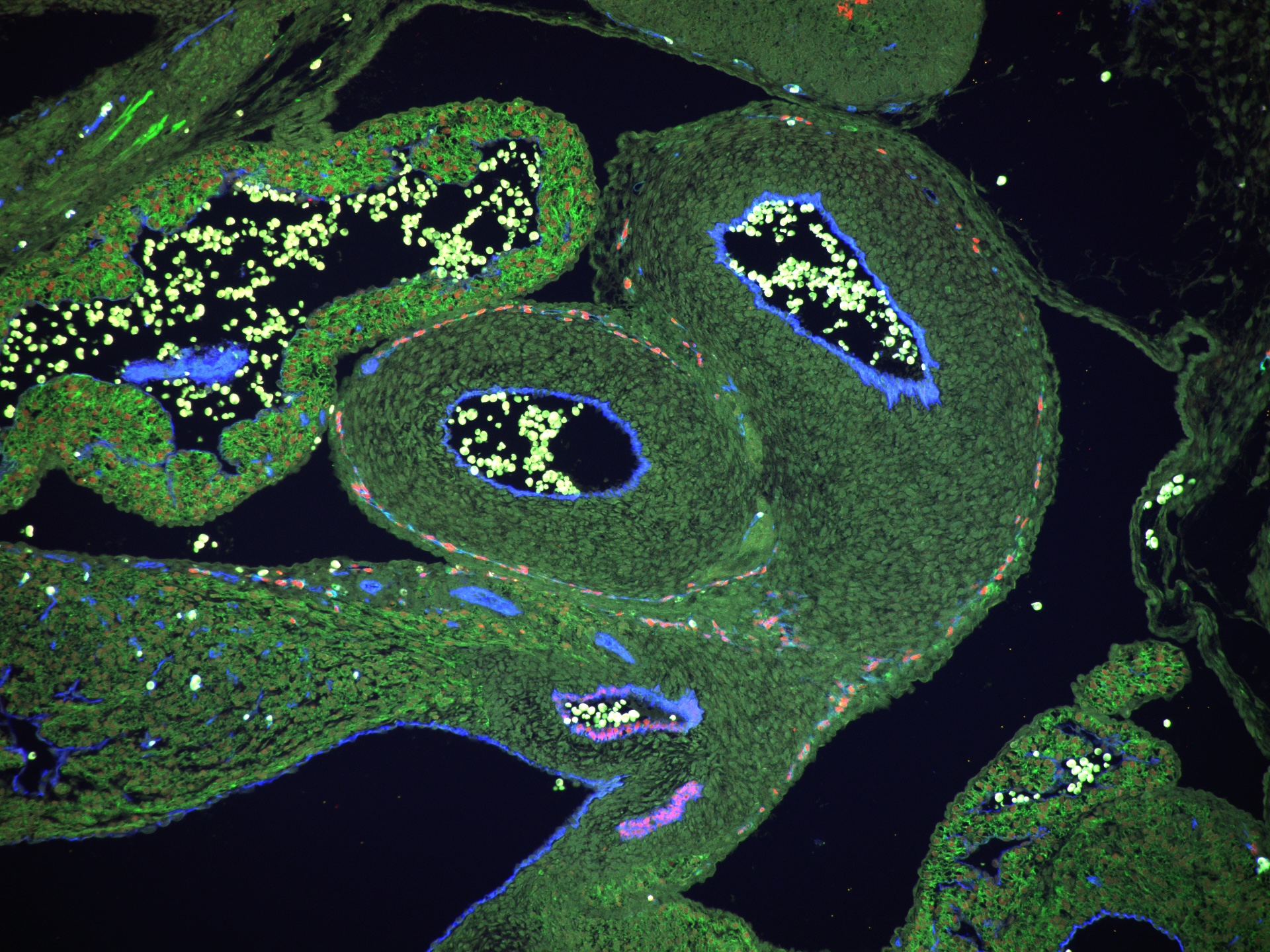

Supplement: Supplementary file 3 — Source Data Fig. 3 [file 44318_2024_45_MOESM3_ESM.zip › Figure3/Figure-3H.jpeg]

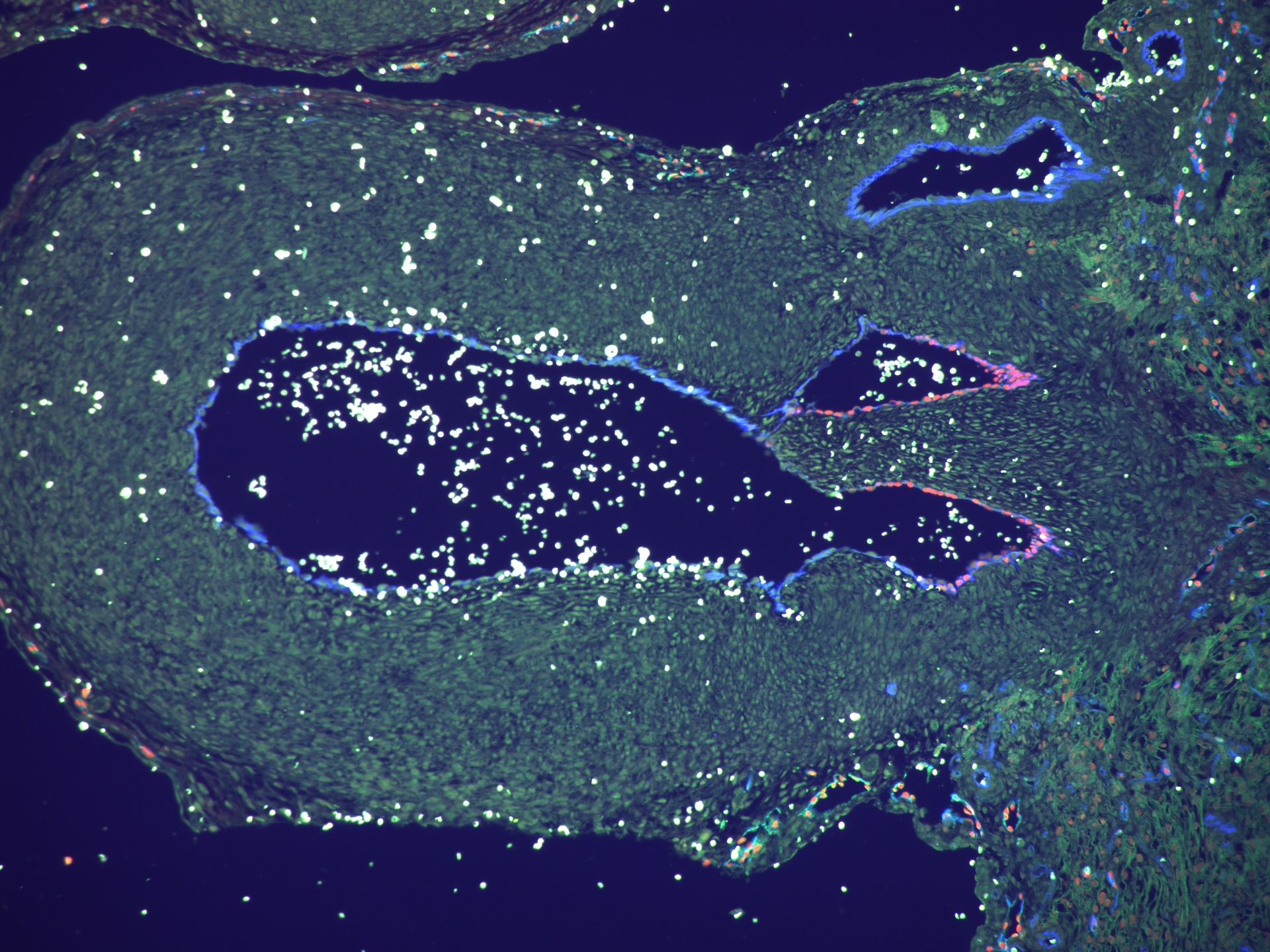

Supplement: Supplementary file 3 — Source Data Fig. 3 [file 44318_2024_45_MOESM3_ESM.zip › Figure3/Figure-3J.jpeg]

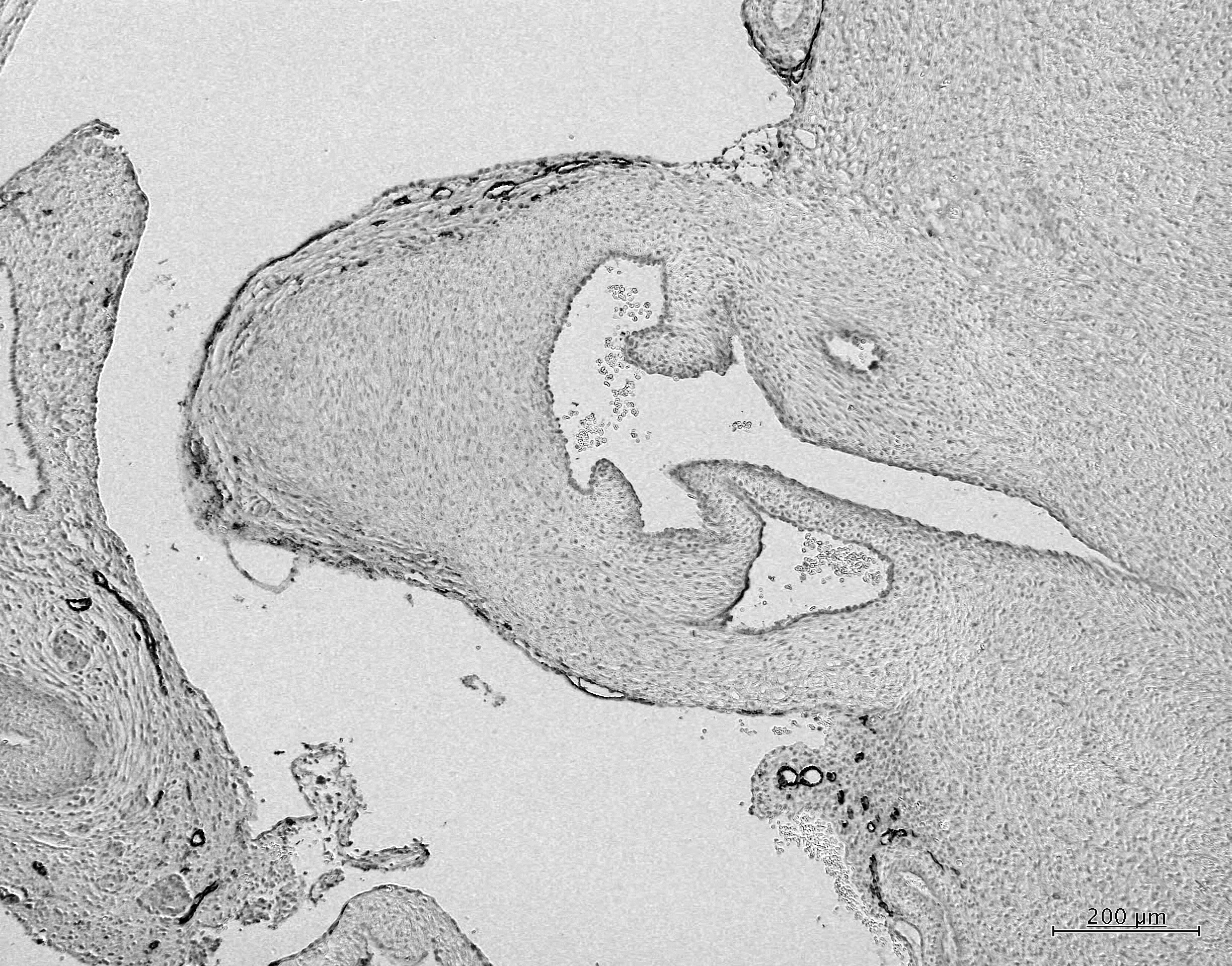

Supplement: Supplementary file 3 — Source Data Fig. 3 [file 44318_2024_45_MOESM3_ESM.zip › Figure3/Figure-3L.jpeg]

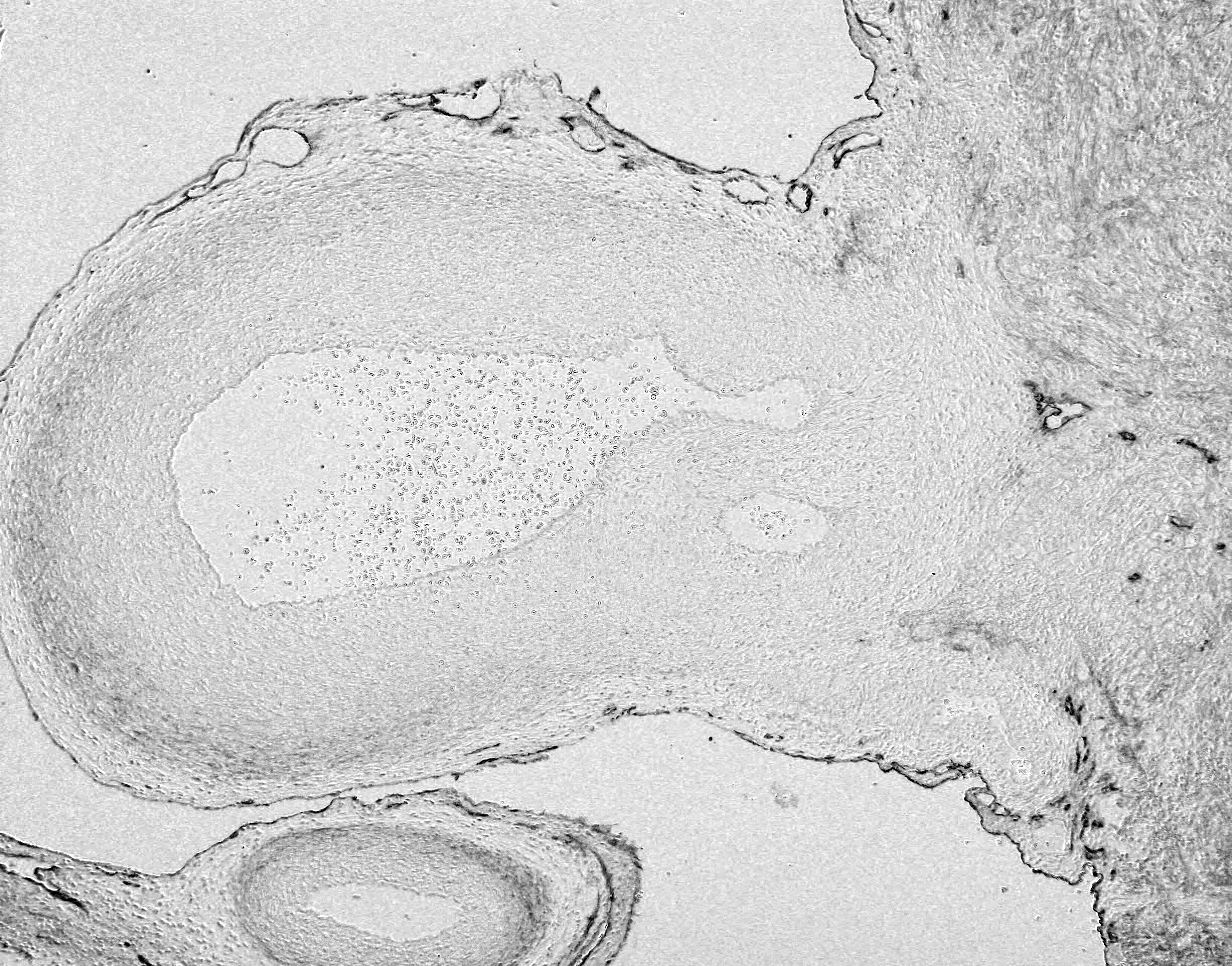

Supplement: Supplementary file 3 — Source Data Fig. 3 [file 44318_2024_45_MOESM3_ESM.zip › Figure3/Figure-3M.jpeg]

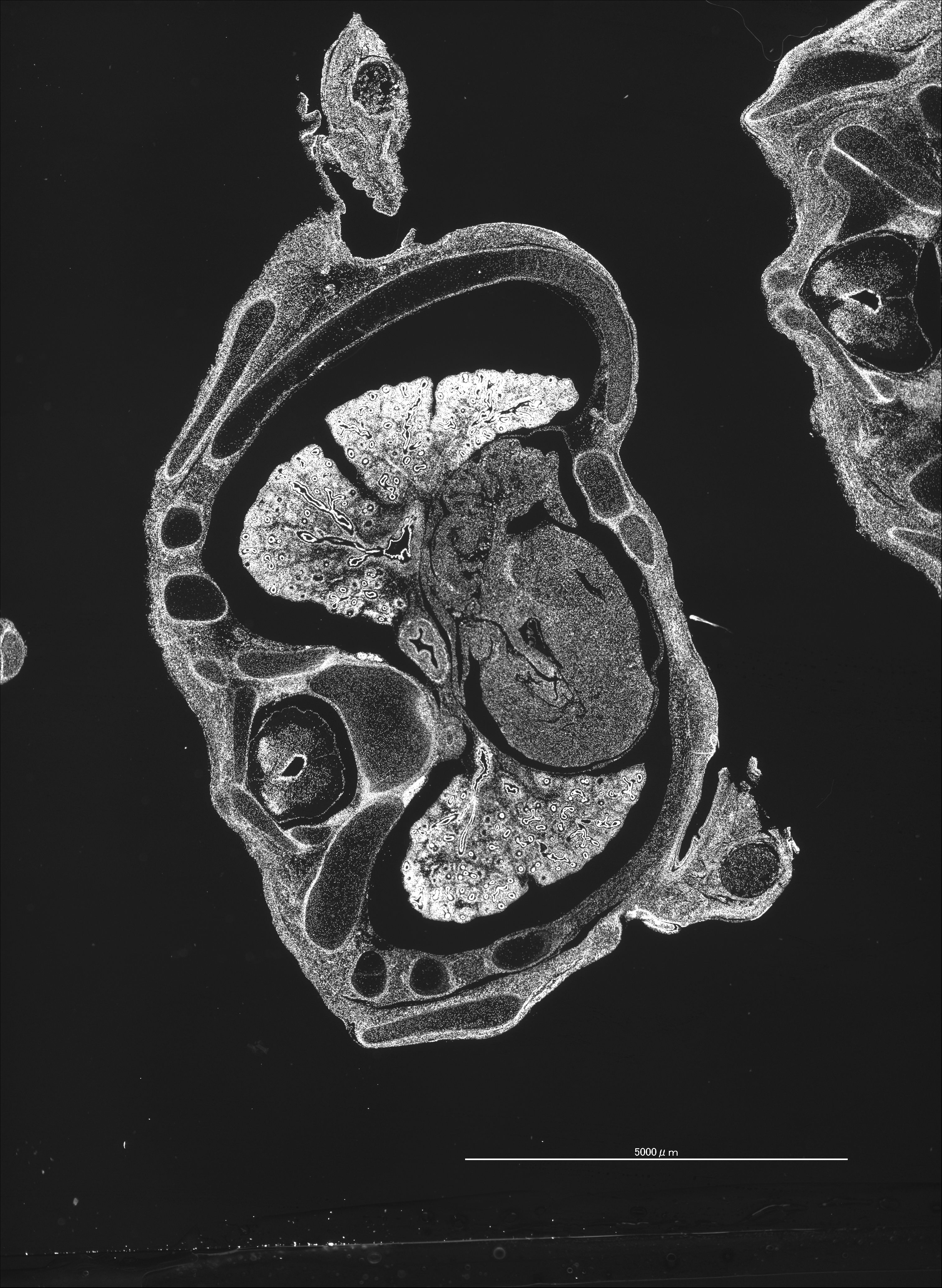

Supplement: Supplementary file 3 — Source Data Fig. 3 [file 44318_2024_45_MOESM3_ESM.zip › Figure3/Figure-3P.jpeg]

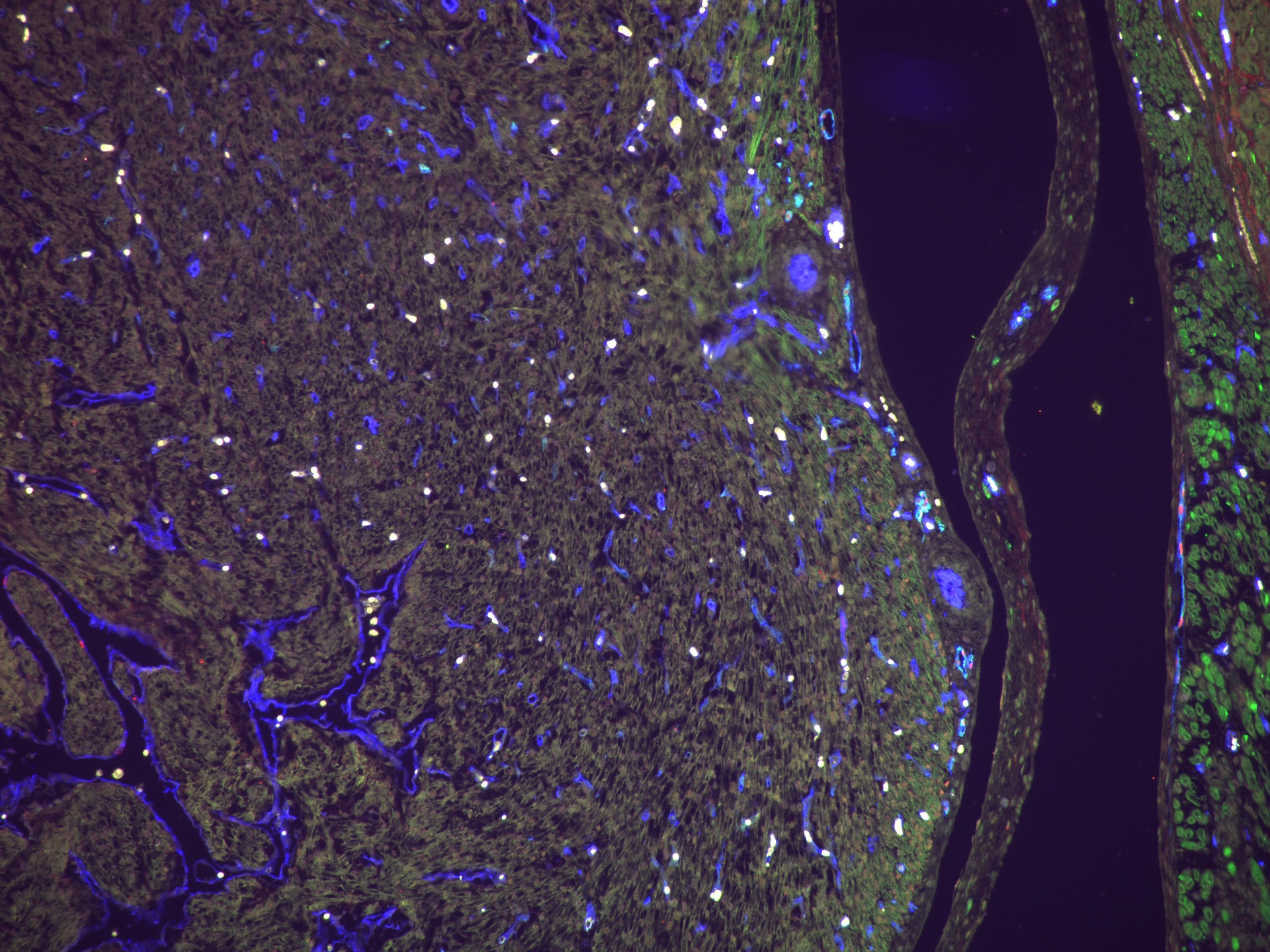

Supplement: Supplementary file 3 — Source Data Fig. 3 [file 44318_2024_45_MOESM3_ESM.zip › Figure3/Figure-3Q.jpeg]

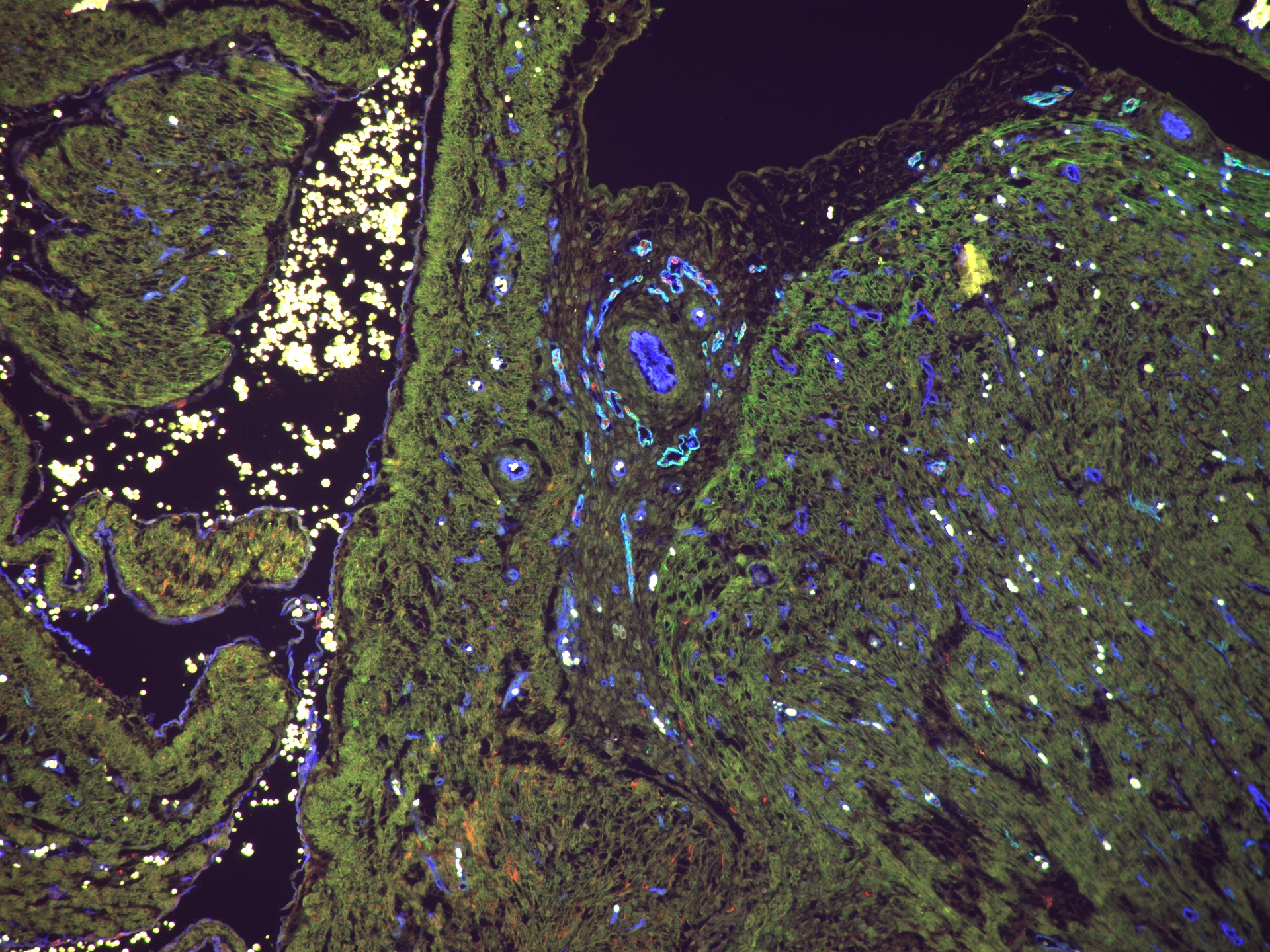

Supplement: Supplementary file 3 — Source Data Fig. 3 [file 44318_2024_45_MOESM3_ESM.zip › Figure3/Figure-3R.jpeg]

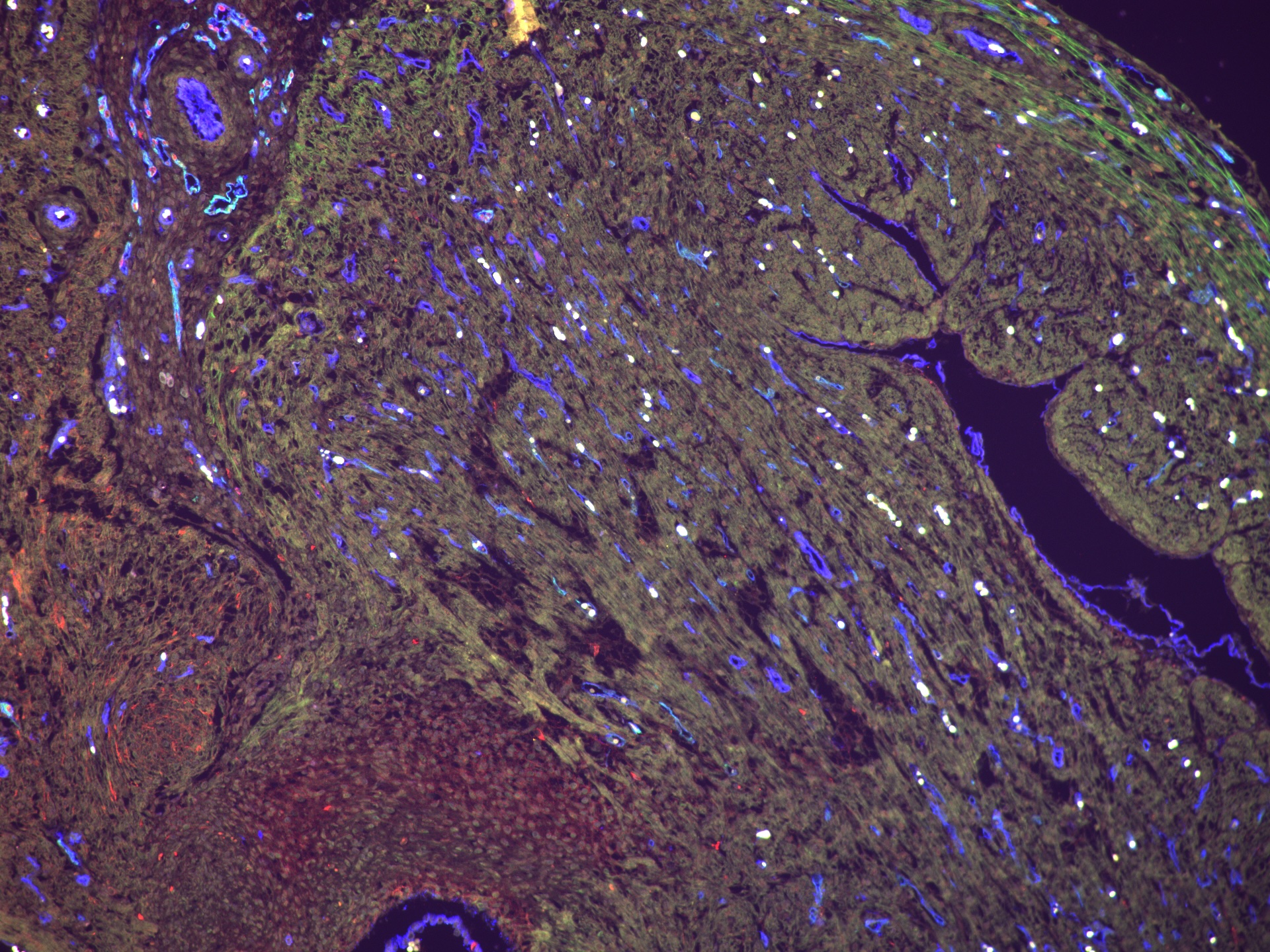

Supplement: Supplementary file 3 — Source Data Fig. 3 [file 44318_2024_45_MOESM3_ESM.zip › Figure3/Figure-3S.jpeg]

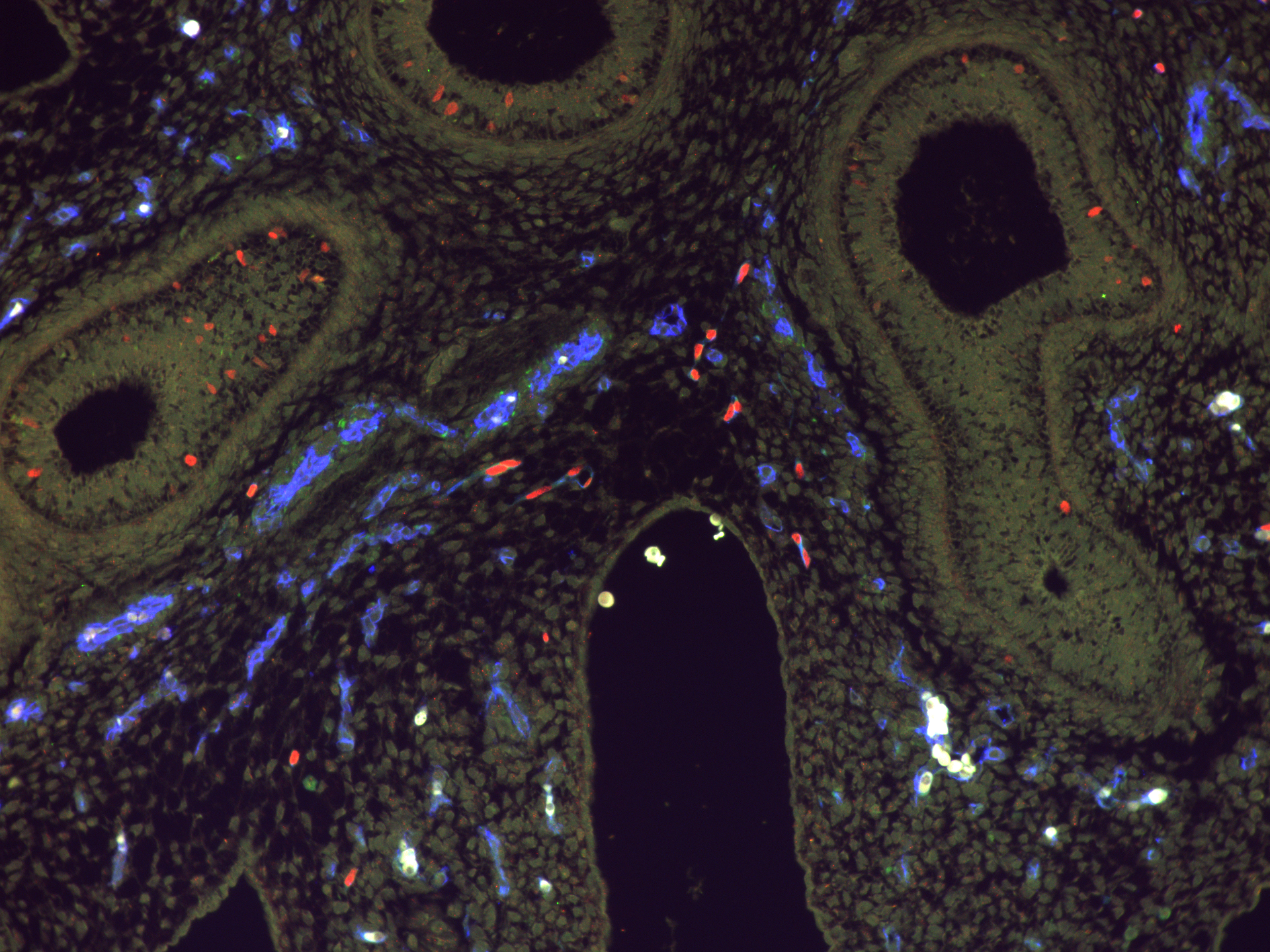

Supplement: Supplementary file 4 — Source Data Fig. 4 [file 44318_2024_45_MOESM4_ESM.zip › Figure4/Figure-4A.jpeg]

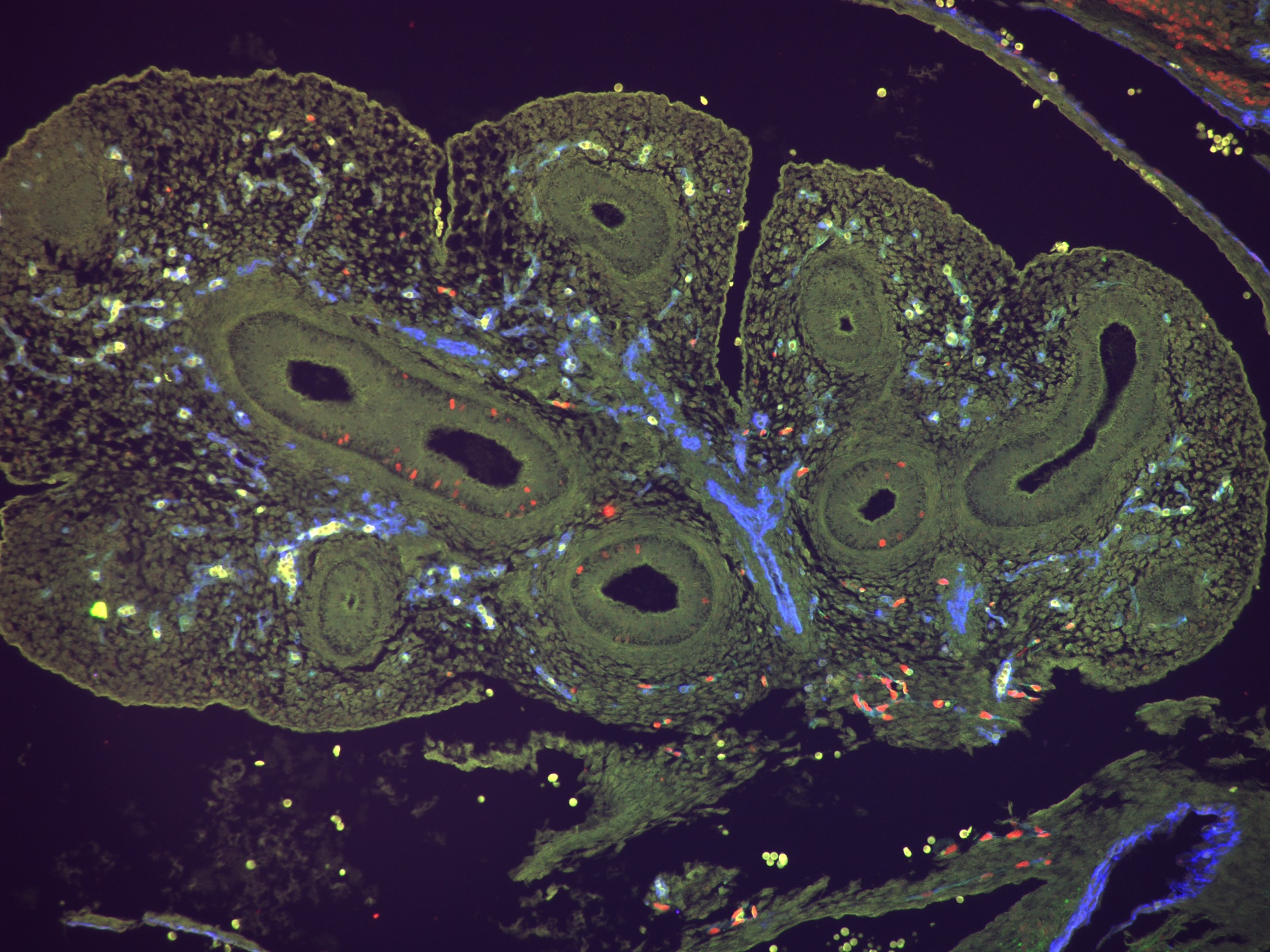

Supplement: Supplementary file 4 — Source Data Fig. 4 [file 44318_2024_45_MOESM4_ESM.zip › Figure4/Figure-4B.jpeg]

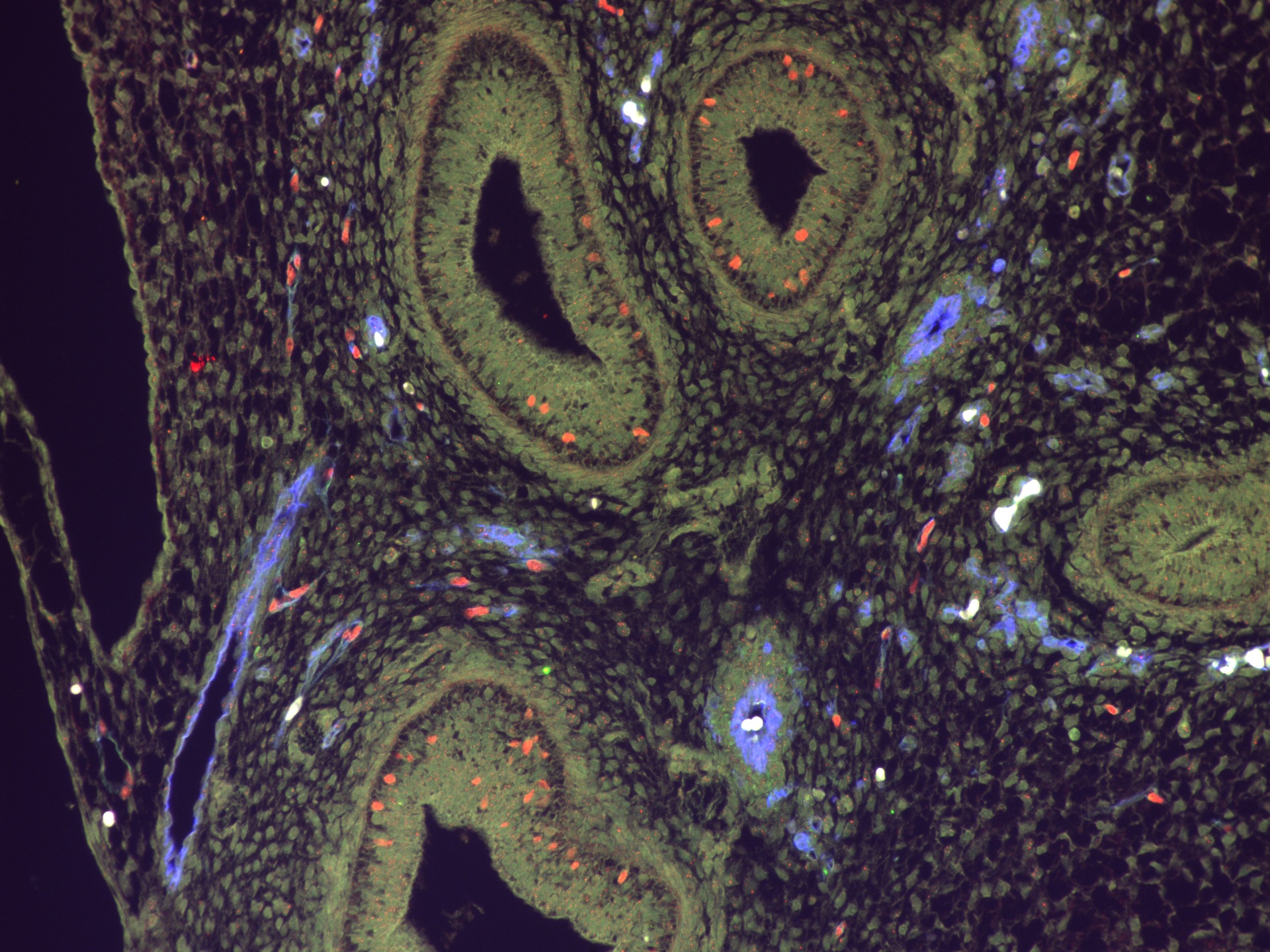

Supplement: Supplementary file 4 — Source Data Fig. 4 [file 44318_2024_45_MOESM4_ESM.zip › Figure4/Figure-4C.jpeg]

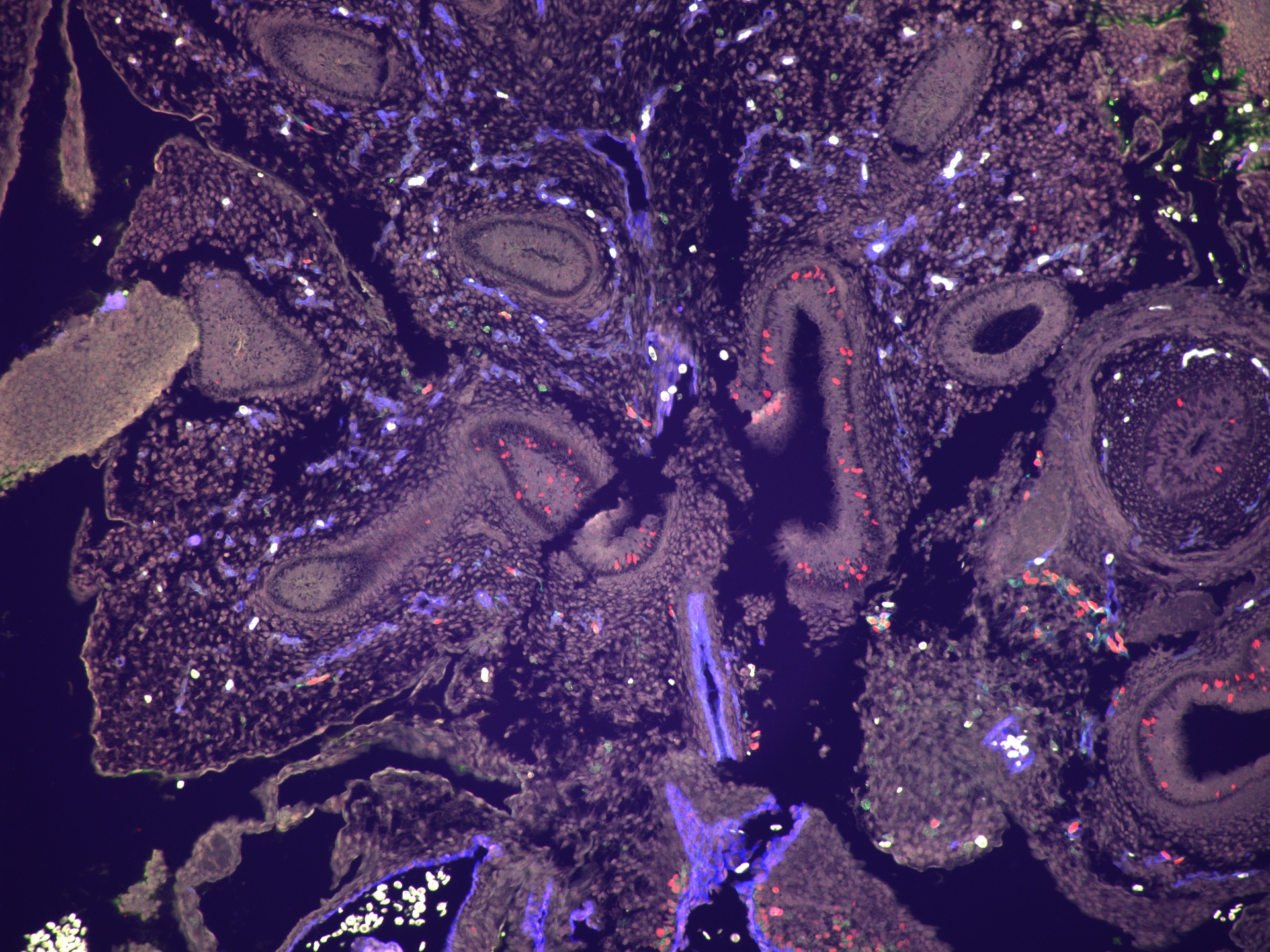

Supplement: Supplementary file 4 — Source Data Fig. 4 [file 44318_2024_45_MOESM4_ESM.zip › Figure4/Figure-4D.jpeg]

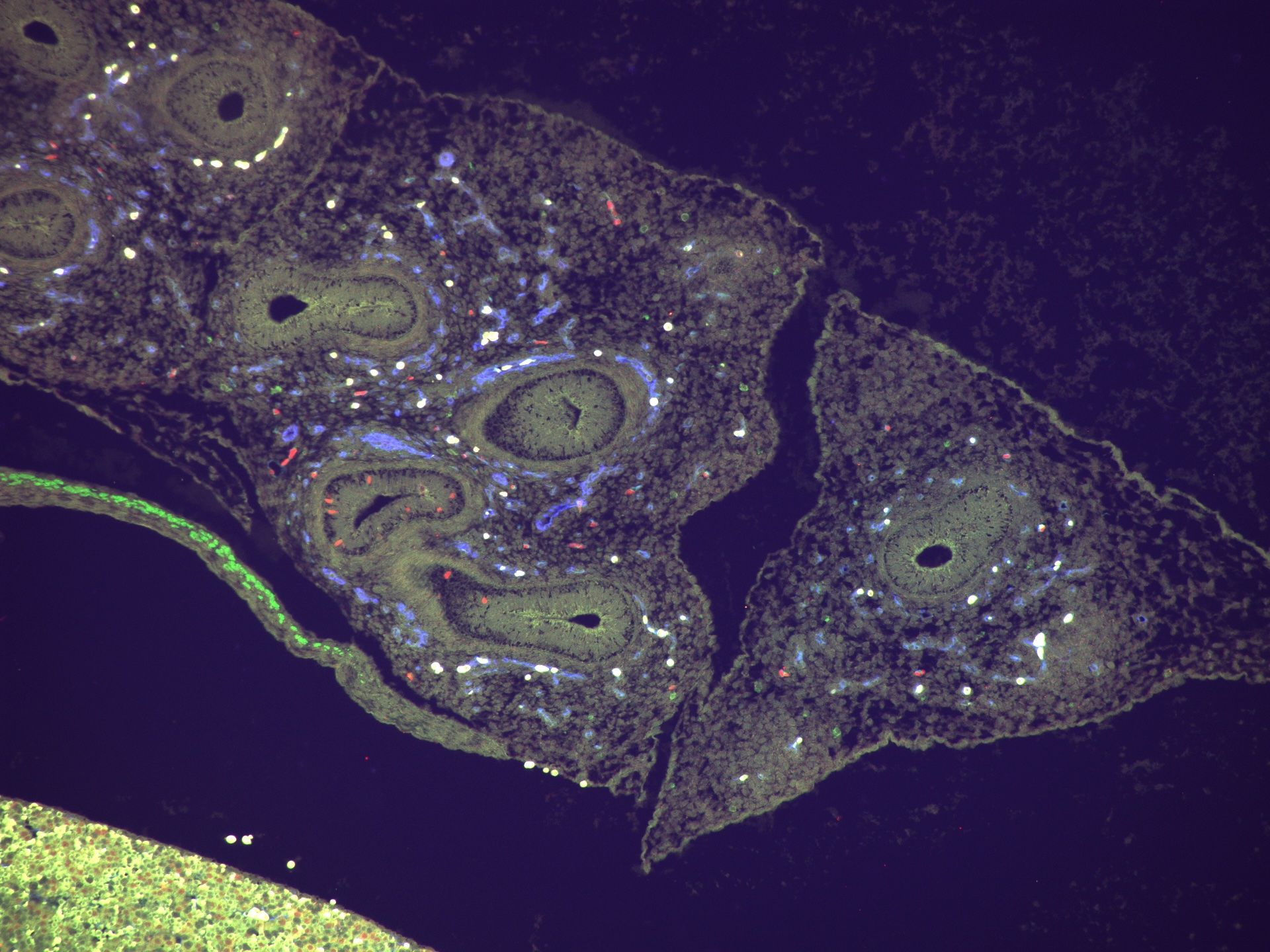

Supplement: Supplementary file 4 — Source Data Fig. 4 [file 44318_2024_45_MOESM4_ESM.zip › Figure4/Figure-4E.jpeg]

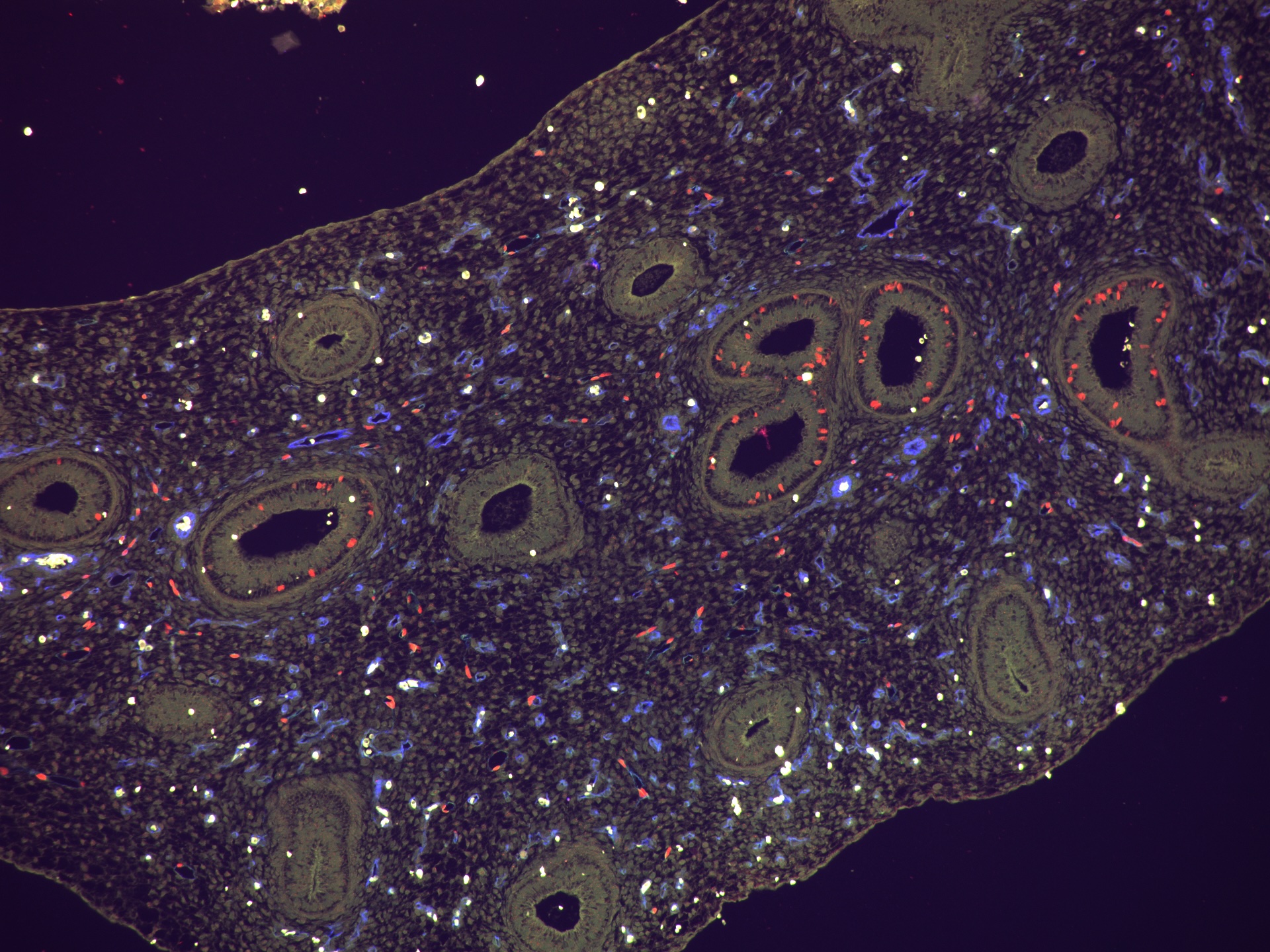

Supplement: Supplementary file 4 — Source Data Fig. 4 [file 44318_2024_45_MOESM4_ESM.zip › Figure4/Figure-4F.jpeg]

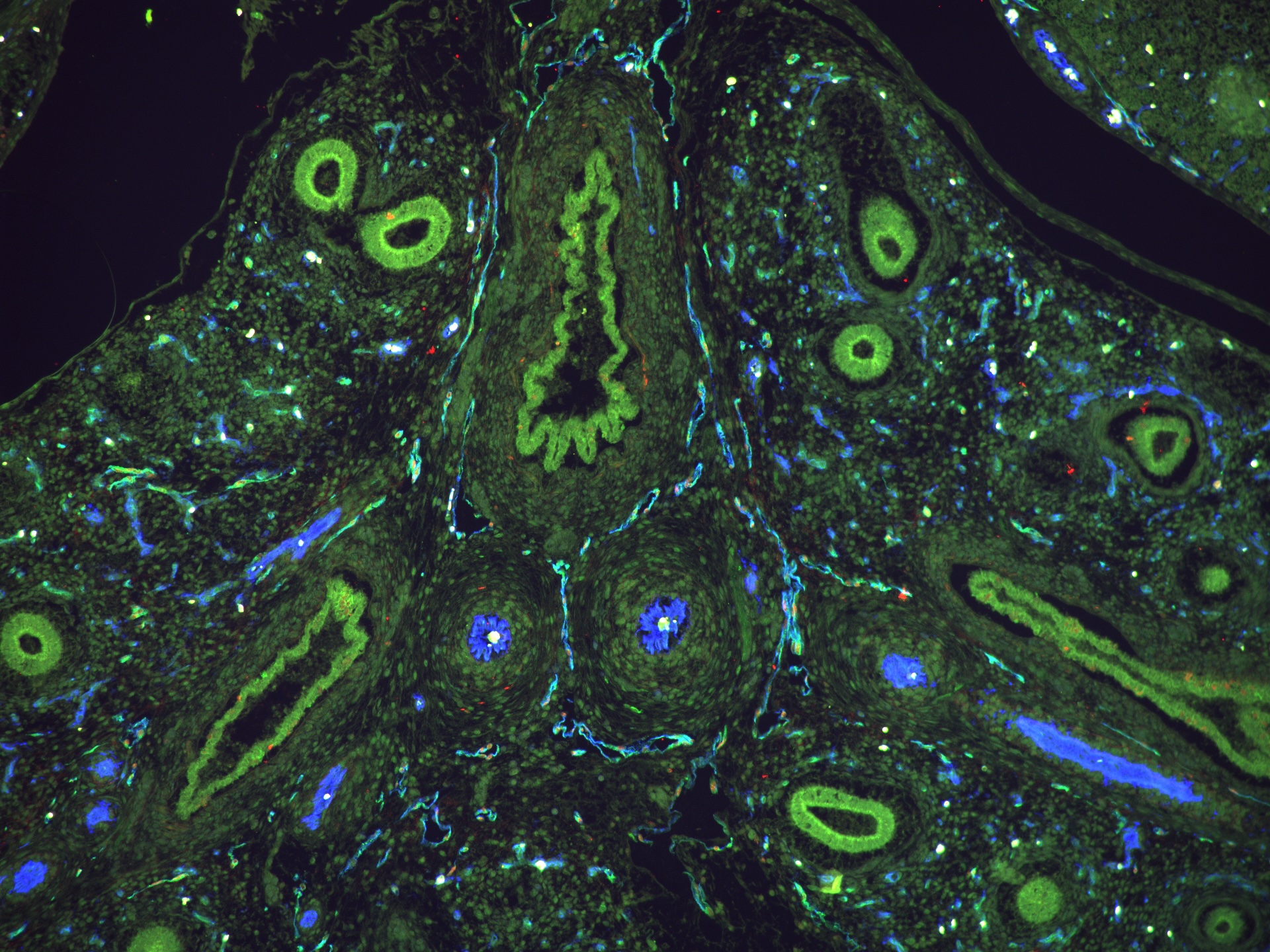

Supplement: Supplementary file 4 — Source Data Fig. 4 [file 44318_2024_45_MOESM4_ESM.zip › Figure4/Figure-4G.jpeg]

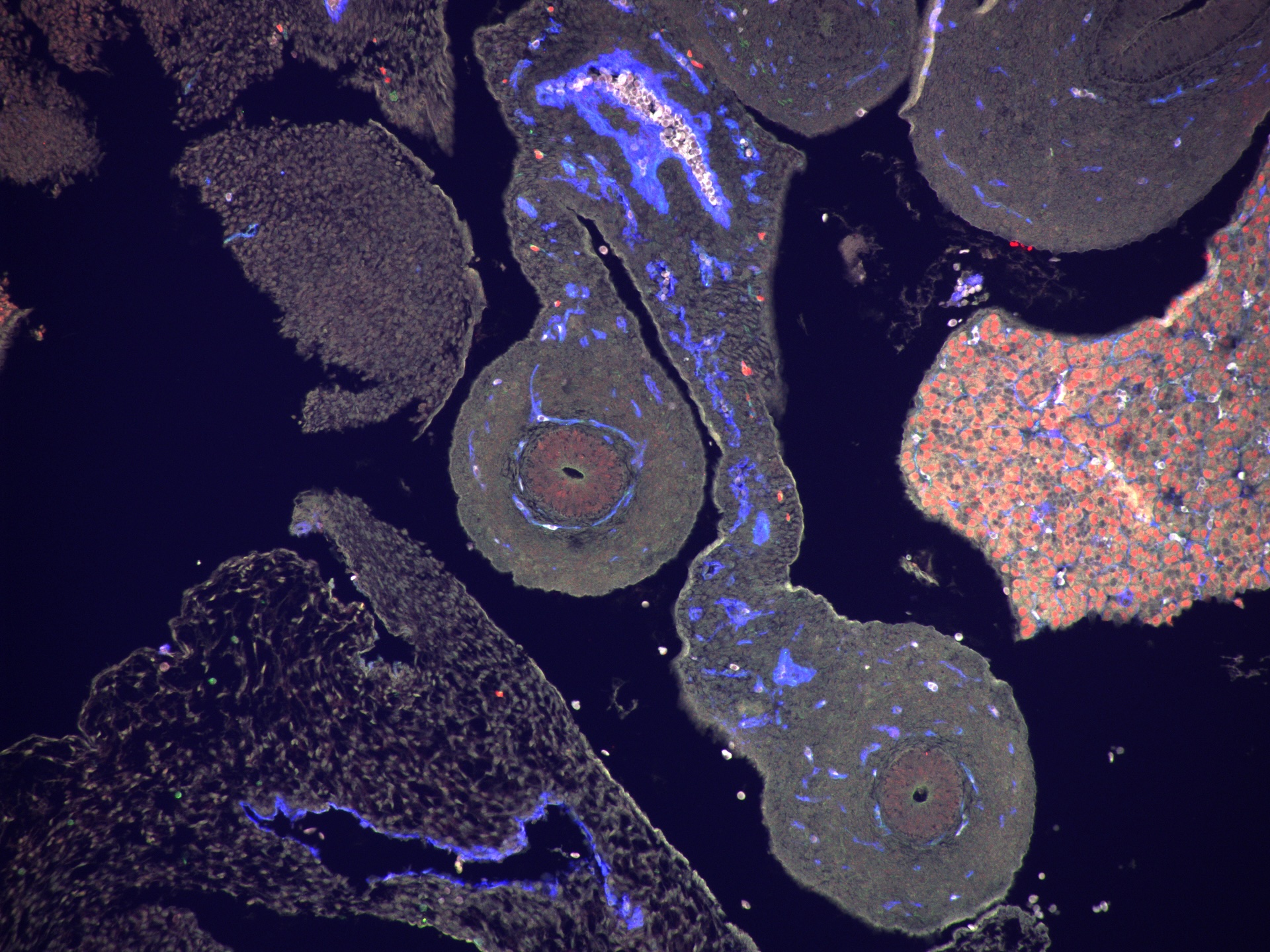

Supplement: Supplementary file 4 — Source Data Fig. 4 [file 44318_2024_45_MOESM4_ESM.zip › Figure4/Figure-4K.jpeg]

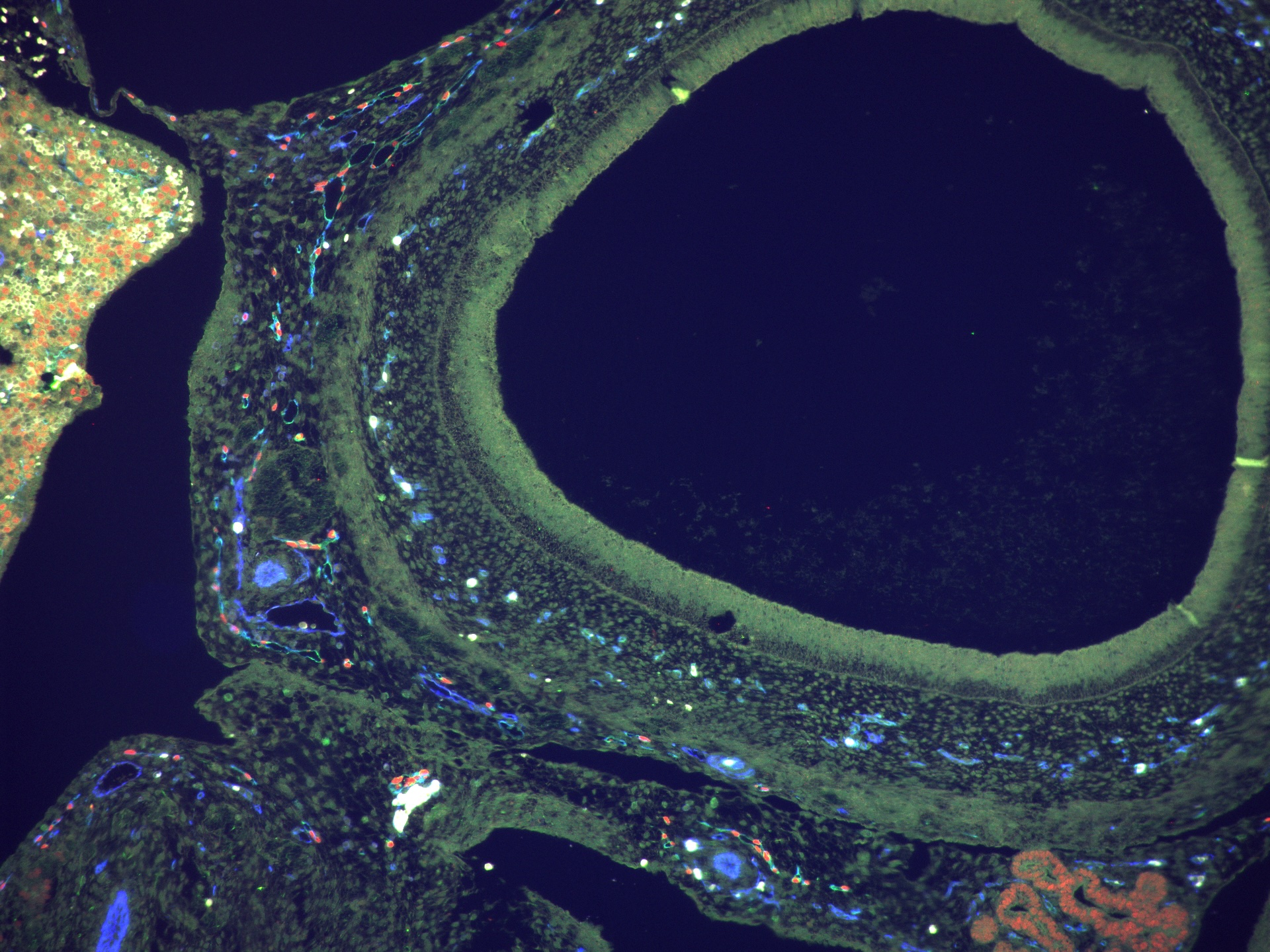

Supplement: Supplementary file 4 — Source Data Fig. 4 [file 44318_2024_45_MOESM4_ESM.zip › Figure4/Figure-4M.jpeg]

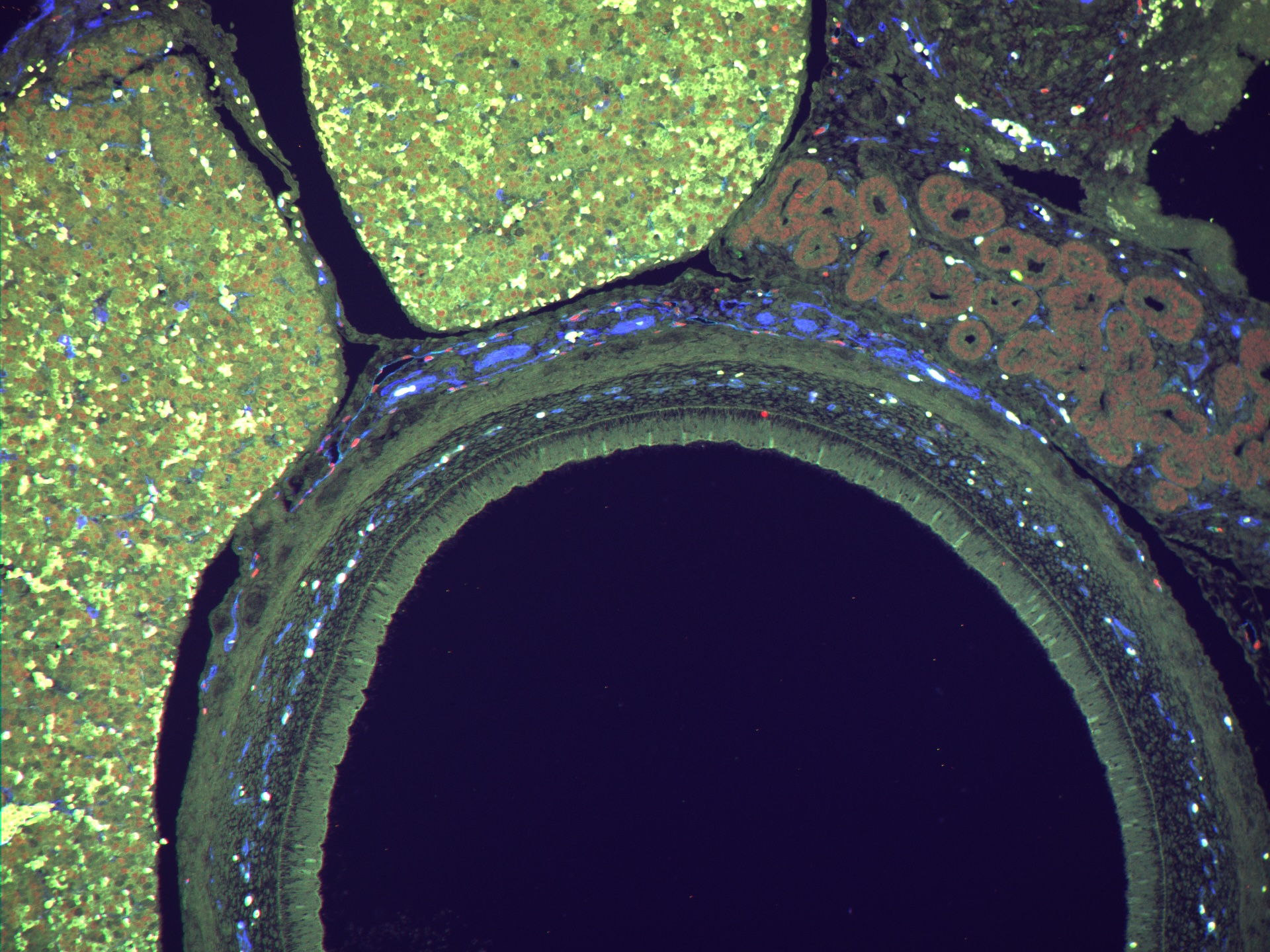

Supplement: Supplementary file 4 — Source Data Fig. 4 [file 44318_2024_45_MOESM4_ESM.zip › Figure4/Figure-4O.jpeg]

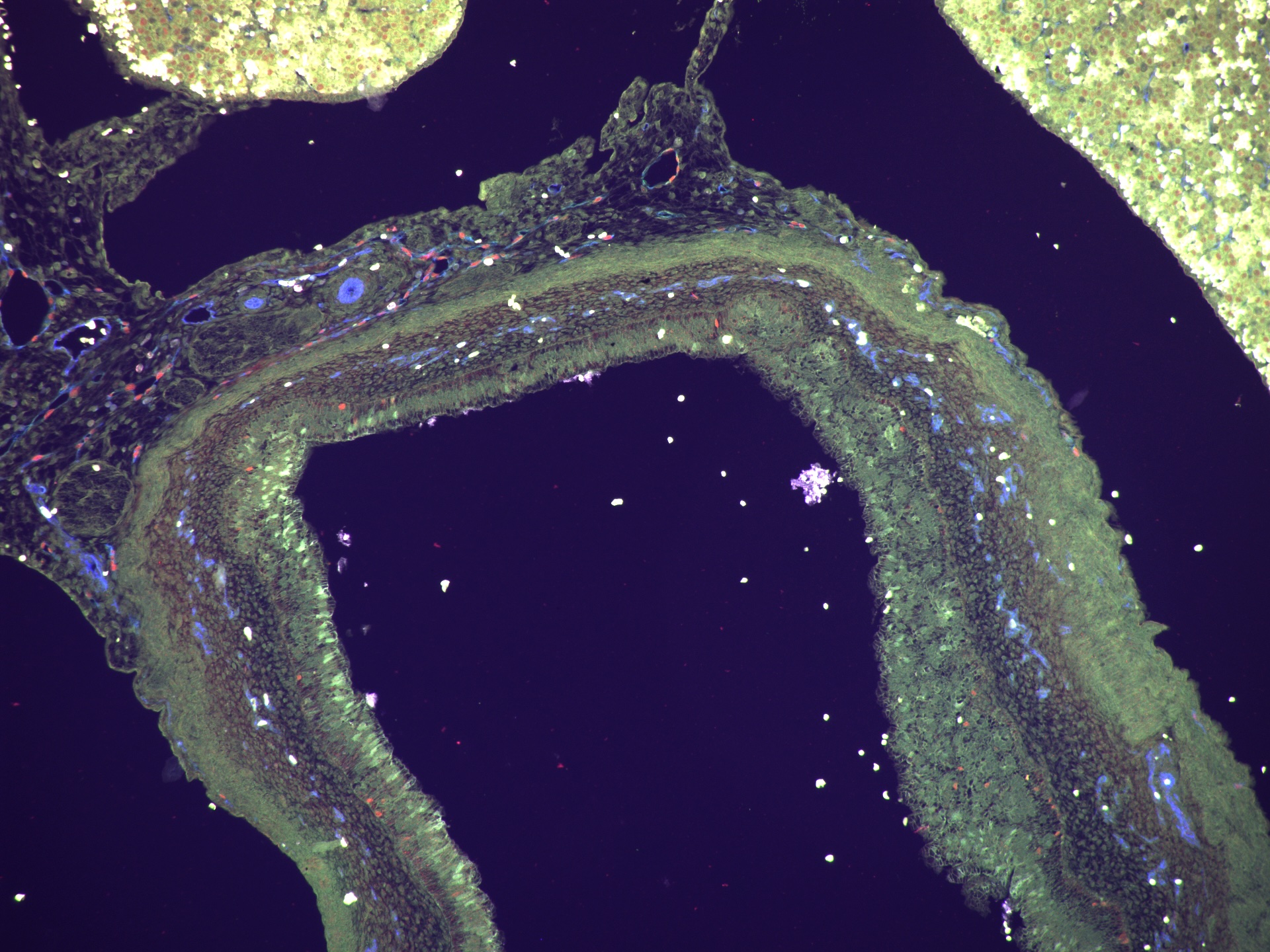

Supplement: Supplementary file 4 — Source Data Fig. 4 [file 44318_2024_45_MOESM4_ESM.zip › Figure4/Figure-4Q.jpeg]

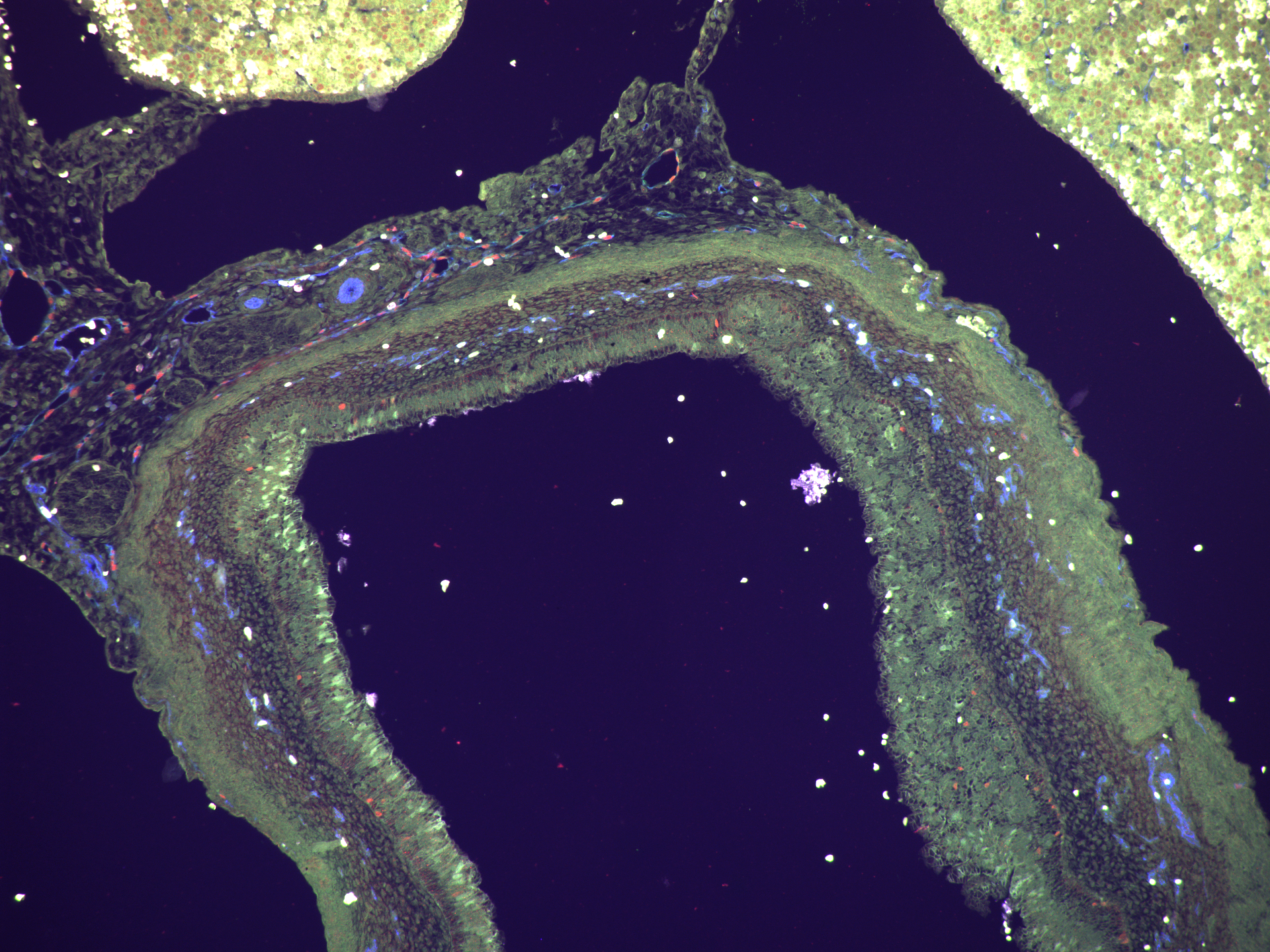

Supplement: Supplementary file 4 — Source Data Fig. 4 [file 44318_2024_45_MOESM4_ESM.zip › Figure4/Figure-4R.jpeg]

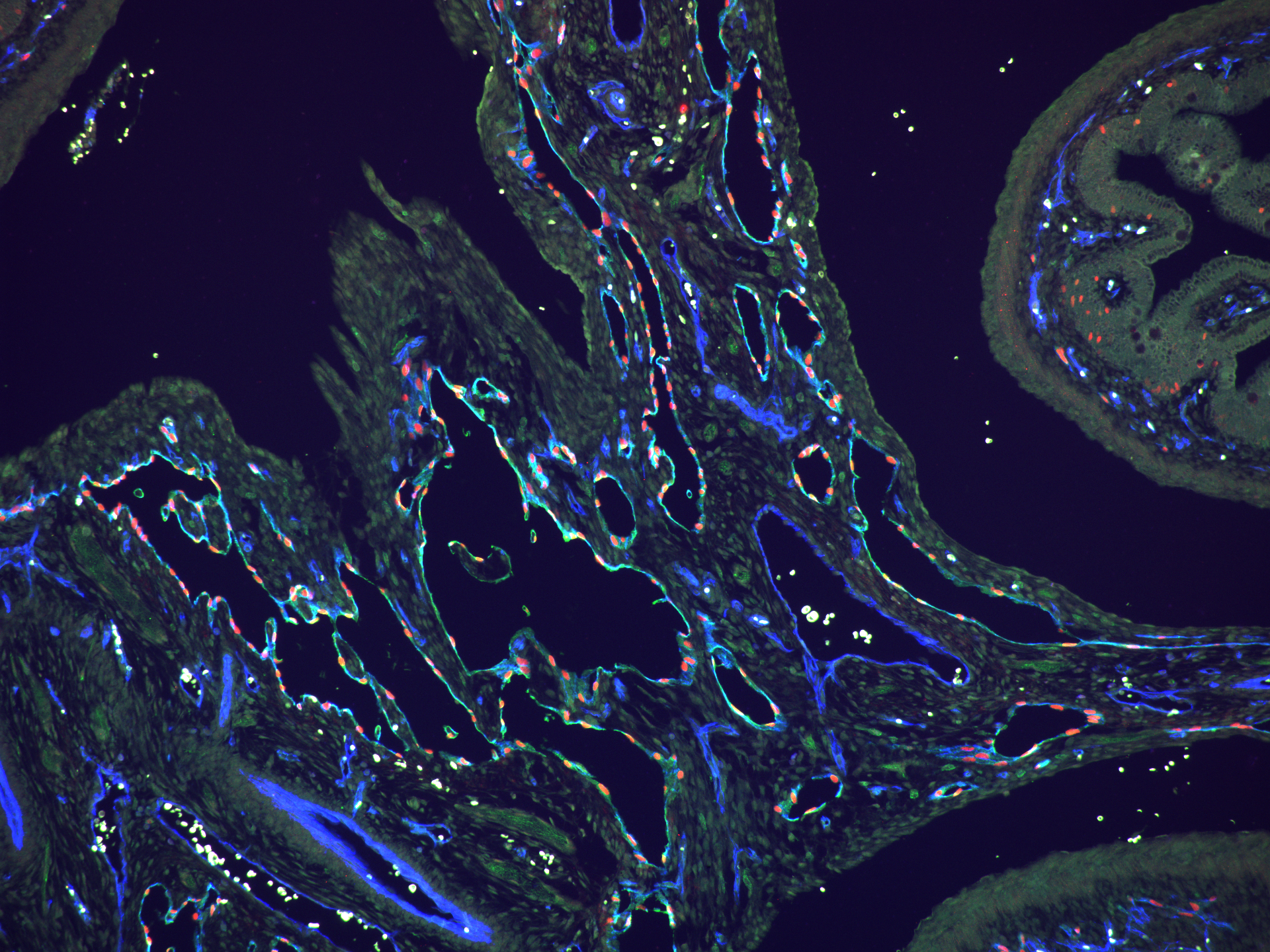

Supplement: Supplementary file 4 — Source Data Fig. 4 [file 44318_2024_45_MOESM4_ESM.zip › Figure4/Figure-4S.jpeg]

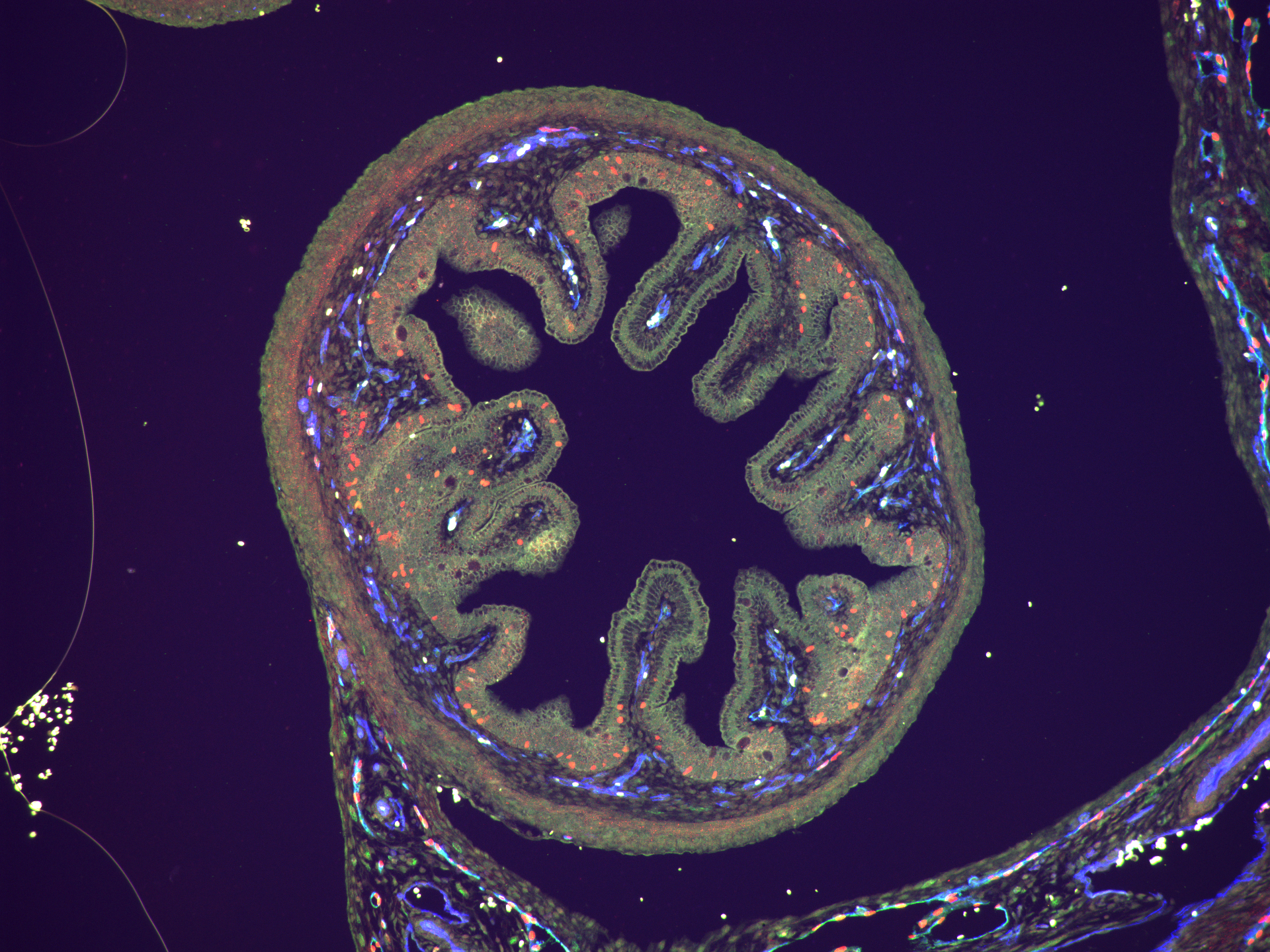

Supplement: Supplementary file 4 — Source Data Fig. 4 [file 44318_2024_45_MOESM4_ESM.zip › Figure4/Figure-4U.jpeg]
