# Supplementary material for: The development of early human lymphatic vessels as characterized by lymphatic endothelial markers
Source: EMBO J. 2024 Feb 14;43(5):868–85. doi: 10.1038/s44318-024-00045-0 (PMC10907744; doi:10.1038/s44318-024-00045-0)
Supplement: Supplementary file 6 — Expanded View Figures [file 44318_2024_45_MOESM6_ESM.pdf]

Expanded View Figures

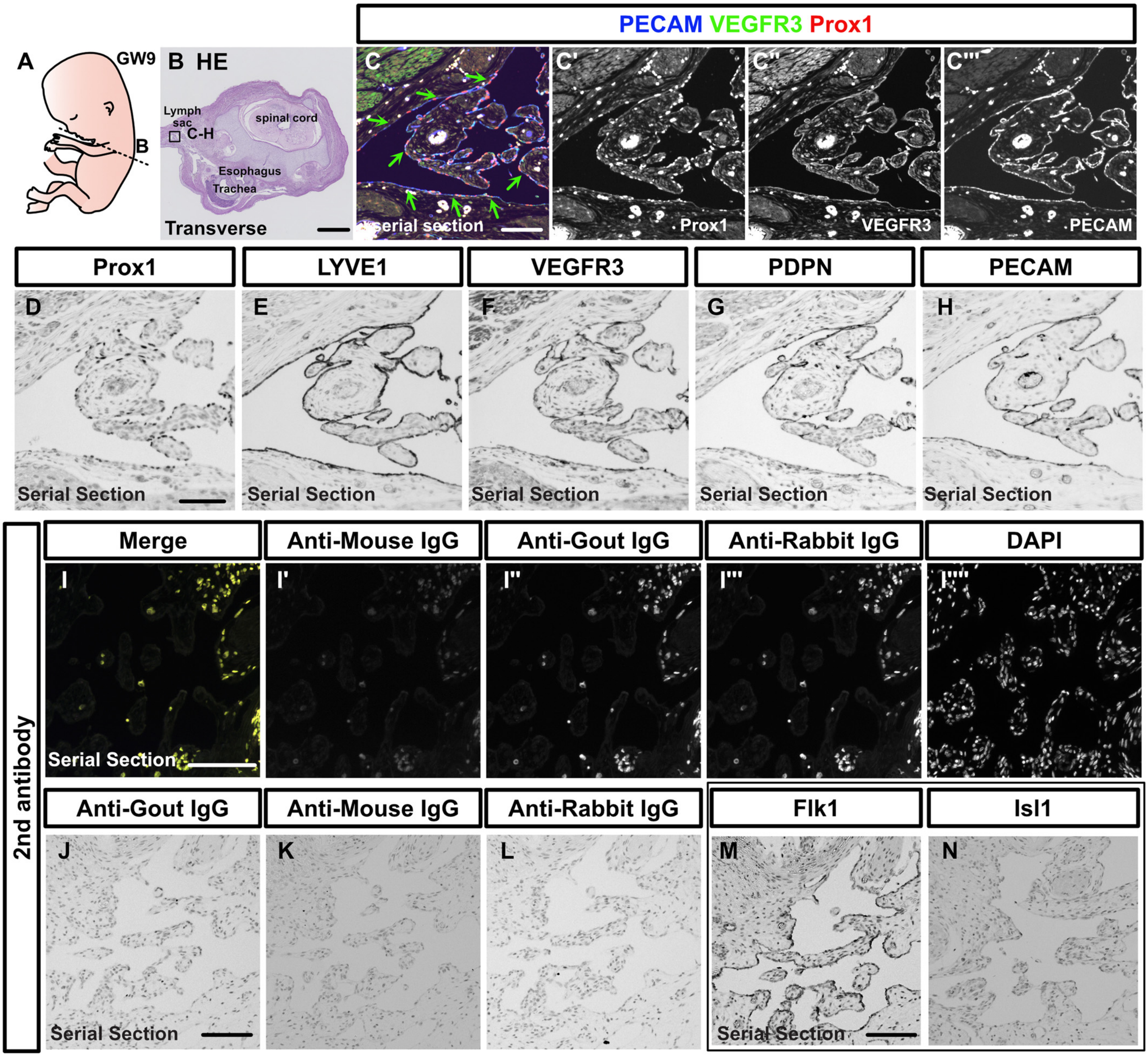

Figure EV1. Multiple lymphatic markers are expressed in fetal lymph sacs.

(**A–H**) Schema showing the positions of the sections in a GW9 fetus (**A**), and immunostaining of transverse sections (**B**–H); (**C–C‴**) Fluorescent immunostaining of PECAM, Prox1, and VEGFR3; These markers were expressed in lymph sacs (green arrows). (**D–H**) Immunostaining of Prox1, LYVE1, VEGFR3, PDPN, and PECAM using the enzyme-antibody method, with color development by DAB. (**I–L**) Imaging of the secondary antibody-only staining. (**M**, **N**) Immunostaining of Flk1, and Isl1 using the enzyme-antibody method, with color development by DAB. Scale bars, 1 mm (**B**) or 100 μm (**C**, **D**, **I**, **J**, **M**).

© The Author(s)

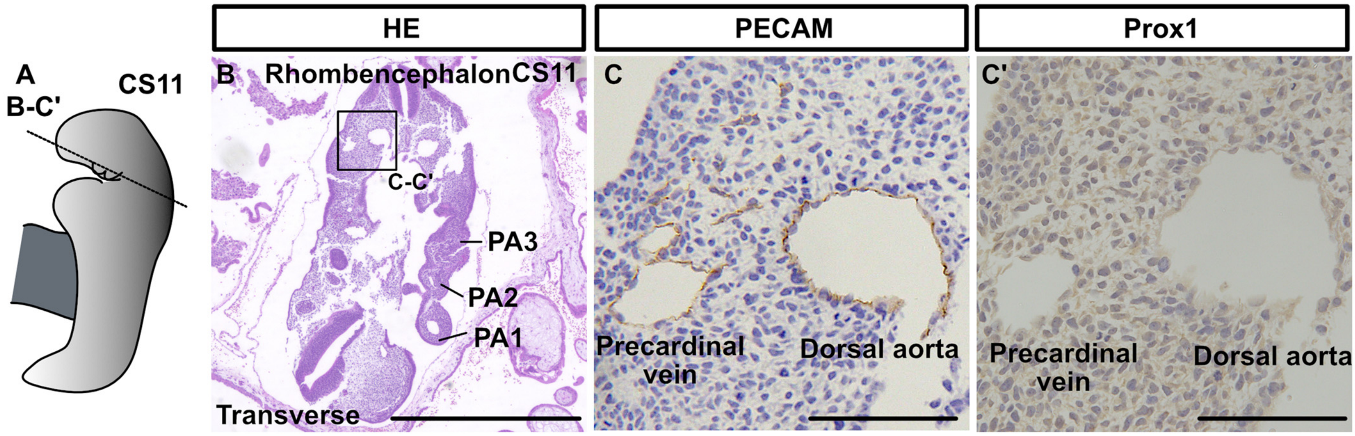

Figure EV2. Prox1 expression is not observed in the precardial vein of the CS11 embryo.

(**A–C′**) Immunostaining of transverse sections of a CS11 embryo with the indicated antibodies and schema showing a CS11 embryo ($n = 1$). Scale bars, 1 mm (**B**) or 100 μm (**C**, **C′**).

 © The Author(s)

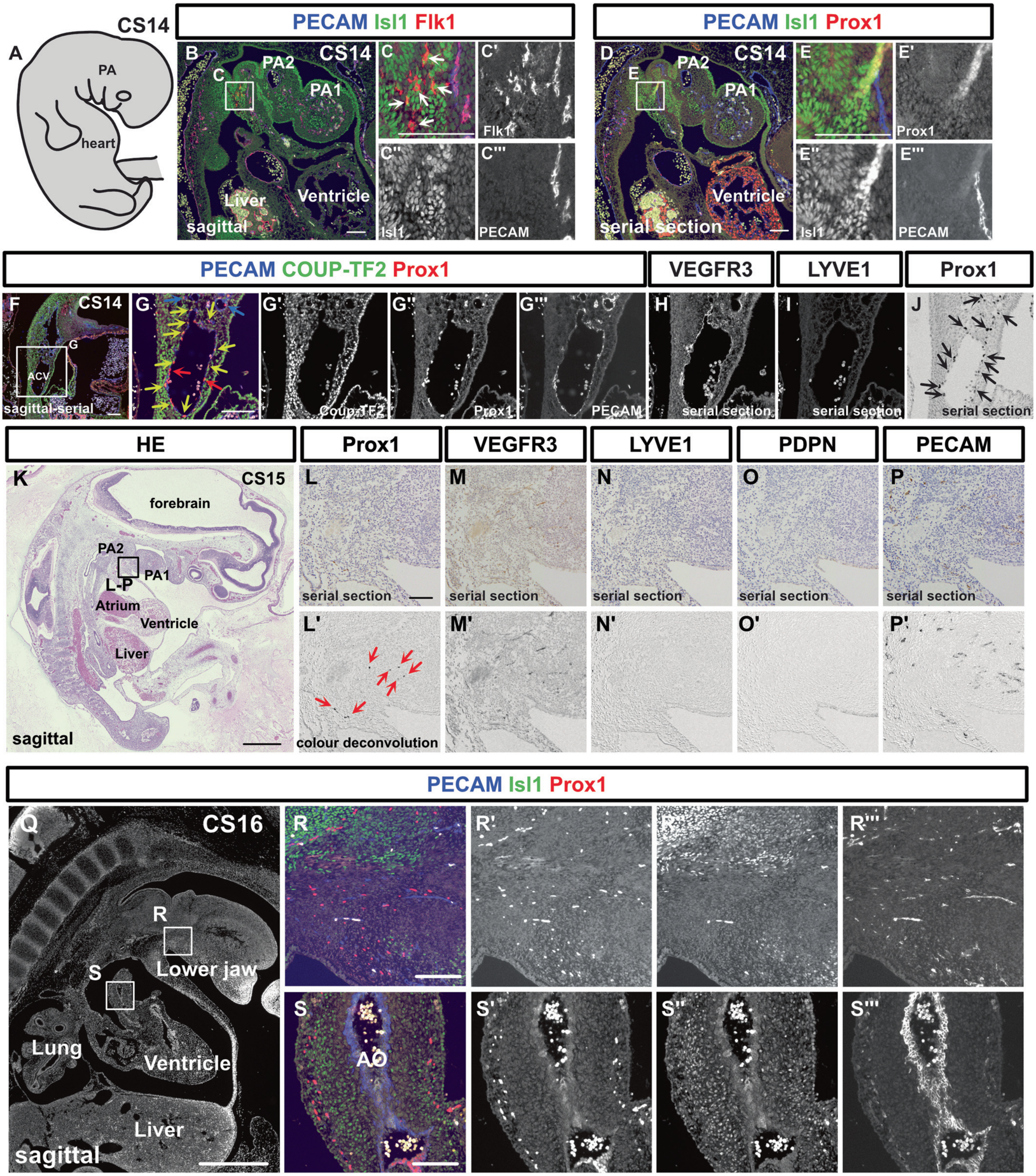
CS14
A
PA
heart
PECAM Isl1 Flk1
B
C
PA2
PA1
CS14
Liver
Ventricle
sagittal
C'
Flk1
C''
Isl1
C'''
PECAM
PECAM Isl1 Prox1
D
E
PA2
PA1
CS14
Ventricle
serial section
E'
Prox1
E''
Isl1
E'''
PECAM
PECAM COUP-TF2 Prox1
VEGFR3
LYVE1
Prox1
F
CS14
G
ACV
sagittal-serial
G'
Coup-TF2
G''
Prox1
G'''
PECAM
H
serial section
I
serial section
J
serial section
HE
Prox1
VEGFR3
LYVE1
PDPN
PECAM
K
CS15
forebrain
PA2
PA1
L-P
Atrium
Ventricle
Liver
sagittal
L
serial section
M
serial section
N
serial section
O
serial section
P
serial section
L'
colour deconvolution
M'
N'
O'
P'
PECAM Isl1 Prox1
Q
CS16
R
S
Lower jaw
Lung
Ventricle
Liver
sagittal
R'
R''
R'''
S'
S''
S'''
AO

© The Author(s)

Figure EV3. LECs bud from the cardinal veins and form luminal structures.

(**A–J**) Immunostaining of sagittal sections of a CS14 embryo with the indicated antibodies and schema showing the CS14 embryo. (**B–C‴**) Cardiovascular progenitor cells, which were composed of FLK1$^+$/Isl1$^+$/PECAM$^-$ cells, were observed in the second pharyngeal arch (white arrows). (**F–G‴**) PECAM$^+$/Prox1$^+$/Coup-TF2$^+$ cells (yellow arrows: Frequency of Coup-TF2$^+$ cells among PECAM$^+$/Prox1$^+$ cells=44.1%, $n = 1$ [the ACVs could not be identified in another embryo]), and PECAM$^+$/Prox1$^+$/Coup-TF2$^-$ cells (red arrows) were observed in and around the ACVs. (**K–P′**) HE staining of a sagittal section of a CS15 embryo (**K**) and immunostaining of sagittal sections of the same CS15 embryo with the indicated antibodies (**L–P′**); At this stage, scattered Prox1$^+$ cells were observed in the pharyngeal arch (red arrows). (**Q–S‴**) Immunostaining of sagittal sections of a CS16 embryo with the indicated antibodies. PA1 first pharyngeal arch, PA2 second pharyngeal arch, ACV anterior cardinal vein. scale bars, 1 mm (K) or 100 µm (**B–G**, **L**).

 © The Author(s)

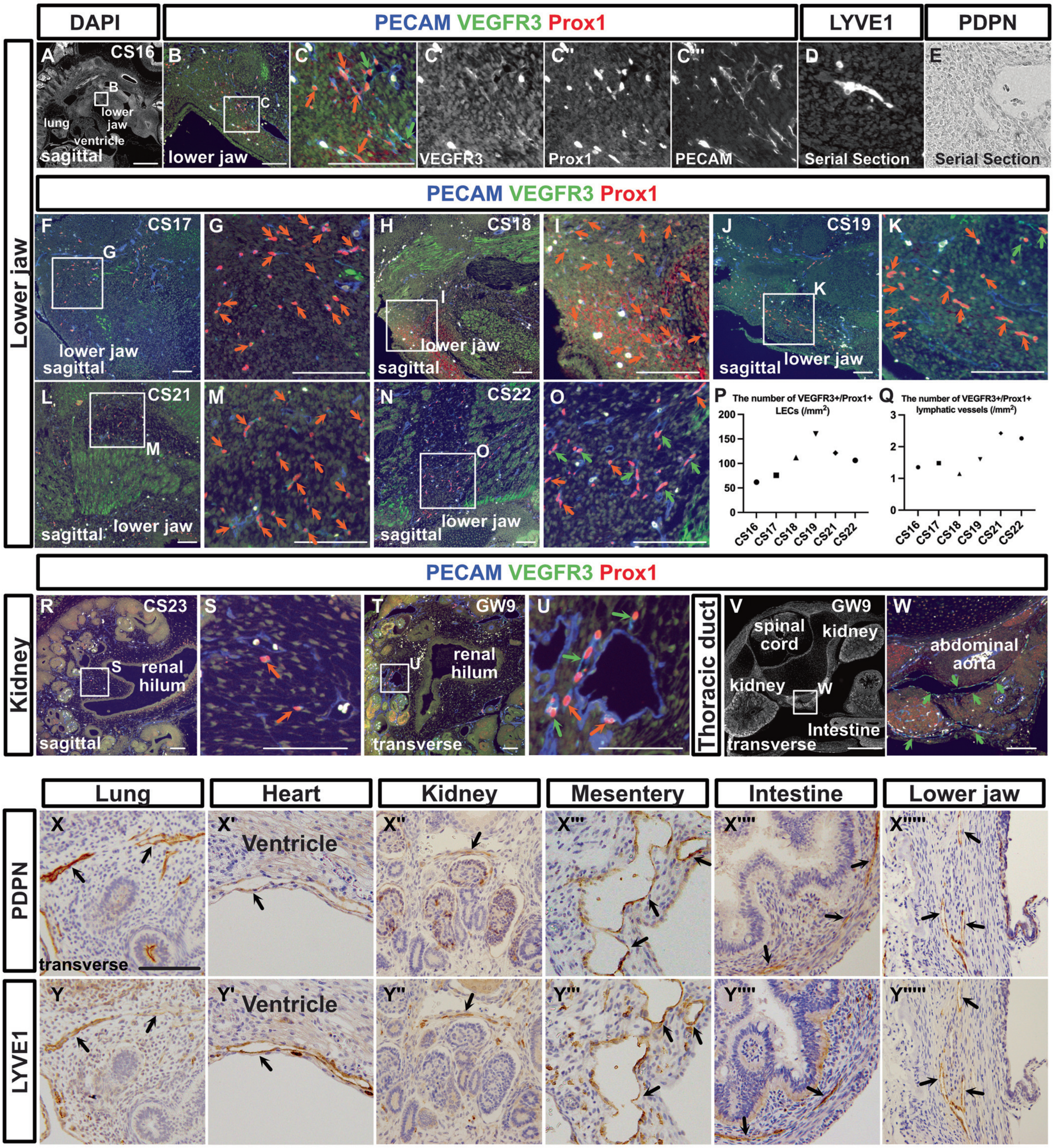

Figure EV4. Development of lymphatic vessels in the lower jaw, kidneys, and thoracic duct.

(**A–O**) Immunostaining of sagittal sections of embryos collected at the described stages with the indicated antibodies; The orange arrows indicate isolated or small clusters of LECs. The green arrows indicate lymphatic vessels that had formed luminal structures. (**P**, **Q**) Changes in the number of PECAM$^+$/Prox1$^+$/VEGFR3$^+$ LECs in the aortic wall (**P**). Changes in the number of PECAM$^+$/Prox1$^+$/VEGFR3$^+$ luminal lymphatic vessels (**Q**). Each dot represents a single individual. (**R–U**) Fluorescent immunostaining of PECAM, Prox1, and VEGFR3 in sections from embryos collected at the described stages. The orange arrows indicate isolated or small clusters of LECs. The green arrows indicate lymphatic vessels that had formed luminal structures. (**V**, **W**) The green arrows indicate lymphatic vessels surrounding the aorta at GW9. (**X**, **Y**) Immunostaining for PDPN and LYVE1 in the lung, heart, kidney, mesentery, intestine, and lower jaw. Scale bars, 1 mm (**A**, **V**) or 100 μm (**B**, **C**, **F–O**, **R–U**, **W**, **X**).

© The Author(s)

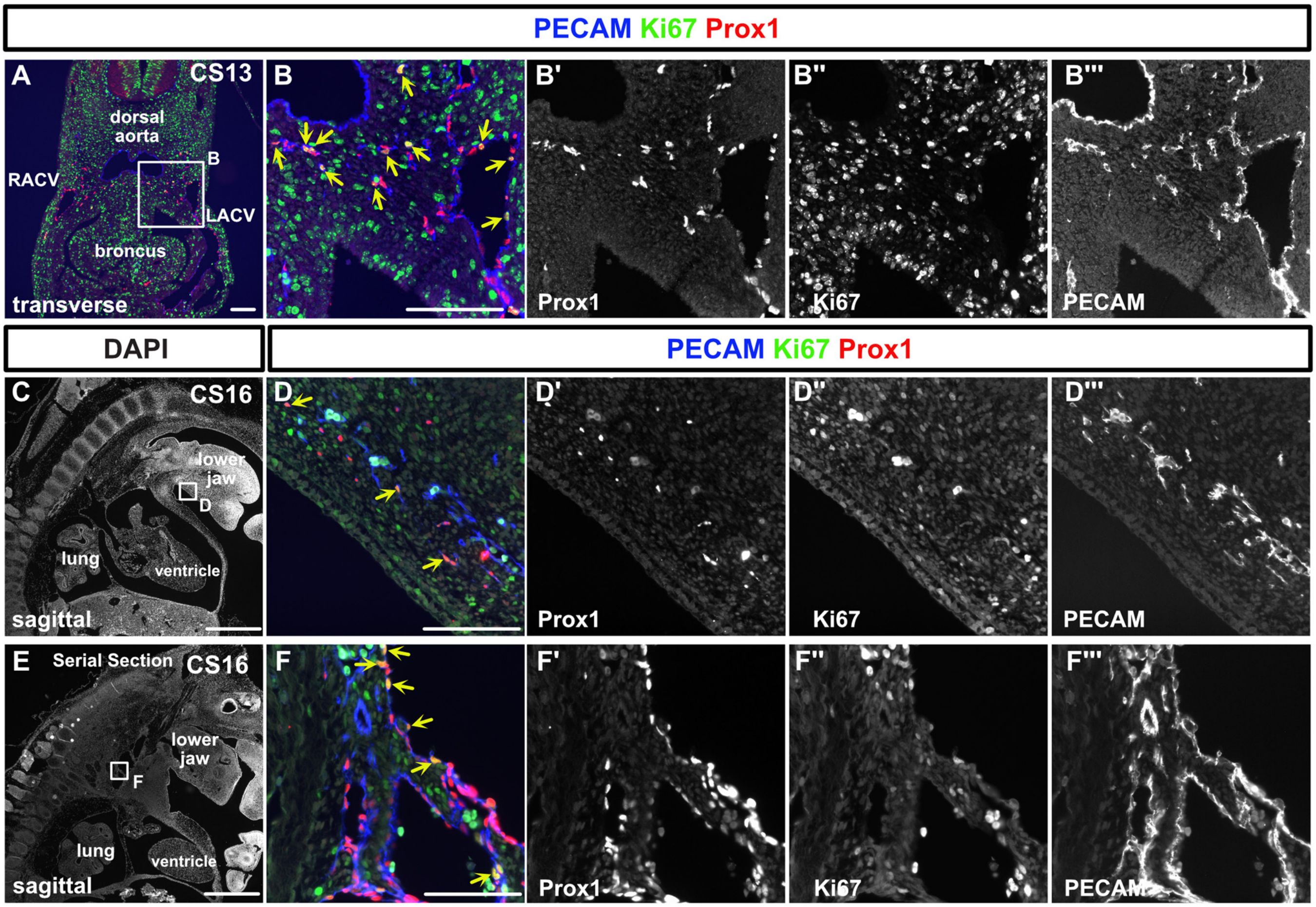

Figure EV5. Proliferation activity of lymphatic endothelial cells at CS13 and CS16.

(**A–F'''**) Immunostaining of transverse and sagittal sections using the indicated antibodies in a CS13 and 16 embryos. We detected Prox1$^+$/PECAM$^+$/Ki67$^+$ LECs (indicated by yellow arrows) as well as Prox1$^+$/PECAM$^+$/Ki67$^-$ LECs. At CS13, within CV, 19.6% of LECs were Ki67 positive, while outside the CV, the percentage was 43.6%. At CS16, 22.7% of LECs in the lymph sacs were Ki67 positive, and in the lower jaw, the positivity rate was 31% (based on an average of two sections at $n = 1$). CV cardinal vein, RACV right anterior cardinal vein, LACV left anterior cardinal vein. scale bar, 1 mm (**C**, **E**), 100 µm (**A**, **B**, **D**, **F**).

© The Author(s)
